# Microbial metabolites in chronic heart failure and its common comorbidities

**DOI:** 10.15252/emmm.202216928

**Published:** 2023-05-08

**Authors:** Sha Hua, Bomin Lv, Zeping Qiu, Zhuojin Li, Zhiyan Wang, Yanjia Chen, Yanxin Han, Katherine L Tucker, Hao Wu, Wei Jin

**Affiliations:** ^1^ Department of Cardiovascular Medicine, Heart Failure Center, Ruijin Hospital and Ruijin Hospital Lu Wan Branch Shanghai Jiao Tong University School of Medicine Shanghai China; ^2^ State Key Laboratory of Genetic Engineering, Human Phenome Institute, Fudan Microbiome Center, Department of Bariatric and Metabolic Surgery, Huashan Hospital Fudan University Shanghai China; ^3^ Department of Biomedical and Nutritional Sciences University of Massachusetts Lowell Lowell MA USA

**Keywords:** biomarkers, chronic heart failure, microbial metabolites, multimorbidity, Cardiovascular System, Metabolism

## Abstract

This study aimed to identify microbial signatures that contribute to the shared etiologies between chronic heart failure (CHF), type 2 diabetes, and chronic kidney disease. The serum levels of 151 microbial metabolites were measured in 260 individuals from the Risk Evaluation and Management of heart failure cohort, and it was found that those metabolites varied by an order of 10^5^ fold. Out of 96 metabolites associated with the three cardiometabolic diseases, most were validated in two geographically independent cohorts. In all three cohorts, 16 metabolites including imidazole propionate (ImP) consistently showed significant differences. Notably, baseline ImP levels were three times higher in the Chinese compared with the Swedish cohorts and increased by 1.1–1.6 fold with each additional CHF comorbidity in the Chinese population. Cellular experiments further supported a causal link between ImP and distinct CHF relevant phenotypes. Additionally, key microbial metabolite‐based risk scores were superior in CHF prognosis than the traditional Framingham or Get with the Guidelines‐Heart Failure risk scores. Interactive visualization of these specific metabolite‐disease links is available on our omics data server (https://omicsdata.org/Apps/REM‐HF/).

## Graphical abstract

Targeted metabolomics profiling and cellular experiments revealed that many microbially associated metabolites may mediate the pathogenesis of chronic heart failure (CHF), as well as its related comorbidities including type 2 diabetes and chronic kidney diseases.

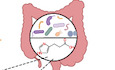

**The paper explained**

**Problem**

Whether and which circulating microbial metabolites are associated with chronic heart failure and related comorbidities is currently unknown.

**Results**

An association between microbial metabolites and cardiometabolic diseases was identified in a Chinese cohort study of 260 individuals with or without incidence of chronic heart failure and related comorbidities. Compared with the traditional heart failure risk score, the metabolite‐based risk score exhibited superior performance for CHF prognosis.

**Impact**

These results revealed several potential microbial metabolites and pathways that could be utilized for chronic heart failure multimorbidity monitoring, targeted for drug design, and integrated for disease prognosis.

## Introduction

As the most energy‐ and oxygen‐consuming organ per tissue mass, the heart is sensitive to shifts in myocardial metabolism and energetics, such as accumulation of toxic lipid intermediates and fuel shifts from free fatty acids (FFAs) to anaerobic glycolysis (Doenst *et al*, 2013; Bertero & Maack, 2018; Lopaschuk *et al*, 2021). As such, chronic heart failure (CHF) resulting from metabolic inflexibility is often accompanied by multi‐morbidities, especially type 2 diabetes (T2D) and chronic kidney disease (CKD), both of which have contributed to a 2–3 fold increase in the incidence of CHF (Nichols *et al*, 2004; Echouffo‐Tcheugui *et al*, 2022). In total, 13.7 million adults in China (Hao *et al*, 2019) and 64.3 million worldwide (Bragazzi *et al*, 2021) have been diagnosed with CHF, among which 16% were estimated to be accompanied by both T2D and CKD (Lawson *et al*, 2021). One recent epidemiological study revealed that CHF patients co‐occuring with both diseases had the worst mortality and rehospitalization (Lawson *et al*, 2021), imposing ever‐increasing social and health burdens. However, the metabolic interconnectivities and shared components among these three chronic diseases remain poorly understood, despite the fact that some classical clinical biomarkers reflecting insulin resistance (Ingelsson *et al*, 2005), hyperglycemia (Matsushita *et al*, 2010), and dyslipidemia (Tuunanen *et al*, 2006) have been implicated in the pathogenesis and development of CHF. Understanding the molecular signatures and metabolic remodeling of CHF and its associated comorbidities are, thus, fundamental to reveal the underlying pathophysiological factors and heterogenicities inherent to those cardiometabolic diseases and to develop new therapeutic targets (Seferovic *et al*, 2022) and systems biology approaches (Voors *et al*, 2016).

Recent advances in metabolomics profiling provide us with an opportunity to address this. An in‐depth molecular profiling of a large prospective cohort has been reported recently, and 420 metabolites were found to be shared by at least two noncommunicable diseases, including CHF, T2D, and/or CKD (Pietzner *et al*, 2021). Notably, a substantial proportion of the metabolites associated with the gut microbiota, such as imidazole propionate (ImP), a microbially produced metabolite that could directly lead to insulin resistance via the mTORC1‐p38γ signaling pathway (Koh *et al*, 2018). In agreement, the intestinal overgrowth of pathogenic bacteria has been observed in each of these three chronic diseases (Pasini *et al*, 2016; Yang *et al*, 2018; Tang *et al*, 2019), and microbial signatures could even be detected in atherosclerotic lesions in individuals with chronic heart disease (Ott *et al*, 2006). It is reasonable to speculate that the gut microbiome and their metabolites may mediate some of the shared etiologies among CHF and its associated comorbidities, which has led to increased efforts in identifying the microbial metabolites responsible for these cardiometabolic diseases (Brown & Hazen, 2018; Canfora *et al*, 2019).

Here, we performed a targeted quantification of 151 microbially associated metabolites in 260 individuals from the Risk Evaluation and Management of Heart Failure (REM‐HF) cohort, with defined combinations of diabetes, cardiac, and renal status. We aimed to investigate: (i) if previously identified microbial metabolites associated with T2D, CHF, and/or CKD based on untargeted metabolic profiling could be validated using absolute quantification in an independent Chinese CHF cohort with rising burdens from different cardiometabolic comorbidities; (ii) which clinical variables mediate the associations between those microbial metabolites and diseases; and (iii) if the circulating levels of specific microbial metabolites contributed to disease severity and prognosis.

## Results

### Clinical characteristics

**Figure EV1:**
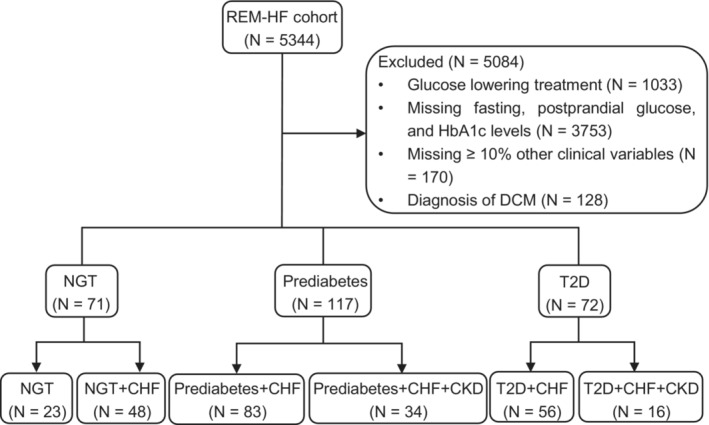
CONSORT diagram for REM‐HF cohort CHF, chronic heart failure; CKD, chronic kidney disease; DCM, Dilated cardiomyopathy; NGT, normal glucose tolerance; T2D, type 2 diabetes.

Characterization of prediabetes or intermediate hyperglycaemia presents a unique opportunity for studying the role of gut microbiota in the progression to clinical T2D. We and others have demonstrated the potential contribution of gut microbiota in this transition (Zhou *et al*, 2019; Wu *et al*, 2020). To further explore the molecular signatures that may link CHF and T2D development, we screened 260 individuals with varying glucose metabolism from the REM‐HF cohort and conducted a targeted metabolomics analysis. The individuals could be classified into six subgroups, including individuals with normal glucose tolerance (NGT), which served as the control group, NGT concomitant with CHF (NGT + CHF), prediabetes with CHF (Prediabetes + CHF), prediabetes with both CHF and CKD (Prediabetes + CHF + CKD), T2D with CHF (T2D + CHF), and T2D with both CHF and CKD (T2D + CHF + CKD) (Fig EV1). A general description of the clinical variables is summarized in Table 1. Individuals with prediabetes and diabetes were, in general, older and tended to suffer from cardiac and renal dysfunctions, but no differences in body mass index (BMI) or blood pressures were observed. Serum albumin, a biomarker for protein nutritional status, was reduced in most CHF patients, while blood urea nitrogen (BUN) was increased in parallel with poorer kidney function, as reflected in differences in eGFR across groups.

**Table 1:** Characteristics of the REM‐HF cohort.

	NGT (*N* = 23)	NGT + CHF (*N* = 48)	Prediabetes + CHF (*N* = 83)	T2D + CHF (*N* = 56)	Prediabetes + CHF + CKD (*N* = 34)	T2D + CHF + CKD (*N* = 16)
Age (years)	53.61 ± 2.91	60.23 ± 2.27	61.83 ± 1.38*	62.38 ± 1.47*	72.06 ± 2.16^#^	72.50 ± 2.68^#^
Sex (female %)	47.83	38.78	18.07^+^	19.64*	41.18	43.75
BMI (kg/m^2^)	23.92 ± 0.82	23.53 ± 0.53	25.28 ± 0.47	25.29 ± 0.53	23.25 ± 0.67	24.85 ± 0.95
SBP (mmHg)	127.83 ± 3.46	127.19 ± 3.07	132.88 ± 2.40	130.79 ± 3.62	130.65 ± 4.71	132.12 ± 5.81
DBP (mmHg)	75.13 ± 2.41	71.54 ± 1.91	77.72 ± 1.75	77.02 ± 1.97	74.38 ± 3.16	78.25 ± 2.57
Albumin (g/l)	40.26 ± 0.65	37.60 ± 0.65*	36.83 ± 0.57^+^	37.36 ± 0.58^+^	34.50 ± 0.93^#^	36.69 ± 1.33*
hsCRP (mg/l)	2.90 ± 1.38	4.18 ± 1.38	11.71 ± 3.09	15.24 ± 3.86^+^	23.99 ± 10.77^#^	6.05 ± 1.65*
Lymphocyte (×10^9^/l)	1.89 ± 0.09	2.40 ± 0.80	1.72 ± 0.07	1.22 ± 0.07*	1.65 ± 0.08^#^	1.44 ± 0.15^+^
White blood cells (×10^9^/l)	5.95 ± 0.33	7.17 ± 0.87	7.02 ± 0.24*	7.55 ± 0.32^+^	6.26 ± 0.40	7.18 ± 0.57
**Lipid metabolism (mmol/l)**						
FFA	0.45 ± 0.04	0.44 ± 0.03	0.54 ± 0.02*	0.59 ± 0.03^+^	0.63 ± 0.04^+^	0.77 ± 0.08^+^
HDLc	1.24 ± 0.07	1.17 ± 0.04	1.06 ± 0.03*	1.10 ± 0.04	1.02 ± 0.05*	1.05 ± 0.09
LDLc	2.89 ± 0.19	2.54 ± 0.12	2.52 ± 0.09	2.47 ± 012	2.37 ± 0.18*	1.78 ± 0.14^#^
TG	1.41 ± 0.11	1.42 ± 0.08	1.33 ± 0.07	1.55 ± 0.15	1.10 ± 0.06*	1.26 ± 0.10
TC	4.59 ± 0.24	4.19 ± 0.14	4.03 ± 012*	4.07 ± 0.16	3.72 ± 0.22^+^	3.17 ± 0.20^#^
**Glucose metabolism (mmol/l)**						
Fasting glucose	5.03 ± 0.10	4.75 ± 0.07*	5.49 ± 0.08^+^	7.61 ± 0.41^#^	5.76 ± 0.14^#^	7.34 ± 0.66^#^
2 h Glucose	6.02 ± 0.16	6.19 ± 0.13	8.89 ± 0.13^#^	12.95 ± 0.47^#^	8.50 ± 0.24^#^	13.82 ± 1.04^#^
Fasting insulin (pmol/l)	7.44 ± 0.64	7.71 ± 1.03	8.97 ± 0.62	17.75 ± 4.85	8.11 ± 0.77	15.48 ± 3.37*
2 h Insulin (pmol/l)	28.47 ± 4.06	40.42 ± 5.02	76.10 ± 6.69^#^	84.56 ± 14.37^#^	66.32 ± 10.23^+^	102.35 ± 26.19^#^
HbA1c (%)	5.39 ± 0.04	5.35 ± 0.04	6.12 ± 0.07^#^	7.23 ± 0.19^#^	5.90 ± 0.08^#^	7.00 ± 0.29^#^
HOMA‐IR	1.68 ± 0.16	1.67 ± 0.24	2.24 ± 0.16	5.96 ± 1.42^+^	2.11 ± 0.22	5.90 ± 2.20^+^
QUICKI	0.16 ± 0.003	0.16 ± 0.003	0.15 ± 0.002	0.14 ± 0.003^+^	0.15 ± 0.003	0.14 ± 0.004^+^
Adipose‐IR	3.25 ± 0.35	3.11 ± 0.34	4.92 ± 0.38*	8.65 ± 2.22^+^	5.02 ± 0.60	12.36 ± 3.43^+^
TyG index	8.57 ± 0.08	8.51 ± 0.06	8.57 ± 0.05	8.94 ± 0.08^+^	8.47 ± 0.07	8.81 ± 0.11
**Heart function**						
LVEF (%)	65.78 ± 1.36	44.81 ± 1.89^#^	42.55 ± 1.35^#^	41.16 ± 1.711^#^	46.62 ± 2.30^#^	44.12 ± 3.62^#^
NT‐proBNP (μg/l)	0.31 ± 0.14	2.52 ± 0.46^#^	2.88 ± 0.45^#^	2.93 ± 0.62^#^	7.74 ± 1.68^#^	5.18 ± 1.50^#^
Myoglobin (ng/ml)	21.87 ± 2.51	35.07 ± 2.74^#^	36.52 ± 2.65^#^	116.61 ± 70.56^+^	83.21 ± 14.94^#^	54.94 ± 6.65^#^
TnI (μg/l)	0.59 ± 0.55	0.10 ± 0.04*	0.55 ± 0.37^+^	1.63 ± 0.98^+^	3.30 ± 2.87^#^	0.24 ± 0.17^+^
**Kidney function**						
BUN (mmol/l)	5.19 ± 0.30	5.97 ± 0.26	6.56 ± 0.20^+^	6.75 ± 0.29^#^	11.56 ± 1.09^+^	10.51 ± 0.88^#^
Creatinine (μmol/l)	69.43 ± 2.95	79.45 ± 2.49*	84.95 ± 1.76^#^	80.09 ± 2.42^+^	160.12 ± 25.79^#^	128.06 ± 7.86^#^
CystatinC (mg/l)	0.93 ± 0.03	1.17 ± 0.04^#^	1.23 ± 0.03^#^	1.14 ± 0.03^#^	1.98 ± 0.20^#^	1.72 ± 0.12^#^
eGFR (ml/min/1.72 m^2^)	96.31 ± 2.70	86.97 ± 2.53^+^	82.36 ± 1.60^#^	85.23 ± 2.73^+^	42.02 ± 2.58^#^	46.42 ± 2.72^#^
Uric acid (μmol/l)	316.91 ± 13.92	400.45 ± 15.60^#^	412.23 ± 13.61^#^	399.73 ± 16.39^+^	498.74 ± 32.60^#^	487.62 ± 34.15^#^

Values reported as mean ± SE. For continuous variables, the Wilcox rank‐sum test was used. For categorical variables, the chi‐squared test was used. *P < 0.05, ^+^ *P* < 0.01, ^#^ *P* < 0.001 versus the NGT group. Adipose‐IR, adipose insulin resistance; BUN, blood urea nitrogen; DBP, diastolic blood pressure; eGFR, estimated glomerular filtration rate; FFA, free fatty acid; HbA1C, glycated hemoglobin; HDLc, high‐density lipoprotein cholesterol; HOMA‐IR, homeostatic model assessment of insulin resistance; hsCRP, high‐sensitivity c‐reactive protein; LDLc, low‐density lipoprotein cholesterol; LVEF, left ventricular ejection fraction; NT‐proBNP, N‐terminal pro‐B‐type natriuretic peptide; QUICKI, quantitative insulin sensitivity check index; SBP, systolic blood pressure; TC, total cholesterol; TG, triglyceride; TnI, troponin I; TyG index, triglyceride‐glucose index.

### Absolute quantification of the microbially associated metabolites

**Figure EV2:**
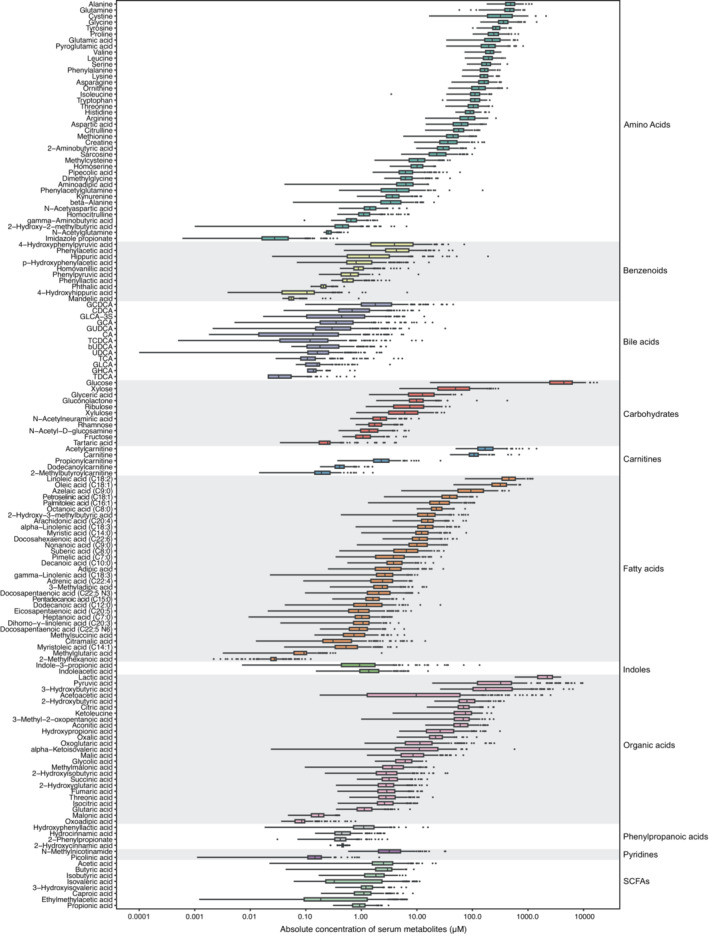
Absolute plasma levels of all 151 microbially associated metabolites in REM‐HF cohort The central band in each box represents the median, the top, and bottom of the box the 25^th^ and 75^th^ percentiles, and the whiskers 1.5 times the interquartile range (*n* = 260; biological replicates).

**Figure EV3:**
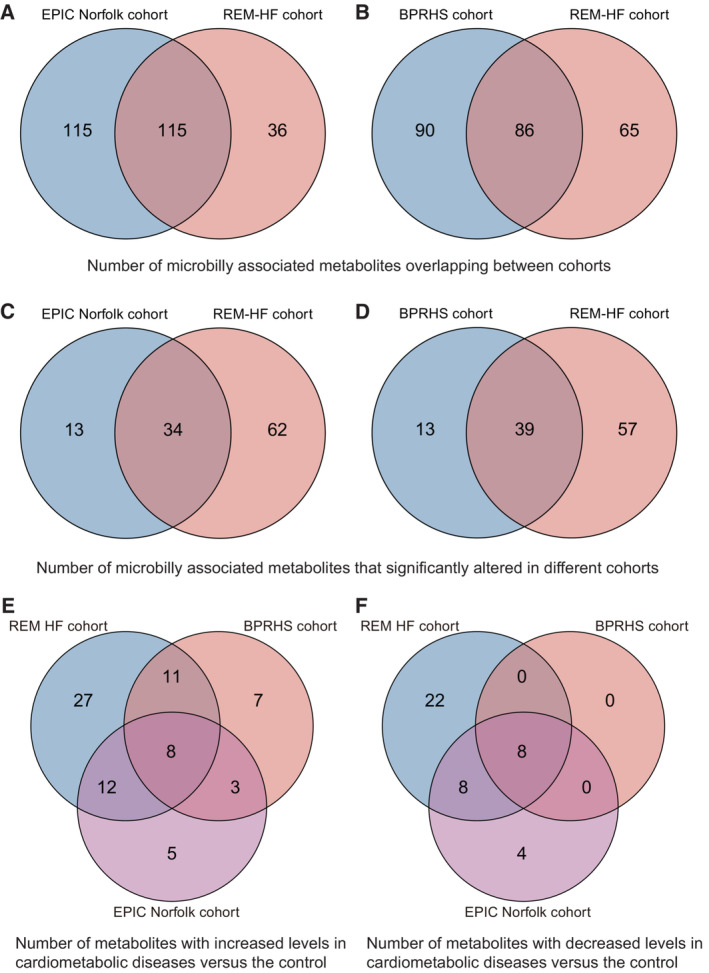
Venn plots depicting numbers of shared metabolites across cohorts A–FNumbers of metabolites (A) and significantly changed metabolites (C) that were overlapping between REM‐HF and EPIC‐Norfolk cohorts and similarly for those (B, D) between REM‐HF and BPRHS cohorts. Numbers of increased (E) and decreased (F) metabolites across all three cohorts, respectively.

The targeted metabolomics profiling of all collected serum samples from this cohort was carried out on a Waters ACQUITY ultraperformance liquid chromatography platform coupled with a Waters XEVO TQ‐S mass spectrometry (UPLC‐MS/MS) system (Xie *et al*, 2021). A total of 199 metabolites were quantified, of which 151 (75.9%) were identified as being microbially associated, including host‐bacteria cometabolites (see Materials and Methods for definition), while 48 metabolites were potentially host‐specific (Table EV1). Consistent with others (Zhou *et al*, 2019), most known metabolites belong to amino acids and lipids metabolism (Fig EV2) but varied by an order of 10^5^ fold on average. A revisit of the metabolic profiles of the European Prospective Investigation into Cancer (EPIC)‐Norfolk (Pietzner *et al*, 2021) and the Boston Puerto Rican Health Study (BPRHS) cohorts (Murthy *et al*, 2020) revealed that 230 and 176 microbial metabolites could be defined according to the same standards, respectively, which served as the validation datasets. Out of the 151 microbial metabolites quantified in this study, 115 (76.2%) and 86 (57.0%) overlap with these two geographically independent cohorts, respectively (Fig EV3A and B).

### Associations with CHF and associated comorbidities

**Figure 1:**
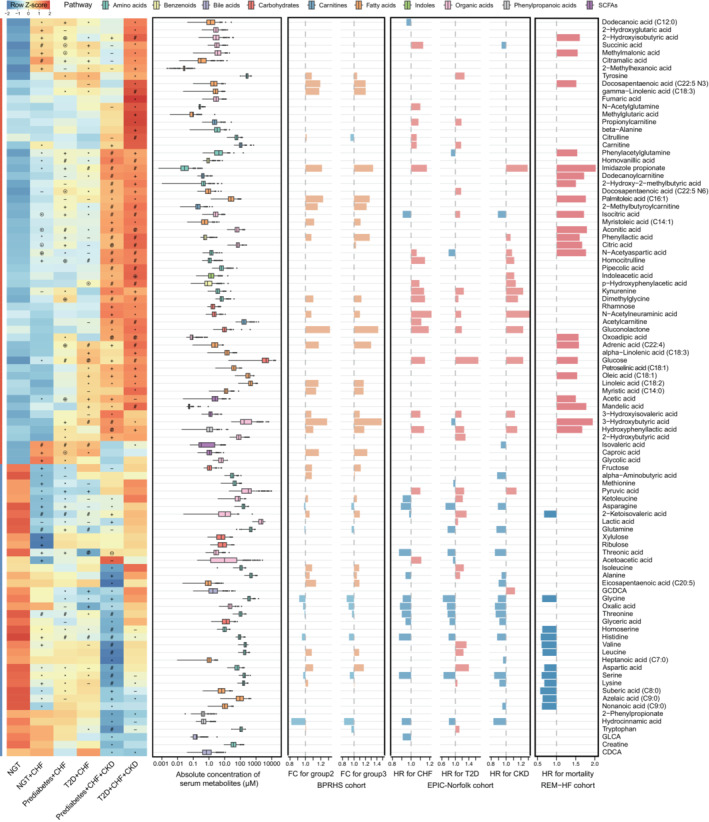
Microbially associated metabolites altered in CHF and its comorbidities *First panel*: the heatmap indicating the mean serum concentrations (scaled by row) of 96 metabolites in the REM‐HF cohort. *P* values were calculated by the Wilcoxon rank‐sum test. Symbols indicate the raw *P* values (^−^ *P* < 0.1, *P < 0.05, ^+^ *P* < 0.01, ^#^ *P* < 0.001), and symbols with open circles indicate metabolite‐disease links that were potentially confounded by medications. *Second panel*: the boxplot showing the absolute serum concentrations of the corresponding metabolites with box colors for different pathway annotations. The central band in each box represents the median, the top, and bottom of the box the 25^th^ and 75^th^ percentiles, and the whiskers 1.5 times the interquartile range (*n* = 260; biological replicates). *Third panel*: the bar plot showing the fold‐changes (FC) of 39 metabolites associated with the cardiometabolic stress scores (group 1–3) in the BPRHS cohort. *Forth panel*: the bar plot showing the hazard ratio (HR) of each metabolite for the incidence of HF, T2D, or CKD identified in the EPIC‐Norfolk cohort. *Last panel*: the bar plot showing the hazard ratios (HRs) of 33 metabolites for CHF rehospitalization or cardiovascular death in the REM‐HF cohort. The HRs were calculated with Cox proportional hazards models, adjusting for age, sex, and BMI (likelihood‐ratio test *P* < 0.05 and Schoenfeld residuals test *P* > 0.05).Source data are available online for this figure.

Compared with the NGT controls, the circulating levels of 94 microbial metabolites were significantly altered in at least one disease setting, after adjusting for age and sex differences (adjusted *P* value < 0.1). Additionally, two metabolites, myristic acid (C14:0) and 2/alpha‐aminobutyric acid, showed altered concentrations based only on the raw *P* values, but with supporting evidence from the validation cohorts (Table EV2). In total, we identified 258 metabolite‐disease links from 96 microbially associated metabolites, among which 23 such links from 19 metabolites were potentially confounded by medications based on a state‐of‐the‐art drug‐deconfounding pipline (Forslund *et al*, 2021; Fig 1; Table EV2). For instance, associations between threonic acid and CHF and/or related comorbidities tend to be affected by diuretics, consistent with previous findings (Forslund *et al*, 2021).

Forty‐seven microbial metabolites were reported to be consistently correlated with the incidence of T2D, CHF, or CKD in two EPIC‐Norfolk subcohorts, based on the reported raw *P* values (age and sex‐adjusted; Pietzner *et al*, 2021), with 72.3% (34/47) aligning with our findings (Fig EV3C). Of note, 75% (39/52) of metabolites that associated with the cardiometabolic stress index defined in the BPRHS cohort (Murthy *et al*, 2020) were also observed with altered circulating levels here (Fig EV3D), consolidating the suggestion that shared pathophysiologies exist among these three common cardiometabolic diseases. We additionally identified 49 new metabolite‐disease links, with 27 and 22 associated with increased and decreased disease risks, respectively (Fig EV3E and F; Table EV3). Of these, the abundances of eight metabolites, including hydrocinnamic acid, were consistently reduced in all three cohorts, and that of another eight with reduced concentrations including glyceric acid were validated in the EPIC‐Norfolk cohort only, implying that those metabolites may play protective roles against CHF and/or related comorbidities. In aggreement, glyceric acid supplementation was shown recently in a clinical trial to be able to alleviate metabolic disorders by boosting mitochondrial metabolism (Hirvonen *et al*, 2021).

Hierarchical clustering analyses showed that all 96 altered microbial metabolites in our study could be classified into two distinct clusters (Fig 1): metabolites exhibiting increased or decreased cardiometabolic stress (group 2/3 versus group 1 defined in the BPRHS cohort, see Materials and Methods) and hazard ratios for incidence of CHF, T2D, or CKD (calculated in the EPIC‐Norfolk cohort). Notably, the observed shifts toward these three cardiometabolic diseases in all three cohorts were generally consistent. Moreover, in the REM‐HF cohort, we found that after an average of 2.3 years of follow‐up, 21 and 12 metabolites were observed with increased and decreased hazard ratios for 113 cardiovascular deaths or CHF rehospitalization events after adjusting for age, sex, and BMI, respectively. In total, the serum levels of 18 metabolites (Table EV4) were found significantly altered in all three diseases and robustly associated with CHF multimorbidity and mortality/rehospitalization.

### Metabolites from amino acid metabolism

We observed reduced circulating levels of 15 out of 19 commonly detected amino acids (missing for cysteine), including three branched‐chain amino acids (BCAAs), and a marginal significance for phenylalanine (raw *P* = 0.03; adjusted *P* = 0.14), in different disease groups compared with NGT controls. No significant differences were observed for glutamate, proline, or arginine, while increased tyrosine levels were potentially due to phenylalanine metabolism, given their significant correlation and metabolic connections (Pearson correlation coefficient *R* = 0.43, *P* = 8.6e‐13). Conversely, all seven metabolites derived from BCAAs (such as 2/alpha‐hydroxyisobutyric acid) and seven out of 12 from phenylalanine metabolism detected in this study showed increased circulation. Indoleacetic acid (IAA) and kynurenine, which result from tryptophan metabolism, were also found to have higher concentrations and have been previously linked to obesity and diabetes (Laurans *et al*, 2018). In addition, individuals with CHF and related comorbidities showed dysregulation of the cysteine‐glutathione pathway and oxidative stress, as reflected by decreased levels of methionine and two closely related intermediates from the cysteine biosynthesis pathway, namely 2/alpha‐aminobutyric acid and homoserine, and increased levels of dimethylglycine and 2/alpha‐hydroxybutyric acid. Methylmalonic acid, an alternative biomarker for oxidative stress, was also increased in individuals with CHF.

### Metabolites from lipids metabolism

Most lipid‐derived metabolites were insignificant in individuals with CHF compared with the NGT control group but showed progressively stonger patterns along with co‐ocurring CHF morbidities. For example, we noted that the serum levels for most long‐chain fatty acids, including the two most abundant ones, C18:1 (oleic acid) and C18:2 (linoleic acid) that can be taken up by the heart, were increased in the CHF + prediabetes/T2D groups but showed the most pronounced increase in individuals with all three diseases (Fig 1). C18:1 (oleic acid) and C22:4 (adrenic acid) additionally showed higher hazard ratios for incidence of CHF rehospitalization or cardiovascular deaths. Most medium‐chain fatty acids instead were observed to have reduced levels in different disease groups, such as nonanoic acid (C9:0), azelaic acid (C9:0), and suberic acid (C8:0), in line with their generally protective roles (Labarthe *et al*, 2008; Nagao & Yanagita, 2010). Carnitines and three acyl‐carnitines, including acetylcarnitine, propionylcarnitine, and 2‐methylbutyroylcarnitine, as well as the main ketone body 3/beta‐hydroxybutyric acid, which contribute about 15% of myocardial carbon uptake (Murashige *et al*, 2020), also showed significant increases in at least one disease group. Among short‐chain fatty acids, only acetic acid was found to be significantly increased in all disease groups.

### Cross‐group comparison and disease‐specific metabolites

**Figure 2:**
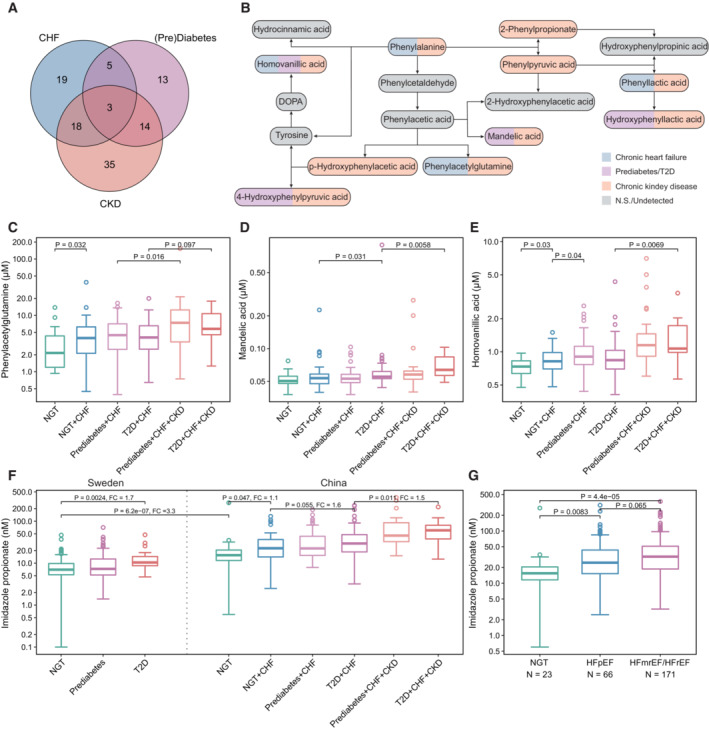
Metabolic distinctiveness among all three cardiometabolic diseases AVenn diagram showing the shared and unique metabolites associated with CHF, T2D, and CKD (Wilcoxon rank‐sum test, *P* < 0.05; ImP with a marginal signicance level (*P* = 0.055) was also included).BThe phenylalanine metabolism pathway. The color code of each metabolite shows their associations with different diseases.C–EBoxplots showing the serum level of phenylacetylglutamine, mandelic acid and homovanillic acid in different CHF subgroups in the REM‐HF cohort. NGT: *n* = 23; NGT + CHF: *n* = 48; Prediabetes+CHF: *n* = 83; T2D + CHF: *n* = 56; Prediabetes + CHF + CKD: *n* = 34; T2D + CHF + CKD: *n* = 16 (biological replicates).FThe boxplot showing the serum level of ImP in the Swedish prediabetes versus the Chinese REM‐HF cohorts. For Swedish prediabetes cohort, NGT: *n* = 125; prediabetes: *n* = 262; T2D: *n* = 17 (biological replicates). For Chinese REM‐HF cohort: NGT: *n* = 23; NGT + CHF: *n* = 48; Prediabetes+CHF: *n* = 83; T2D + CHF: *n* = 56; Prediabetes+CHF + CKD: *n* = 34; T2D + CHF + CKD: *n* = 16.GDifferences in ImP when stratified by left ventricular ejection fraction (LVEF; two‐tailed Wilcoxon rank‐sum tests). NGT: *n* = 23; HFpEF: LVEF ≥ 50, *n* = 66; HFmEF: 40 < LVEF < 50, *n* = 68; HFrEF: LVEF ≤ 40, *n* = 103 participants. Data information: the central band in each box represents the median, the top, and bottom of the box the 25^th^ and 75^th^ percentiles, and the whiskers 1.5 times the interquartile range. Wilcoxon rank‐sum test was used for all group comparisons. Source data are available online for this figure.

**Figure EV4:**
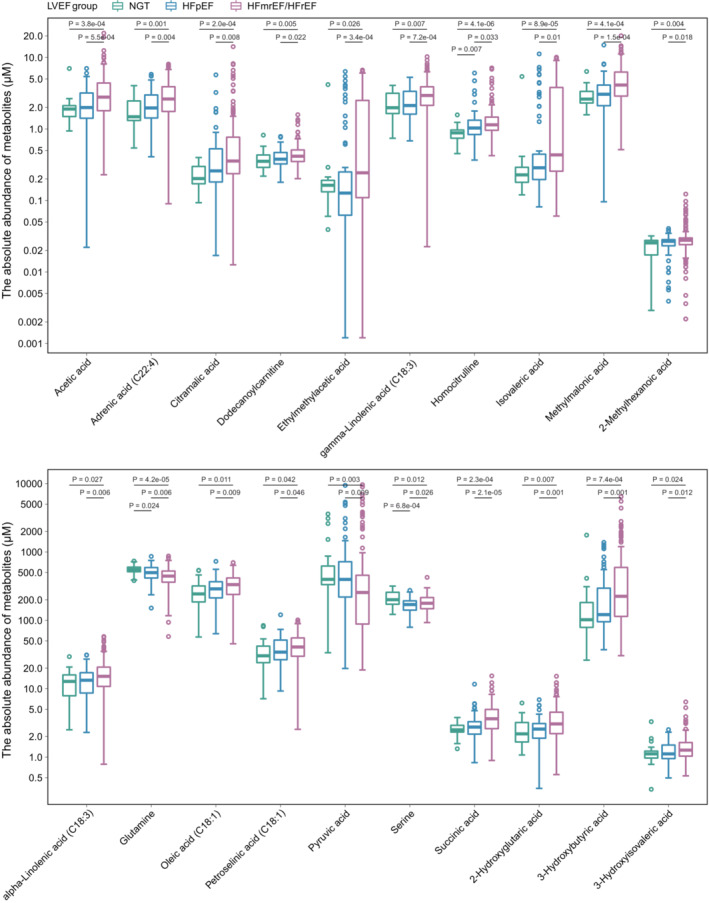
Metabolites that differed between different LVEF groups in REM‐HF cohort The central band in each box represents the median, the top, and bottom of the box the 25^th^ and 75^th^ percentiles, and the whiskers 1.5 times the interquartile range. NGT: *n* = 23, HFpEF: LVEF ≥ 50, *n* = 66; HFmEF: 40 < LVEF < 50, *n* = 68; HFrEF: LVEF ≤ 40, *n* = 103 (biologcial replicates).

We additionally performed cross‐group comparisons to identify potential disease‐specific metabolites. Our results revealed that 19, 13, and 35 metabolites were unique to CHF (NGT + CHF versus NGT), prediabetes/T2D (Prediabetes/T2D + CHF versus NGT + CHF), and CKD (Prediabetes/T2D + CHF + CKD versus Prediabetes/T2D + CHF), respectively. Three metabolites, including ImP, were common among all three diseases (Fig 2A). Notably, our results implied that metabolites from the same pathway, such as those from phenylalanine metabolism, might have distinct roles in the pathogenesis and development of different cardiometabolic diseases (Fig 2B). For example, phenylacetylglutamine, a microbially produced metabolite that had been linked with incidence of major cardiovascular events (Nemet *et al*, 2020), was confirmed here and associated with both CHF and CKD but not T2D, mandelic acid with T2D and CKD but not CHF, p/4‐hydroxyphenylacetic acid (4‐HPA) with CKD only, and homovanillic acid instead increased with all three diseases (Fig 2B–E). In support, 4‐HPA, a known uremic toxin tightly connected with CKD (Deguchi *et al*, 2002), did not affect left ventricular ejection fraction (LVEF) or myocardial infarction, the most common cause of CHF (Zhou *et al*, 2022). We further found that ImP increased 1.1–1.6 times with the occurrence of each additional CHF comorbidity, and that its absolute serum levels in Chinese were three times that in Swedish adults, even in those with NGT (Koh *et al*, 2018; Fig 2F), in parallel with a nearly threefold higher prevalence of T2D in Chinese (12.8%; Li *et al*, 2020) than Swedish adults (4.4%; Norhammar *et al*, 2016); comparable baseline characteristics including male/female ratio, average age, and BMI were found between two NGT groups (55/70, 57.6, and 25.5 in Swedish and 12/11, 53.6, and 23.9 in Chinese NGT groups, respectively). Moreover, we observed a trend (*P* = 0.065) toward higher ImP levels in individuals with reduced/moderate (HFrEF/HFmEF) than those with preserved LVEF (HFpEF) (Fig 2G). Twenty other metabolites showed significant changes between HFrEF/HFmEF and HFpEF (Fig EV4), supporting that distinct pathophysiologic features exist between those two CHF subgroups (Zordoky *et al*, 2015; Murashige *et al*, 2020).

### Validation of the metabolite‐CHF links in cardiomyoblasts

**Figure 3:**
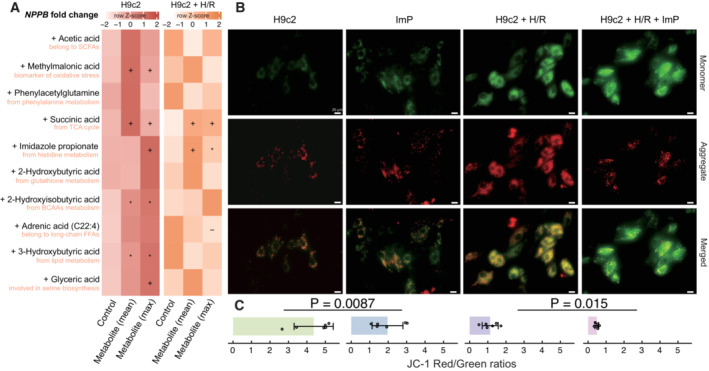
Cardiomyoblast‐metabolite coculturing and induction of *NPPB* gene expression AThe relative expression of *NPPB* (scaled by row) upon different metabolites exposure in H9c2 cells pretreated with or without hypoxia/reoxygenation (H/R; six replicates per group). Symbols indicate the raw *P* values (^−^ *P* < 0.1, *P < 0.05, ^+^ *P* < 0.01) calculated by the Wilcoxon rank‐sum test.BRepresentative JC‐1 staining images showing red fluorescence of JC‐1 aggregates and green signal of monomers. Scale bars: 20 μm.CQuantification of mitochondrial membrane potential (*n* = 6, biological replicates). The data are shown as mean ± s.d.; Wilcoxon rank‐sum test (*P < 0.05, ^+^ *P* < 0.01). Source data are available online for this figure.

**Figure EV5:**
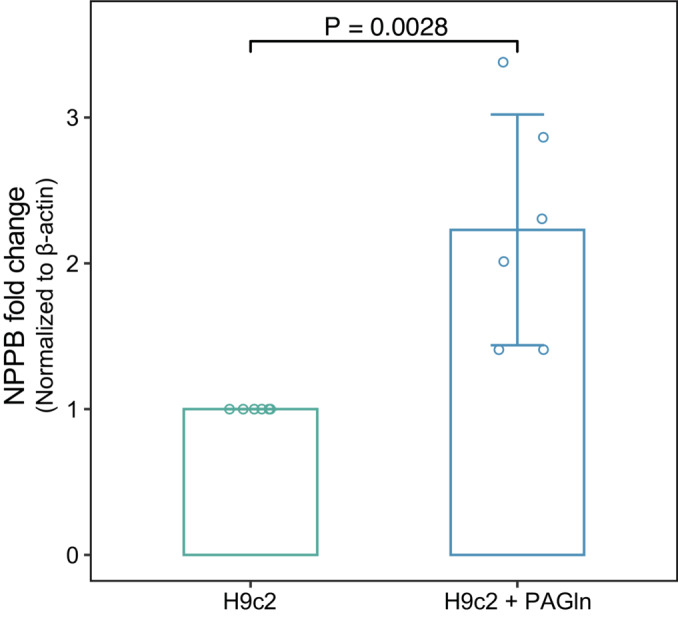
The relative expression of *NPPB* upon phenylacetylglutamine exposure The relative expression of *NPPB* was detected by RT‐PCR after 6‐h exposure to 100 μM phenylacetylglutamine (*n* = 6, biological replicates). The data are shown as mean ± s.d. Statistical analysis was performed by the Wilcoxon rank‐sum test.

Differential expression analysis of Natriuretic Peptide B gene (*NPPB*), one of the key genes relevant for the pathogenesis of CHF, was investigated in H9c2 cardiomyoblasts (derived from rat heart tissue) by exposing them to different metabolites. Ten metabolites representing distinct metabolic pathways were selected, each with two different doses, which included the mean and maximum concentrations measured in serum samples collected from mice with heart failure induced by transverse aortic constriction (*n* = 4; Table EV5). After 12 h of exposure, five metabolites including methylmalonic acid (biomarker of oxidative stress), succinic acid (involved in tricarboxylic acid cycle), ImP (from histidine metabolism), 2‐hydroxyisobutyric acid (from BCAAs metabolism), and 3‐hydroxybutyric acid (biomarker of lipid beta‐oxidation), as expected, induced higher levels of *NPPB* expression compared with the phosphate‐buffered saline (PBS) control; succinic acid and ImP even significantly elevated *NPPB* expression levels in H9c2 cells pretreated with hypoxia/reoxygenation (H/R), an important cause of CHF (Fig 3A). In contrast, four metabolites, including phenylacetylglutamine, had no impact on *NPPB* expression, which seemed contradictory to a recent study indicating upregulation of this gene upon phenylacetylglutamine exposure (Romano *et al*, 2023). The study by Romano *et al* used a dose of 100 μM, which was 1,000 times higher than the doses used in this study (0.11 and 0.16 μM). When the dose of this metabolite was increased to the same level, *NPPB* expression was indeed significantly upregulated (Fig EV5), confirming a crucial dose effect. Glyceric acid was expected to have a protective role against CHF but failed to suppress *NPPB* expression in the H/R pretreated cells and might even cause damage to normal cardiomyoblasts. Additional JC‐1 dye staining revealed that ImP disrupted cardiomyoblast functions, as indicated by significantly reduced mitochondrial membrane potential after exposure in both cell models (JC‐1 red/green ratios; Fig 3B and C).

### Mediation analyses with the clinical variables

**Figure 4:**
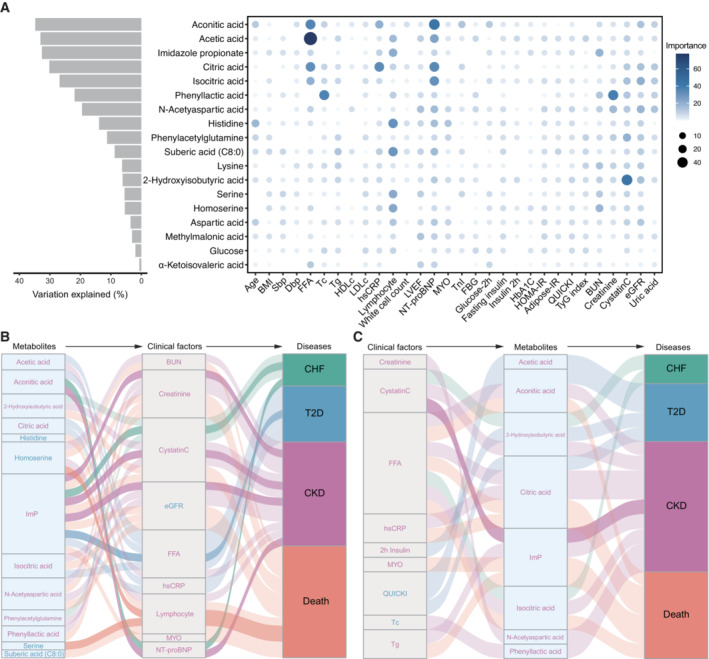
Mediation linkages among metabolites, clinical biomarkers, and diseases AThe association between altered metabolites and clinical variables. The bar plot shows the variance of serum metabolites that were explained by clinical factors based on the random forest model. The bubble plot depicts the importance of each clinical factor to the variation of the corresponding metabolite.B, CThe Sankey plot indicates the mediation relationships among metabolites, clinical factors, and diseases, with the clinical factors (B) and metabolites (C) as the underlying mediators, respectively. The ribbons were colored according to the associated diseases and text colored by the changes of corresponding metabolites or clinical factors, with red indicating increased and blue decreased serum levels in CHF patients. Source data are available online for this figure.

To address to what extent the altered metabolites associated with the clinical variables and if there were any associations between them, we performed machine‐learning regression and bidirectional mediation analyses, respectively, based on the 18 metabolites that showed the most robust associations with those three diseases (Fig 1; Table EV4). Our regression analyses, based on random forest, revealed that aconitic acids showed strongest associations with the clinical variables, especially NT‐proBNP and FFA (Fig 4A). ImP associated more with biomarkers of CKD than that of CHF and T2D. Our results, thus, indicated a pathogenic role of ImP not only for T2D but also for CHF and CKD and that shared etiologies such as those mediated by ImP do exist among those diseases. Further bidirectional mediation analysis implied that the link between aconitic acid to CKD and CHF might be mediated by NT‐proBNP, whereas the protective roles of serine and homoserine might be due to reduced inflammation (Fig 4B). In agreement, it was recently demonstrated that enhanced bacterial serine fermentation (in parallel with reduced serine circulation in the host) could boost the growth of pathogenic bacteria in the inflamed gut (Kitamoto *et al*, 2020). Our results also suggest that some microbially associated metabolites, including ImP, could in reverse be affected by clinical variables such as Cystatin C (Fig 4C).

### The utility in CHF rehospitalization/mortality prediction

**Figure 5:**
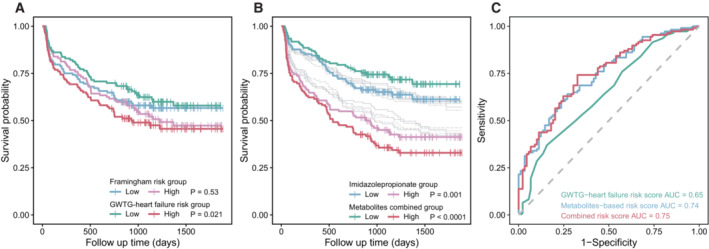
Performance of the metabolites in CHF prognosis AThe Kaplan–Meier survival curve based on the Framingham and GWTG‐HF risk scores. In total, 240 participants with clinical variables for Framingham and GWTG‐HF risk scores calculation were included in survival analysis (biological replicates). *P* values were calculated based on the log‐rank test.BThe Kaplan–Meier survival curve based on each microbially associated metabolite (gray colors except for imidazole propionic acid) and a combined metabolite risk score. In total, 244 participants who completed the follow‐up study were included in survival analysis (biological replicates). *P* values were calculated based on the log‐rank test.CThe time‐dependent receiver operating characteristic (ROC) curves for the performance of GWTG‐HF scores, metabolite‐based risk score, and the combination of both scores in CHF prognosis. The AUC values were determined by bootstrap resampling with 1,000 iterations. Source data are available online for this figure.

To examine if the identified metabolites could help predict a composite of CHF rehospitalization and mortality, we performed survival analyses and compared the performance of metabolites with that of the Framingham (D'Agostino Sr. *et al*, 2008) and GWTG‐HF (Get With the Guidelines‐Heart Failure) risk scores (Peterson *et al*, 2010). Results indicated that the GWTG‐HF risk scores, at least in our cohort, were more useful in predicting CHF rehospitalization/mortality than the Framingham risk scores (Fig 5A). We additionally showed that the risk scores derived from key microbially produced metabolites, such as ImP or a combination of all 18 metabolites, performed better than the GWTF‐HF risk score (Fig 5B). Further time‐dependent receiver operating characteristic (ROC) analysis confirmed that the metabolite risk score was superior than the GWTF‐HF risk score for CHF prognosis, and that combinations of both features barely improved the model performance (AUC from 0.74 to 0.75; Fig 5C).

## Discussion

We measured the serum levels of 151 microbially associated metabolites and examined their associations with CHF, T2D, and CKD in a Chinese heart failure cohort. Our findings indicate that many microbially associated metabolites, particularly those involved in amino acid metabolism, may contribute to the reprogramming of metabolic interconnectivities in CHF and its related comorbidities. In addition, cellular experiments provided evidence for causal links between several targeted metabolites and CHF relevant phenotypes. Further survival analyses demonstrated that a metabolite‐based risk score had better prognostic performance than existing Framingham or GWTG‐HF risk scores.

Amino acids, which can serve as metabolic or anaplerotic substrates, have been increasingly recognized as cardioprotective (Drake *et al*, 2012; Streng *et al*, 2022). Reduced circulating levels of amino acids, which associated negatively with New York Heart Association classes, have been reported in CHF patients (Aquilani *et al*, 2017). This is consistent with our findings that the circulating levels of most amino acids tend to decrease in CHF and its comorbidities, potentially due to inadequate dietary intake, reduced intestinal absorption, and/or gut microbial dysfunction. In agreement, inadequate protein intake, absorption, and negative nitrogen balance have been well documented (Aquilani *et al*, 2003; Arutyunov *et al*, 2008) and recently associated with higher CHF mortality (Streng *et al*, 2022), despite the fact that a detailed food frequency questionnaire was not available for our cohort. Our observation of reduced 2‐aminobutyric acid, which reflects dietary protein absorption to some extent, in the NGT + CHF subgroup, along with reduced serum albumin and protein malnutrition, suggests that reduced protein absorption and, consequently, increased bacterial fermentation in the gut may enhance the circulation of microbial metabolites downstream of amino acid metabolism. Our additional cellular experiments supported that six out of 10 of those microbial metabolites, including ImP, 2/alpha‐hydroxyisobutyric acid, and phenylacetylglutamine, appear to be causal to CHF relevant phenotypes in a dose‐dependent manner. Interestingly, both dietary protein and fat contents lost in the feces have been found to reflect mucosal barrier dysfunction (Arutyunov *et al*, 2008). Alternatively, we speculate that the order of T2D and CHF development may influence the reprogramming of associated metabolic signatures. It would be interesting to investigate whether serum levels of BCAAs increase if the onset of metabolic diseases precedes CHF and decrease if it occurs in the opposite order, as we observed in this study, as BCAAs have been repeatedly associated with various metabolic disorders, including T2D (White & Newgard, 2019).

Our findings also highlighted the importance of oxidative stress especially for those metabolites involved in the cysteine‐glutathione pathway to the pathogenesis and development of CHF and its comorbidities (van der Pol *et al*, 2019). 2‐aminobutyric acid and homoserine from cysteine biosynthesis, as well as hydrocinnamic acid derivatives, which possess antioxidant properties, were observed with reduced circulating levels in the disease groups, in line with their generally protective roles against these cardiometabolic disorders (Alam *et al*, 2016; Irino *et al*, 2016; Patel *et al*, 2020). Increased circulation of dimethylglycine and 2/alpha‐hydroxybutyric acid potentially reflects dysregulation of glutathione homeostasis and thus increased serum oxidative stress (McGregor *et al*, 2001). 2/alpha‐hydroxybutyric acid has also been suggested as an early biomarker of insulin resistance and glucose intolerance (Gall *et al*, 2010; Cobb *et al*, 2016). In addition, methylmalonic acid, an alternative biomarker for oxidative stress and mitochondrial dysfunction (Wang *et al*, 2020), was proven to be able to induce *NPPB* gene expression in cardiomyoblasts upon exposure in this study.

The failing heart also associated with reduced FFA utilization, which accounts for >70% myocardial carbon sources (Murashige *et al*, 2020). It has been shown that, for every standard deviation increase in serum FFA reflecting reduced heart consumption, there was a 12% higher incidence of CHF (Djousse *et al*, 2013). Similarly, we found that the serum levels of long‐chain fatty acid adrenic acid (C22:4) and short‐chain fatty acid acetic acid were both increased in most disease groups and were associated with higher hazard ratios for incidence of CHF rehospitalization and cardiovascular deaths, consistent with others (Lankinen *et al*, 2015; Delgado *et al*, 2017). However, no impact on *NPPB* gene expression levels was observed for either metabolites at physiological doses when exposed to cardiomyoblasts. In contrast, intermediates from incomplete FFA oxidation, such as the main ketone body 3‐hydroxybutyric acid and different carnitines, and those involved in the tricarboxylic acid cycle such as citric acid, isocitric acid, fumaric acid, aconitic acid and succinic acid, might represent more promising biomarkers for CHF development. For instance, elevated levels of citric acid and succinic acid have been associated with high risk of CHF (Bulló *et al*, 2021). Consistently, our cellular experiments demonstrated that both 3‐hydroxybutyric acid and succinic acid induced *NPPB* expression. Moreover, succinic acid could promote generation of reactive oxygen species (Mills *et al*, 2016), leading to myocardial cell deaths (Chouchani *et al*, 2014). However, it is essential to conduct further studies to determine whether increased circulation and utilization of those lipids represent compensatory mechanisms or true pathogenic factors for CHF and related comorbidities.

Our study has several limitations. First, the small sample size may have limited the statistical power of our analysis. However, we were able to assess CHF rehospitalization and mortality and validate our results in two other cohorts. Second, although we conducted bidirectional mediation and regression analyses, caution should be taken in interpreting the microbially associated metabolites as biomarkers or mediators of CHF, despite the fact that the causal roles for CHF relevant phenotypes have been shown *in vitro* for several representative metabolites. Third, some of the metabolite‐disease links identified in our study were confounded by medications as shown. Fourth, the lack of detailed nutritional data prevented us from determining whether the altered serum molecules were due to diet, or the diseases, or both.

In summary, our study identified several potential microbial metabolites and pathways that could be utilized for monitoring and predicting CHF multimorbidity. The strengthened link between the gut–heart axis and nutrient metabolism supported dietary modulation of the gut microbiota as a promising therapy for CHF. Overall, our findings provide a valuable resource for advancing our understanding of the human gut microbiota in the precise management of CHF and its comorbidities.

## Materials and Methods

### Description of the study cohort

The REM‐HF study is a prospective cohort study of 5,344 individuals recruited from China between 2016 and 2019 (registered at ClinicalTrials.gov with identifier: NCT02998788). In total, 260 individuals (29.23% female) aged 24–87 years old were selected for our targeted metabolomics analysis according to the defined exclusion criteria in Fig EV1. Individuals were followed up for CHF rehospitalization or cardiovascular deaths until August 31, 2021. A total of 244 individuals completed the follow‐up study with 89 CHF rehospitalization and 24 cardiovascular deaths reported. All participants gave their informed written consent. This study was approved by the Institutional Review Board of Ruijin Hospital, Shanghai Jiao Tong University and conformed to the principles set out in the WMA Declaration of Helsinki and the Department of Health and Human Services Belmont Report.

### Clinical, biochemical, and echocardiographic assessments

Anthropometric features were collected from electronic medical records. The blood samples were obtained after at least 8 h fasting and 20 min rest in supine position. Most clinical biomarkers were measured using a chemiluminescent method (AU5800; Beckman, Carlsbad, CA). Transthoracic echocardiography was conducted using a commercially available system (Vivid‐I, GE Healthcare, Milwaukee, WI). Two‐dimensional pulsed‐Doppler imaging was performed from standard parasternal and apical transducer positions with 2D frame rates of 60–100 frames/s. LVEF (left ventricular ejection fraction) was calculated using the modified Simpson's biplane technique (EchoPac, version 7; GE Healthcare).

### Definitions of prediabetes, heart failure and chronic kidney diseases

Prediabetes was defined as fasting blood glucose in the range of 6.1–6.9 mmol/l and/or 2‐h blood glucose 8.9–12.1 mmol/l and/or HbA1c (glycated hemoglobin) 5.7–6.4%. T2D was defined as fasting blood glucose ≥ 7.0 mmol/l and/or 2‐h blood glucose ≥ 12.2 mmol/l and/or HbA1c ≥ 6.5%. Chronic heart failure was defined as LVEF < 40% or left atrial dilation concomitant with NT‐proBNP > 400 ng/l. Chronic kidney disease was defined as eGFR (estimated glomerular filtration rate) < 60 ml/min/1.72 m^2^ and/or the presence of albuminuria.

### Definition of microbially associated metabolites

Metabolites were considered as microbially associated if they were (i) annotated as microbially produced in the HMDB database (Human Metabolome Database, version 5.0; Wishart *et al*, 2022); (ii) detected with four times higher for their concentrations in gut bacteria culturing medium than in controls or in conventionally‐raised than in germ‐free mice (Han *et al*, 2021); or (iii) significantly associated with the gut microbiome as reported recently (Bar *et al*, 2020). Altogether, 588 metabolites were defined as microbially associated; of these, 151 metabolites could be targetedly quantified using the Q300 platform based on a UPLC‐MS/MS system.

### Targeted metabolomics profiling

The blood samples were centrifuged at 1,000 *g* for 10 min to separate the serum within 12 h after collection, and the aliquoted serum samples were stored at −80°C for further analysis. Absolute quantification of the microbially associated metabolites was conducted based on the Q300 platform (Metabo‐Profile Biotechnology, Shanghai, China) as previously described (Xie *et al*, 2021). In brief, 25 μl of serum samples in a 96‐well plate was mixed with 120 μl methanol and then vortexed vigorously for 5 min and centrifuged at 4,000 *g* for 30 min. In total, 30 μl of supernatant and 20 μl of freshly prepared derivative reagents were added to a clean 96‐well plate for further derivatization. The samples were further diluted using 330 μl of ice‐cod 50% methanol solution and centrifuged. In total, 135 μl of supernatant was transferred to a new 96‐well plate with 10 μl internal standards in each well. Measurement was performed using a UPLC‐MS/MS system (ACQUITY UPLC‐Xevo TQ‐S, Waters Corp., Milford, MA, USA) with the following settings: BEHC18 1.7 μM VanGuard pre‐column and analytical column; water with 0.1% formic acid for mobile phase A and acetonitrile/IPA for mobile phase B; flow rate at 0.4 ml/min; and capillary (Kv) = 1.5 (ESI^+^), 2.0 (ESI^−^). The raw data files generated by UPLC‐MS/MS were processed using the MassLynx software (v4.1) to perform peak integration, calibration, and quantification for each metabolite.

### Identification of metabolites associated with cardiometabolic stress in BPRHS cohort

The cardiometabolic stress (CM) index in BPRHS cohort was calculated as previously described (Murthy *et al*, 2020). This index is based on nine components associated with metabolic, inflammatory, and neurohormonal states, with a score of 0, 1, or 2 assigned to each component. The scores are then summed up to obtain a score ranging from 0 to 11 defined as the CM index. To identify metabolites associated with this index, we divided participants in the BPRHS cohort into the following three groups: a low‐risk group (group1, 0 ≤ CM index ≤ 3, *n* = 212), an intermediate‐risk group (group2, 4 ≤ CM index ≤ 5, *n* = 301), and a high‐risk group (group3, 6 ≤ CM index, *n* = 227). We calculated the fold‐change of each metabolite by comparing its abundance in group 2 or group 3 to group 1, respectively.

### Transverse aortic constriction (TAC) mice model

C57/BL6 mice were purchased from Model Animal Research Center of Nanjing University and housed in the Experimental Medical Research Center of Ruijin Hospital with a standard 12 h light–dark cycle and free access to food and water. Minimally invasive TAC was performed on 8‐week‐old male mice (*n* = 4). Prior to the procedure, the mice were anesthetized with an intraperitoneal injection of 2.5% pentobarbital and connected to a mechanical ventilation device to ensure their breathing. The procedure began with a horizontal skin incision, approximately 0.5–1.0 cm in length, made at the level of the suprasternal notch. The transverse aortic arch was then exposed, and a 5–0 silk suture was carefully looped around the aorta and tied around a 27‐gauge needle placed next to the aortic arch. After the ligation, the needle was removed, and the skin was closed. The mice were then allowed to recover on a warming pad until fully awake. Blood from the mice eyeballs was collected 6 weeks after the surgery and centrifuged at 4°C, 3,000 rpm for 20 min. After centrifugation, the supernatant serum was stored at −80°C until further metabolomics analysis. All animal experimental protocols were approved by the Committee on the Ethics of Animal Experiments of Ruijin Hospital.

### Cell‐metabolite co‐culturing and quantitative real‐time PCR analysis

The H9c2 cell line was obtained from the National Collection of Authenticated Cell Cultures in China (identifier:CSTR:19375.09.3101RATGNR5) and routinely tested for mycoplasma contamination. Cells was cultured in Dulbecco's Modified Eagle Medium (DMEM) medium supplemented with 10% fetal bovine serum, 100 U/ml penicillin, and 100 μg/ml streptomycin at 37°C in a humidified atmosphere containing 5% CO_2_. The cells were fed every 2 days and subcultured when they reached 70–80% confluence to prevent any changes in cellular phenotype. All metabolites were dissolved in phosphate‐buffered saline (PBS) for subsequent use, with the exception of adrenic acid (C22:4), which was dissolved in ethanol first before being diluted in PBS. Three groups of H9c2 cells, each with approximately 80% confluency, were exposed to either different doses of each metabolite or an equal volume of PBS for a duration of 12 h. To establish a hypoxic condition, the cells were transferred to a glucose‐free medium within an airtight Plexiglas hypoxic chamber for 6 h, while being exposed to the metabolite or PBS. This was followed by a reoxygenation period of 6 h.

Total RNA was isolated from H9c2 cells using TRIzol reagent (Cat#9109, Takara, Japan) following the manufacturer's instructions. The quality and purity of the RNA were assessed using a NanoDrop‐2000 spectrophotometer. Real‐time PCR was performed on an Applied Biosystems 7500 instrument using SYBR Green PCR Master Mix (Cat#RR036A, Takara, Japan). The relative mRNA levels were calculated using the $2^{-\Delta \Delta C_{t}}$ method and normalized to β‐actin. The following primers were used for PCR: rat β‐actin, F 5′‐TTGTGATGGACTCCGGAGAC‐3′ and R 5′‐TGATGTCACGCACGATTTCC‐3′; rat BNP‐encoding gene *NPPB*, F 5′‐AGACAGCTCTCAAAGGACCA‐3′, and R 5′‐CCGGTCTATCTTCTGCCCAA‐3′.

### Measurement of mitochondrial membrane potential

Changes in mitochondrial membrane potential were assessed by JC‐1 staining with fluorescence microscopy. In brief, H9c2 cells were incubated with JC‐1 at 37°C for 20 min and then washed twice with JC‐1 staining buffer (1×). The cells were visualized on Petri dishes using a fluorescence microscope (IX83, Olympus). The red emission of JC‐1 dye indicates potential‐related aggregation in the mitochondria, whereas the green fluorescence represents the monomeric form of JC‐1 that appears in the cytoplasm after depolarization of the mitochondrial membrane. We analyzed the ratio of aggregate to monomer using ImageJ software. The cells were treated with imidazole propionic acid (0.1 μM for 24 h) or subjected to 6 h of hypoxia followed by 6 h of reoxygenation (H/R).

### Statistical analyses

All statistical analyses were conducted in the R environment (version 4.0.4). All samples were included in the analysis, with the exception of individuals who did not complete the follow‐up study. We did not use cell randomization or blinding of investigators. The nonparametric Wilcox rank‐sum test was used to identify metabolites that differed significantly between groups. For categorical variables, the Chi‐squared test was used. Drug‐deconfounding analysis for the metabolite‐disease links was performed using the metadeconfoundR package (v0.1.8; Forslund *et al*, 2021). The multivariable Cox proportional hazards model (survival v3.2.13; Therneau, 2019) and random forest model (randomForest v4.6.14; Liaw & Wiener, 2002) with sex adjustment (ntree = 3,000, mtry = 2–30) were utilized. Bidirectional mediation analysis (mediation v4.5.0; Tingley *et al*, 2014) was performed with age and sex adjustment. Kaplan–Meier survival analysis (survminer v0.4.9; Kassambara *et al*, 2021) and time‐dependent ROC curves (riskRegression v2021.10.10; Gerds & Kattan, 2021) were conducted with bootstrap resampling (*n* = 1,000). The log‐rank test was applied to compare survival curves between groups. Raw *P* values were adjusted by the Benjamini–Hochberg method (Benjamini & Hochberg, 1995). Adjusted *P* values < 0.1 and raw *P* values < 0.05 were considered statistically significant.

## Author contributions

**Sha Hua:** Data curation; formal analysis; funding acquisition; methodology; writing – original draft; writing – review and editing. **Bomin Lv:** Data curation; software; formal analysis; funding acquisition; visualization; methodology; writing – original draft; writing – review and editing. **Zeping Qiu:** Data curation; formal analysis; validation; methodology; writing – original draft; writing – review and editing. **Zhuojin Li:** Data curation; formal analysis; validation; methodology; writing – review and editing. **Zhiyan Wang:** Data curation; project administration; writing – review and editing. **Yanjia Chen:** Data curation; project administration; writing – review and editing. **Yanxin Han:** Data curation; methodology; project administration; writing – review and editing. **Katherine L Tucker:** Resources; data curation; writing – review and editing. **Hao Wu:** Conceptualization; resources; data curation; software; formal analysis; supervision; visualization; methodology; writing – original draft; project administration; writing – review and editing. **Wei Jin:** Conceptualization; data curation; formal analysis; supervision; funding acquisition; validation; writing – original draft; project administration; writing – review and editing.

## Disclosure and competing interests statement

The authors declare that they have no conflict of interest.

## For more information

https://omicsdata.org/Apps/REM‐HF/.

## Supporting information



Expanded View Figures PDF

Table EV1

Table EV2

Table EV3

Table EV4

Table EV5

PDF+

Source Data for Figure 1

Source Data for Figure 2

Source Data for Figure 3

Source Data for Figure 4

Source Data for Figure 5

## Data Availability

The datasets produced in this study are available in the following databases: raw metabolomics data: OMICSDATA.org OD10001 (https://omicsdata.org/Apps/REM‐HF/Download).
