# Supplementary material for: Microbial metabolites in chronic heart failure and its common comorbidities
Source: EMBO Mol Med. 2023 May 8;15(6):e16928. doi: 10.15252/emmm.202216928 (PMC10245034; doi:10.15252/emmm.202216928)
Supplement: Supplementary file 1 — Expanded View Figures PDF [file EMMM-15-e16928-s007.pdf]

Expanded View Figures

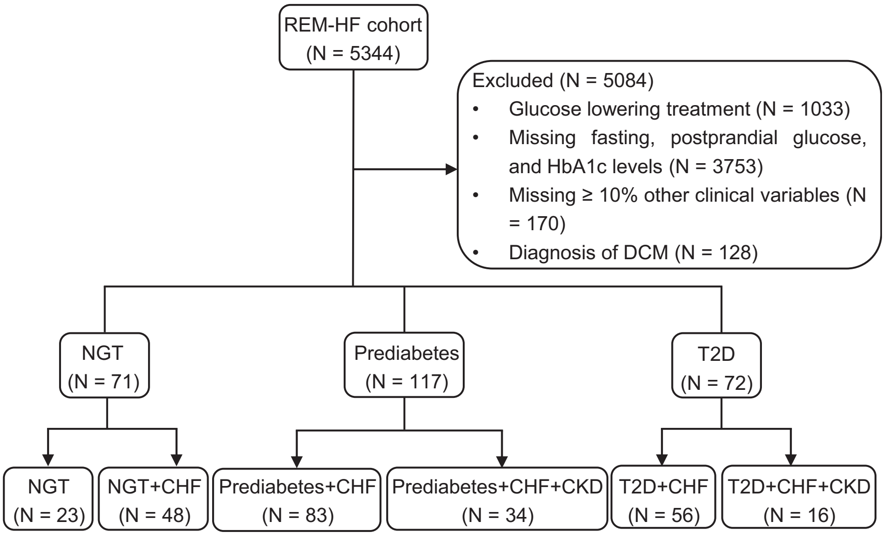

Figure EV1. CONSORT diagram for REM-HF cohort.

CHF, chronic heart failure; CKD, chronic kidney disease; DCM, Dilated cardiomyopathy; NGT, normal glucose tolerance; T2D, type 2 diabetes.

Figure EV2. Absolute plasma levels of all 151 microbially associated metabolites in REM-HF cohort.

The central band in each box represents the median, the top, and bottom of the box the 25th and 75th percentiles, and the whiskers 1.5 times the interquartile range ($n = 260$; biological replicates).

▶

© 2023 The Authors

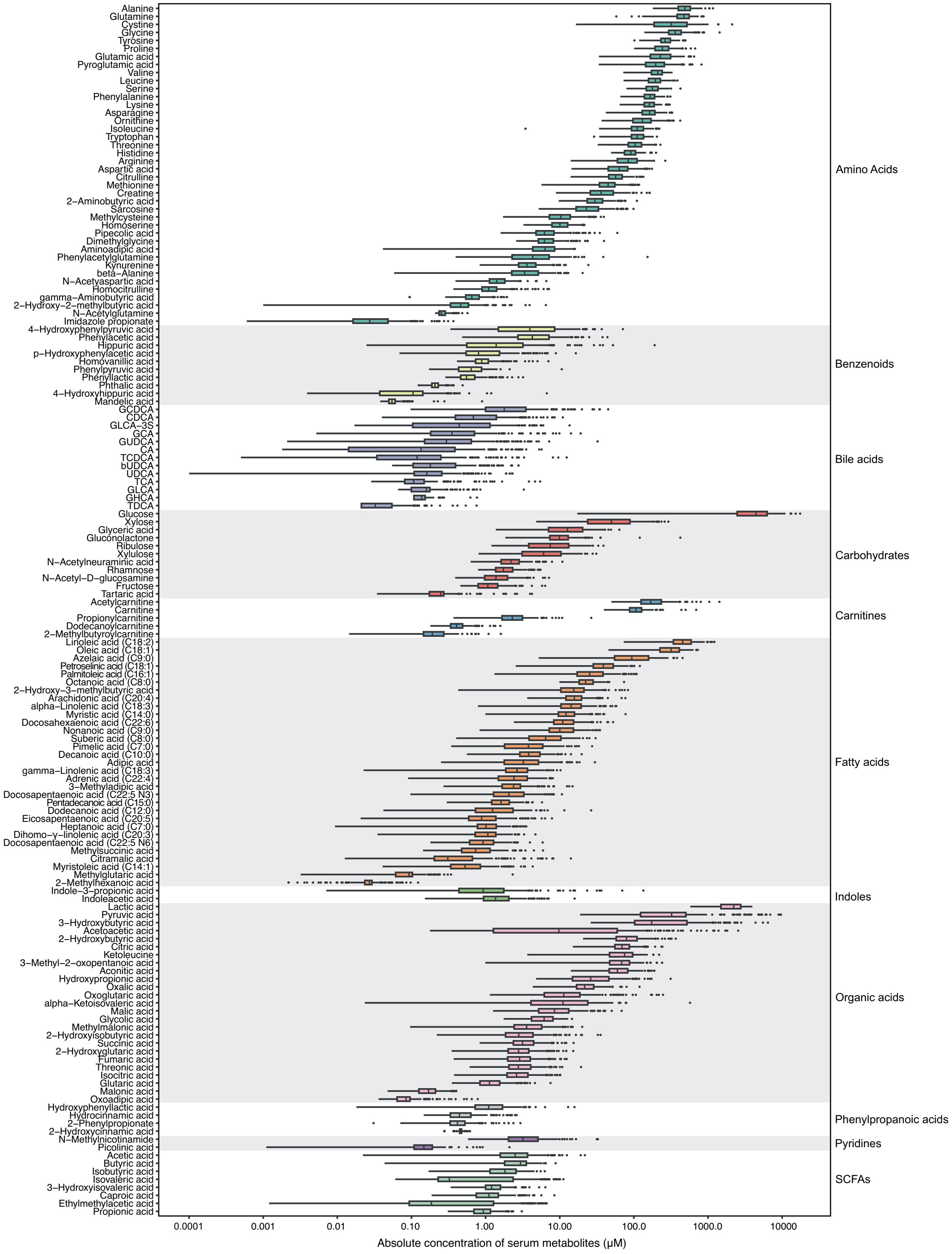

Figure EV2.

 © 2023 The Authors

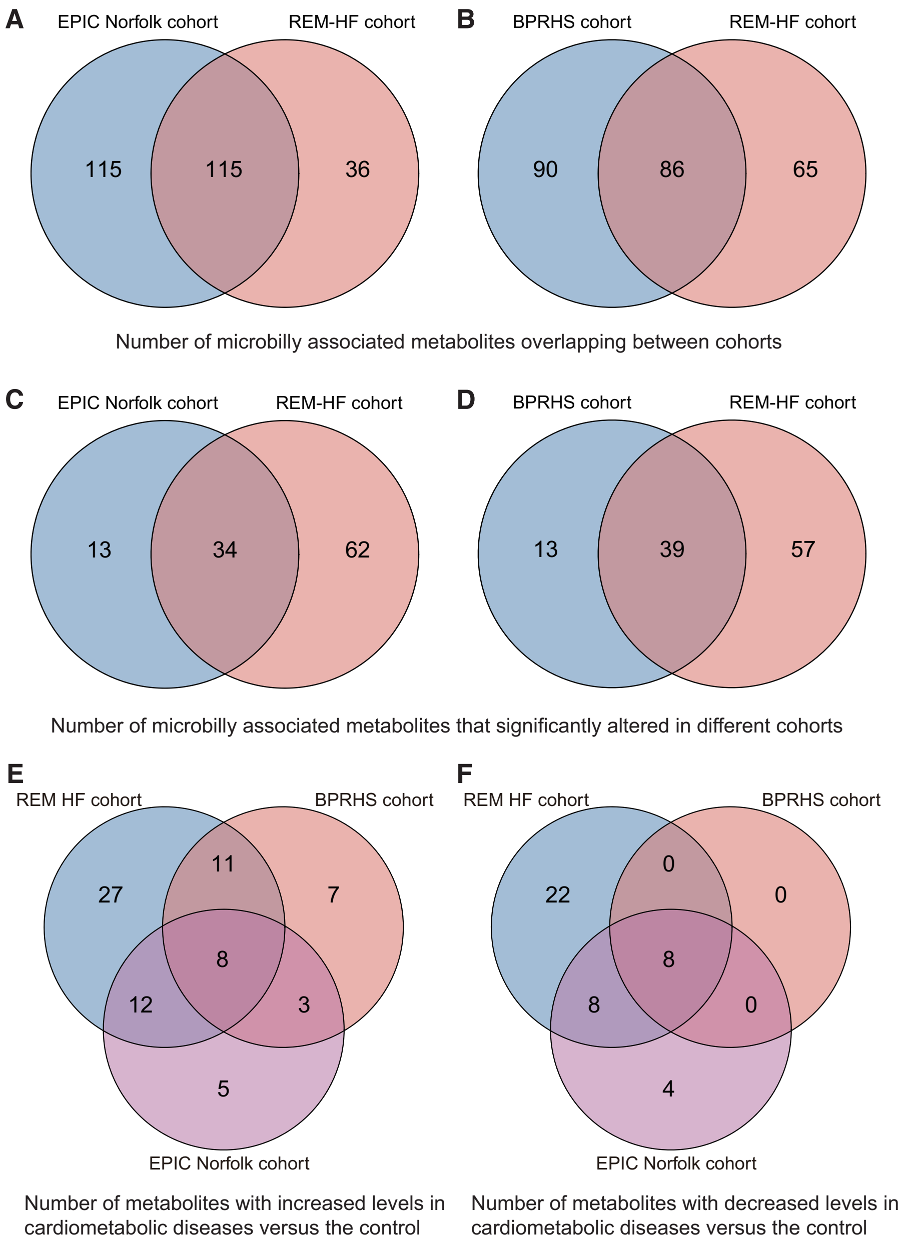

Figure EV3. Venn plots depicting numbers of shared metabolites across cohorts.

A–F Numbers of metabolites (A) and significantly changed metabolites (C) that were overlapping between REM-HF and EPIC-Norfolk cohorts and similarly for those (B, D) between REM-HF and BPRHS cohorts. Numbers of increased (E) and decreased (F) metabolites across all three cohorts, respectively.

© 2023 The Authors

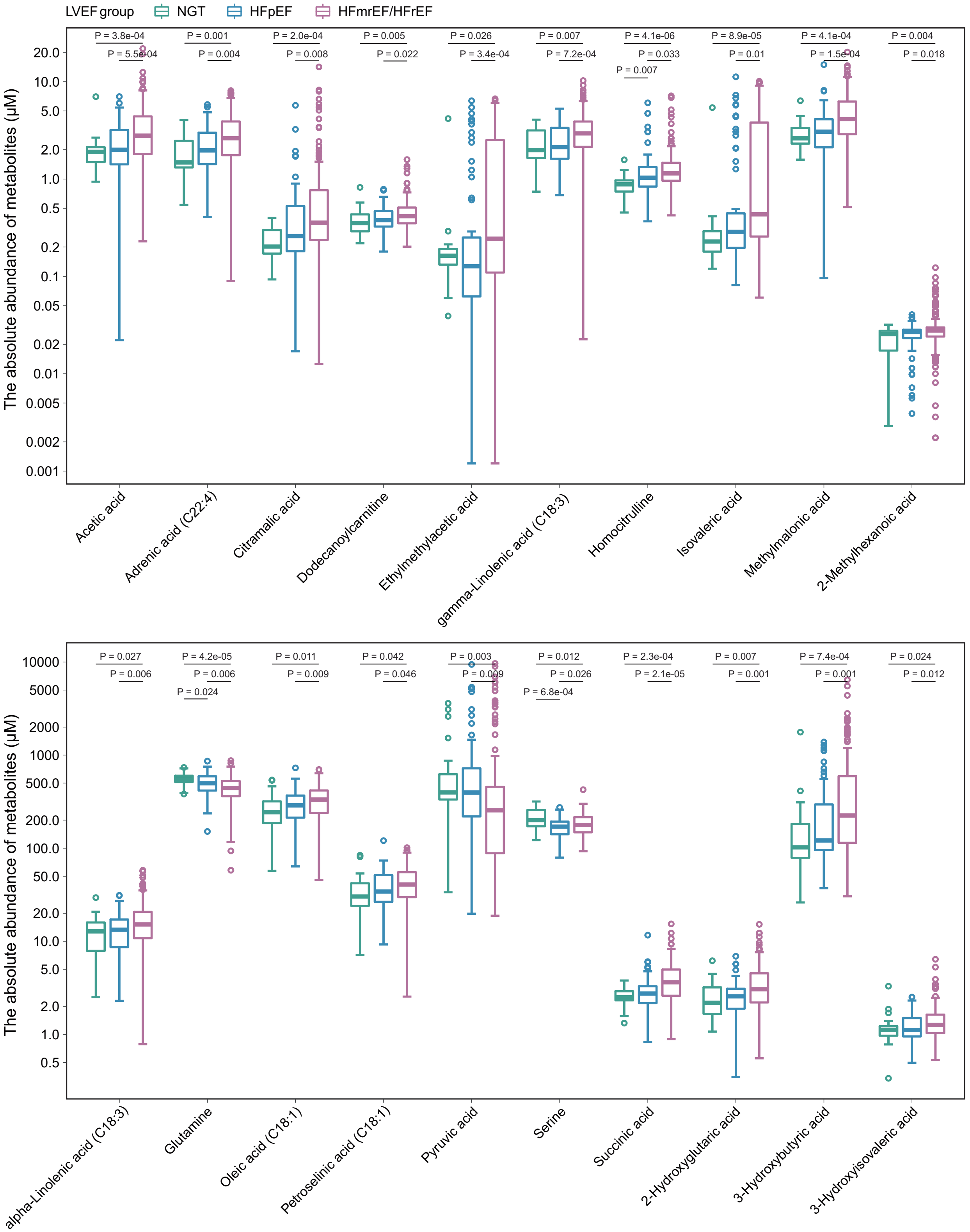

Figure EV4. Metabolites that differed between different LVEF groups in REM-HF cohort.

The central band in each box represents the median, the top, and bottom of the box the 25th and 75th percentiles, and the whiskers 1.5 times the interquartile range. NGT: n = 23, HFpEF: LVEF ≥ 50, $n = 66$; HFmEF: 40 < LVEF < 50, $n = 68$; HFrEF: LVEF ≤ 40, $n = 103$ (biologcial replicates).

© 2023 The Authors

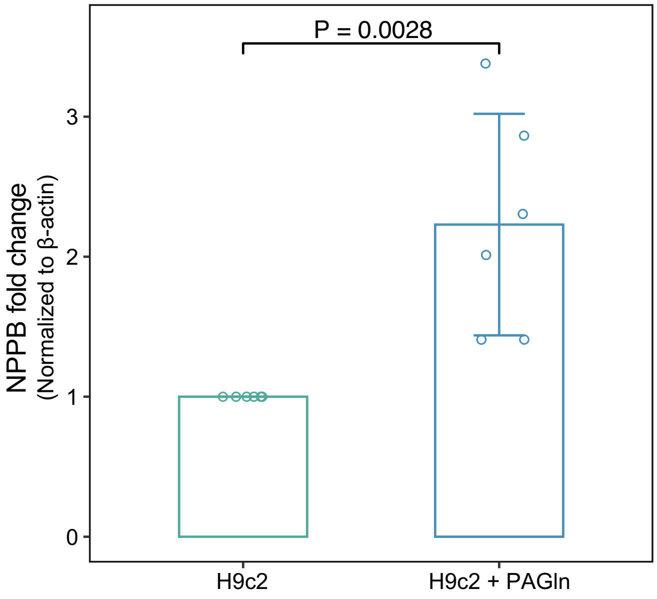

Figure EV5. The relative expression of *NPPB* upon phenylacetylglutamine exposure.

The relative expression of *NPPB* was detected by RT-PCR after 6-h exposure to 100 μM phenylacetylglutamine ($n = 6$, biological replicates). The data are shown as mean ± s.d. Statistical analysis was performed by the Wilcoxon rank-sum test.

© 2023 The Authors
