# Supplementary figures and images for: Microbial metabolites in chronic heart failure and its common comorbidities
Source: EMBO Mol Med. 2023 May 8;15(6):e16928. doi: 10.15252/emmm.202216928 (PMC10245034; doi:10.15252/emmm.202216928)

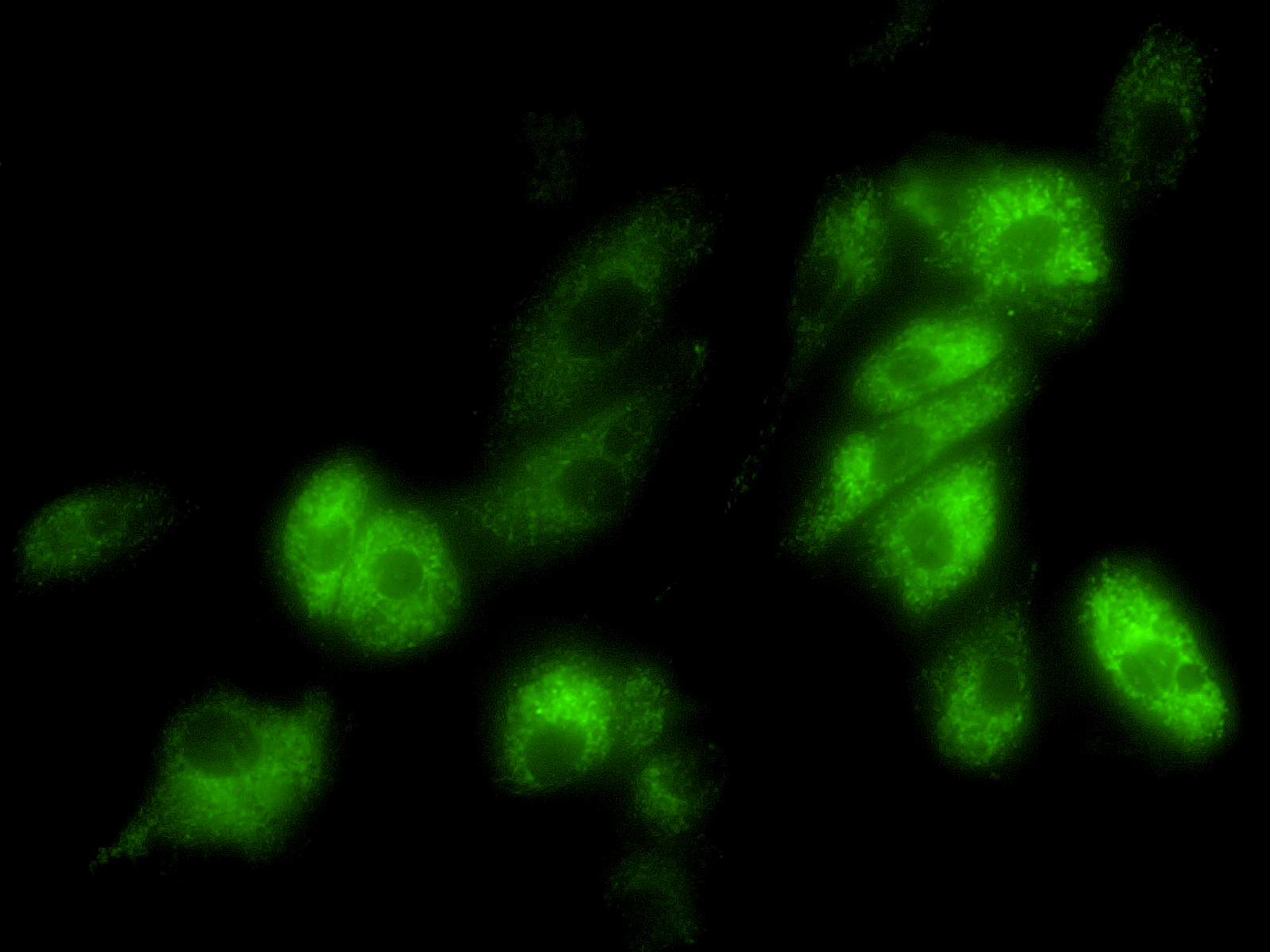

Supplement: Supplementary file 10 — Source Data for Figure 3 [file EMMM-15-e16928-s003.zip › Figure3/3B/H9c2+H:R_Monomer.tif]

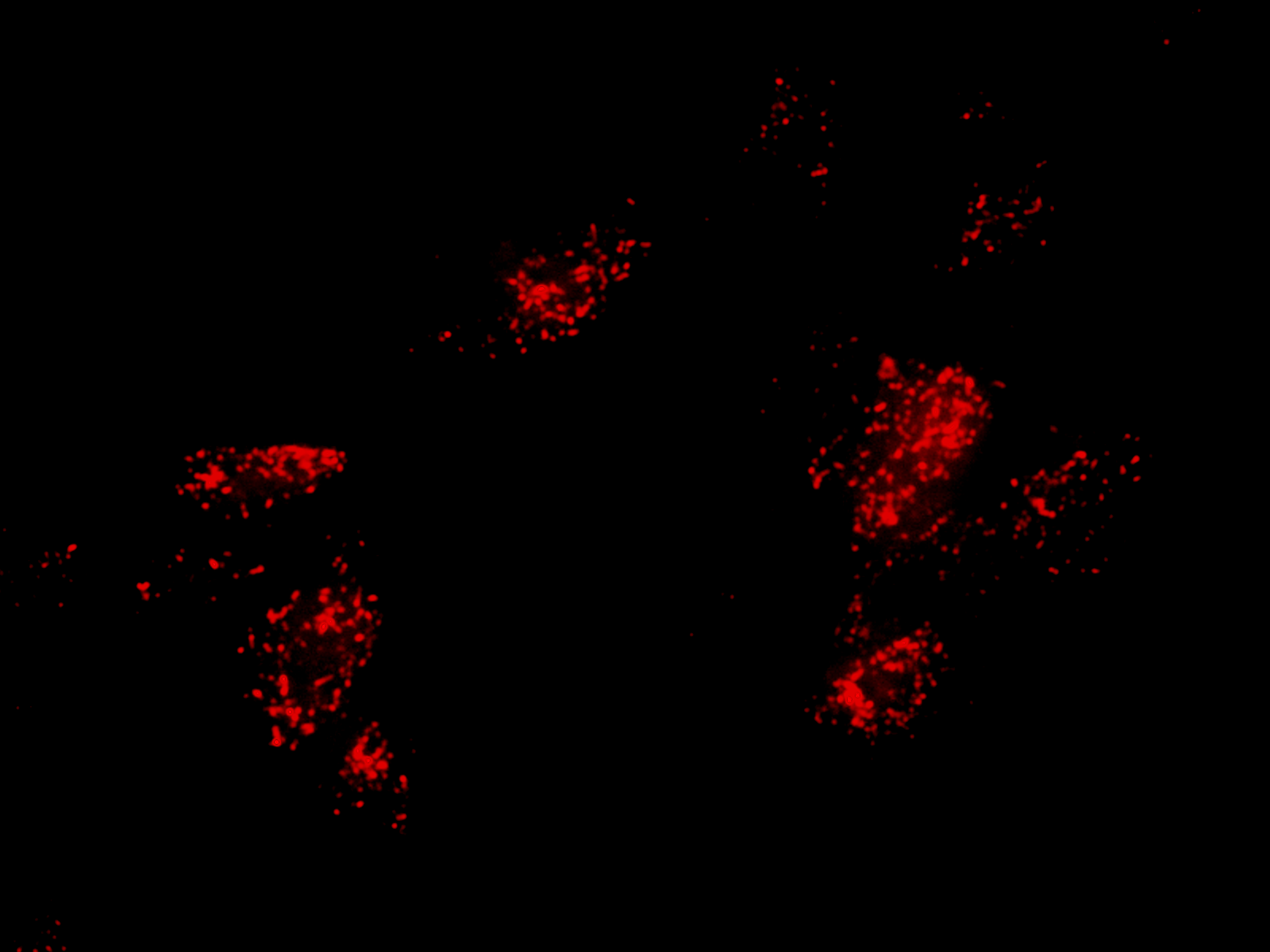

Supplement: Supplementary file 10 — Source Data for Figure 3 [file EMMM-15-e16928-s003.zip › Figure3/3B/H9c2+H:R+ImP_Aggregate.tif]

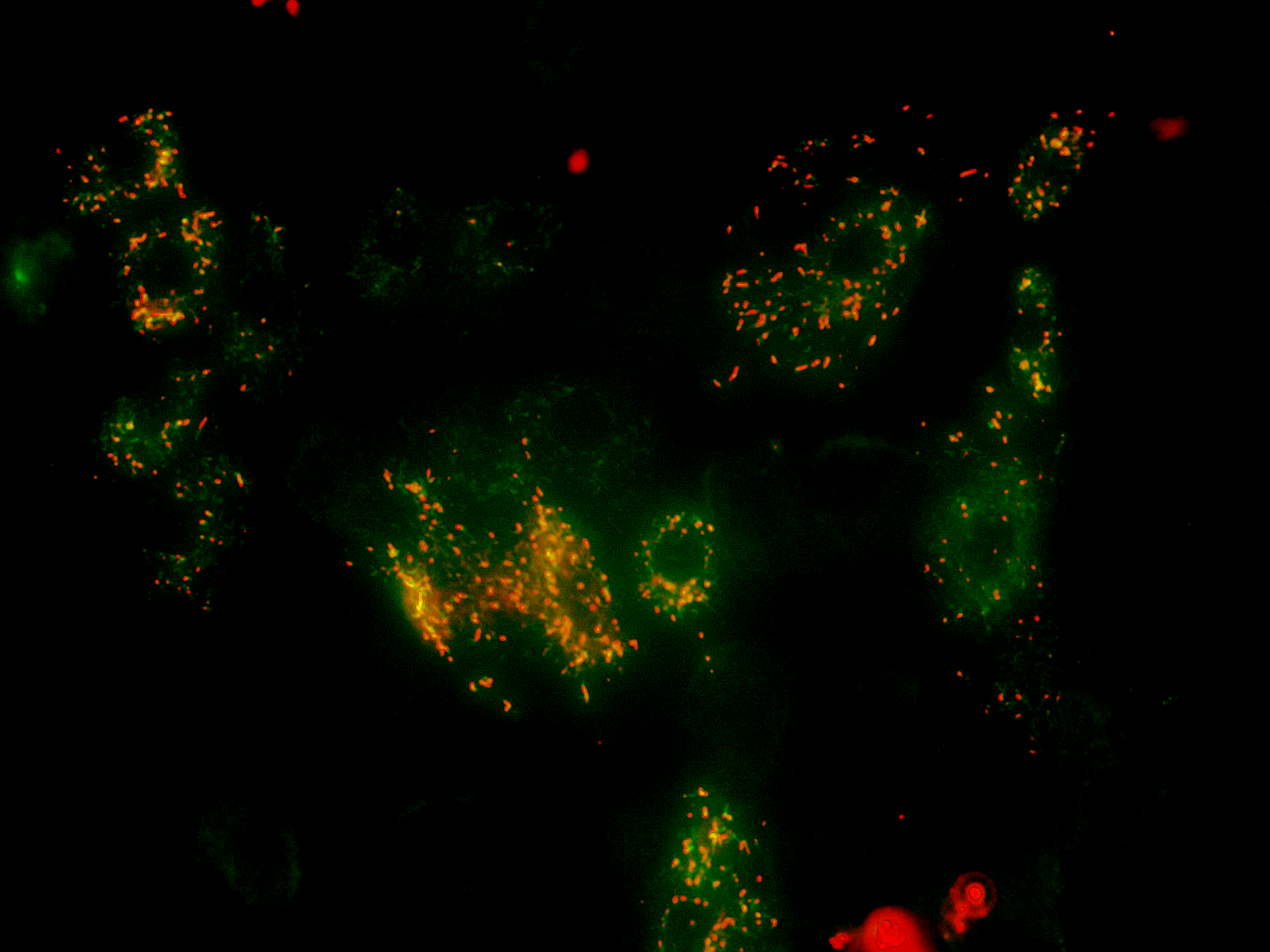

Supplement: Supplementary file 10 — Source Data for Figure 3 [file EMMM-15-e16928-s003.zip › Figure3/3B/H9c2+ImP_Merged.tif]

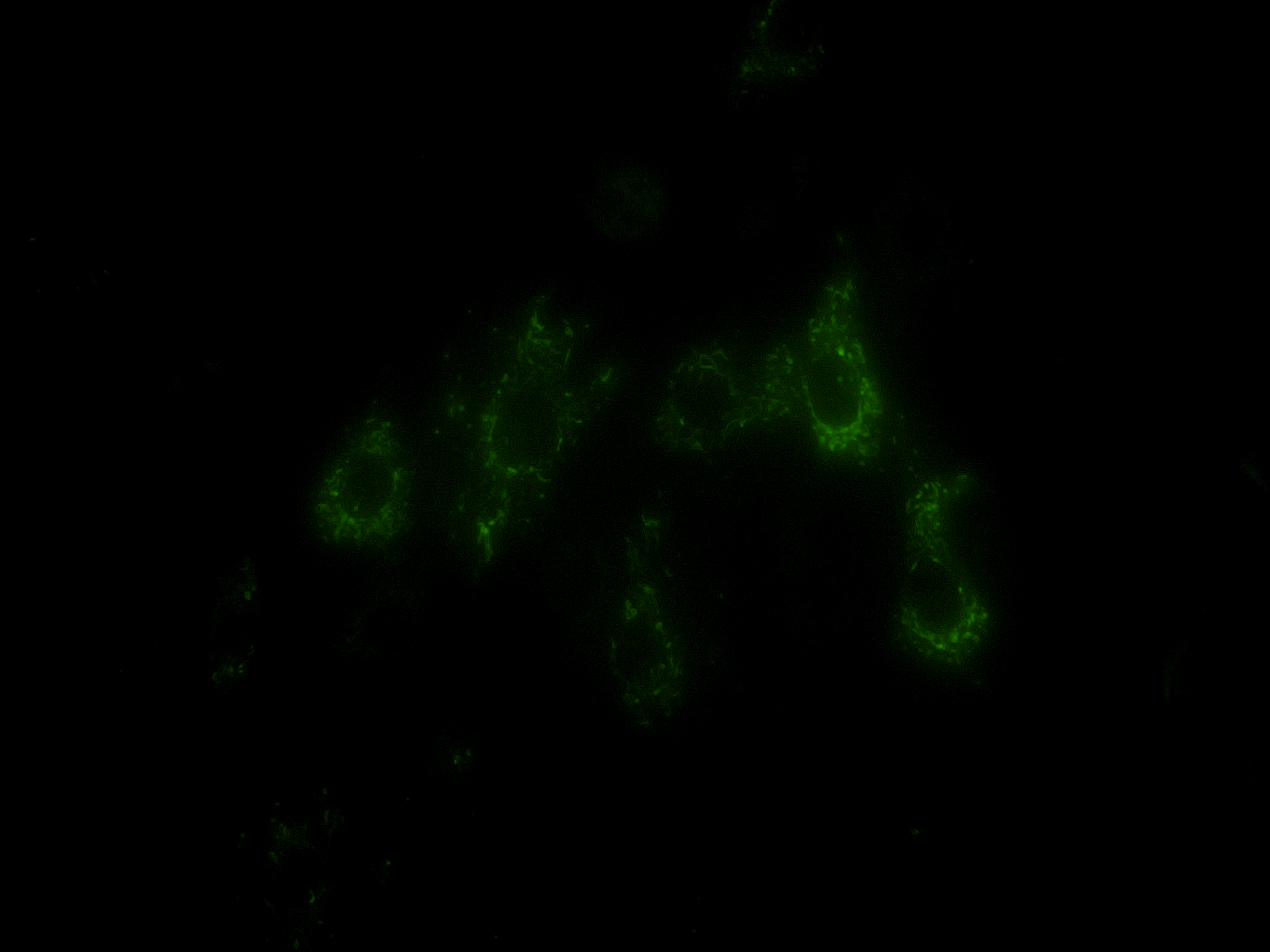

Supplement: Supplementary file 10 — Source Data for Figure 3 [file EMMM-15-e16928-s003.zip › Figure3/3B/H9c2_Monomer.tif]

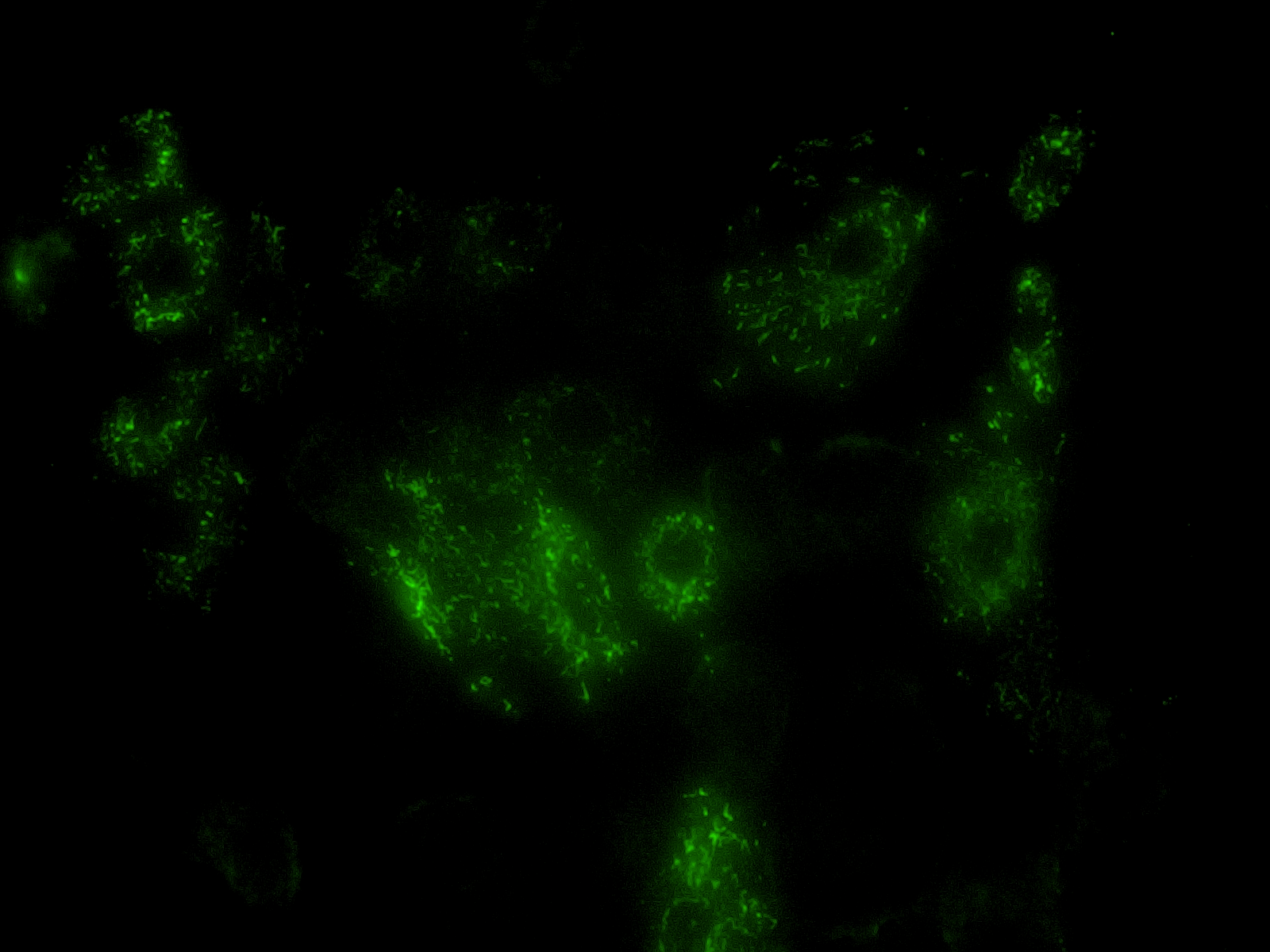

Supplement: Supplementary file 10 — Source Data for Figure 3 [file EMMM-15-e16928-s003.zip › Figure3/3B/H9c2+ImP_Monomer.tif]

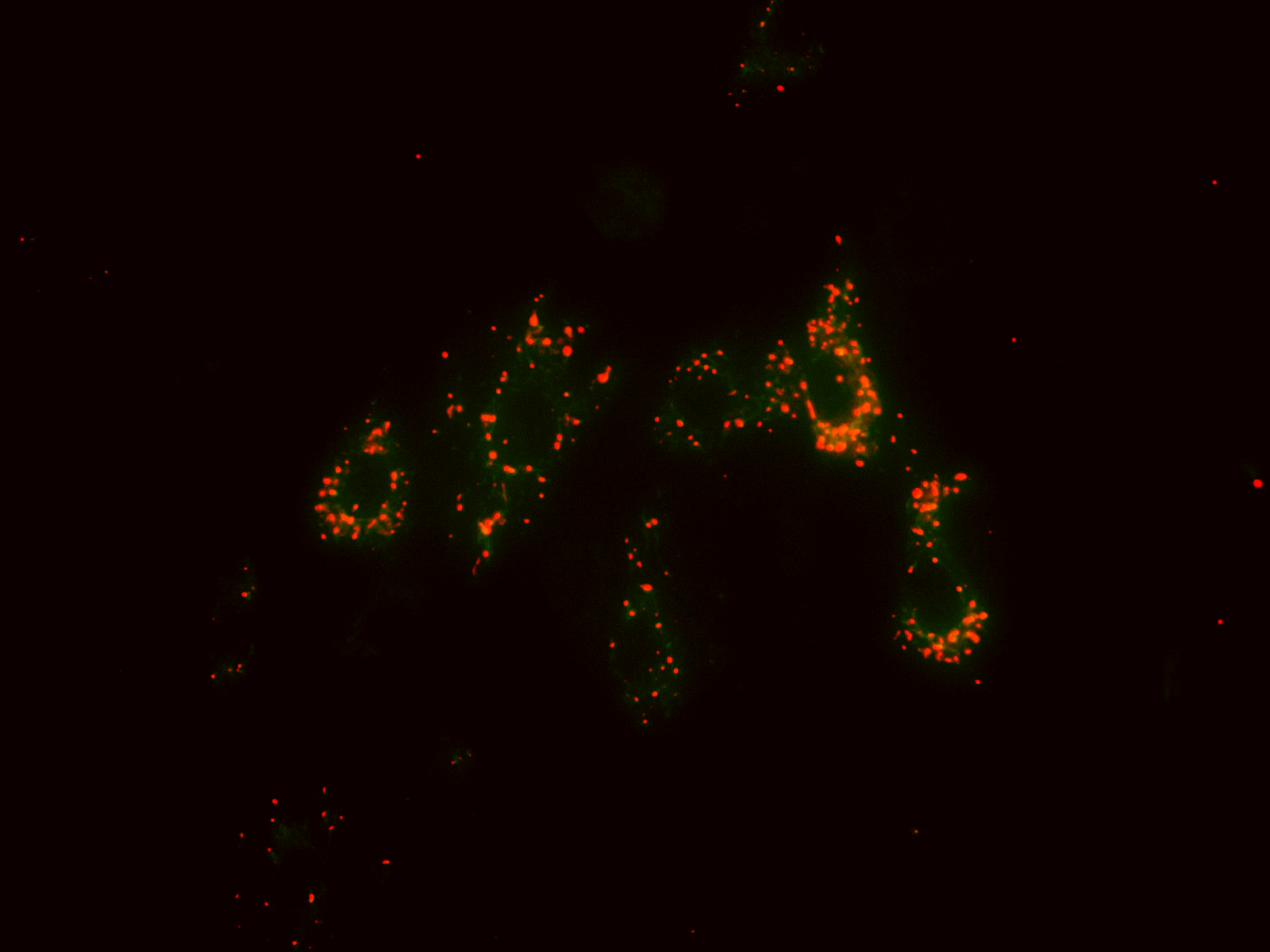

Supplement: Supplementary file 10 — Source Data for Figure 3 [file EMMM-15-e16928-s003.zip › Figure3/3B/H9c2_Merged.tif]

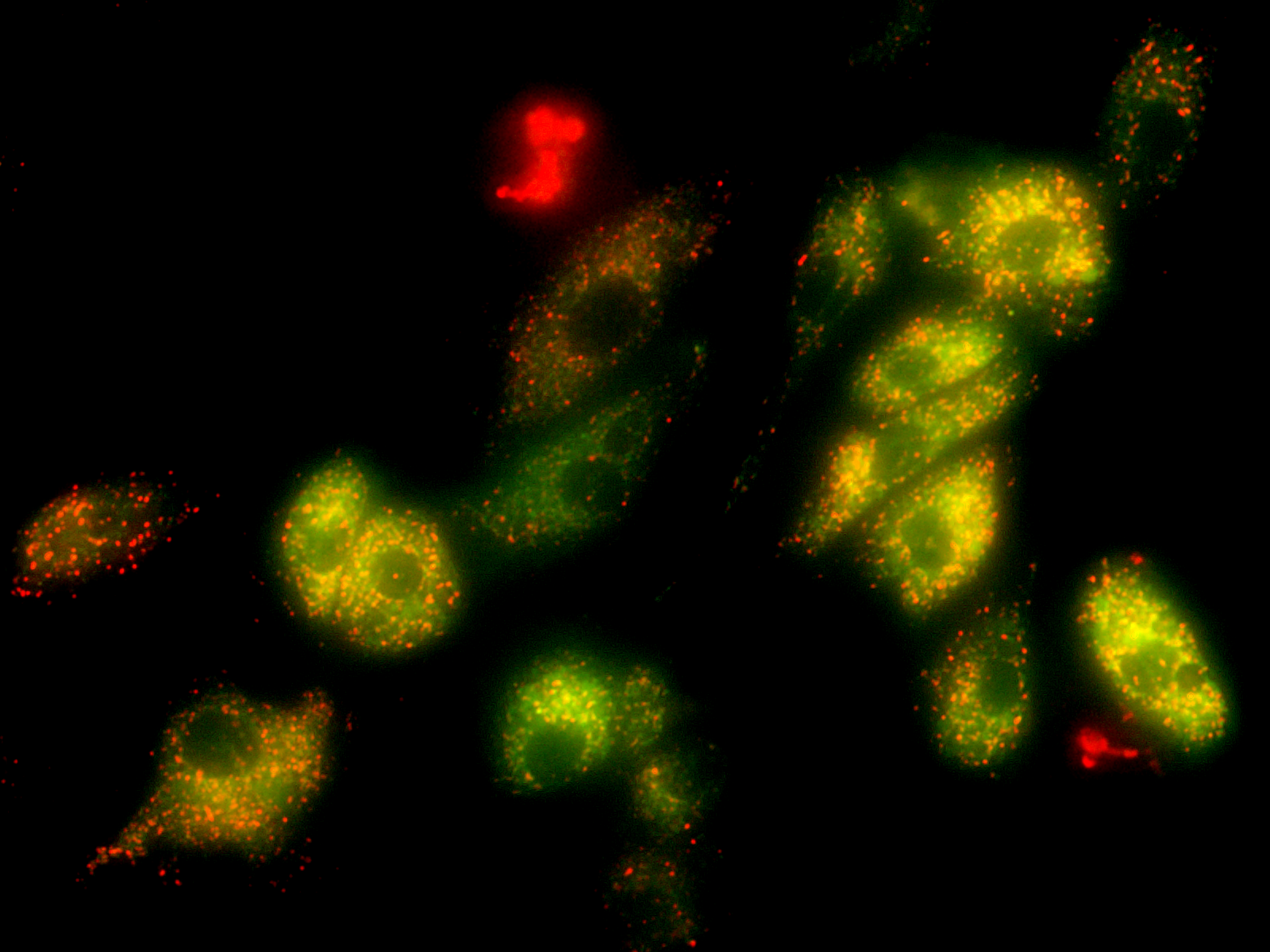

Supplement: Supplementary file 10 — Source Data for Figure 3 [file EMMM-15-e16928-s003.zip › Figure3/3B/H9c2+H:R_Merged.tif]

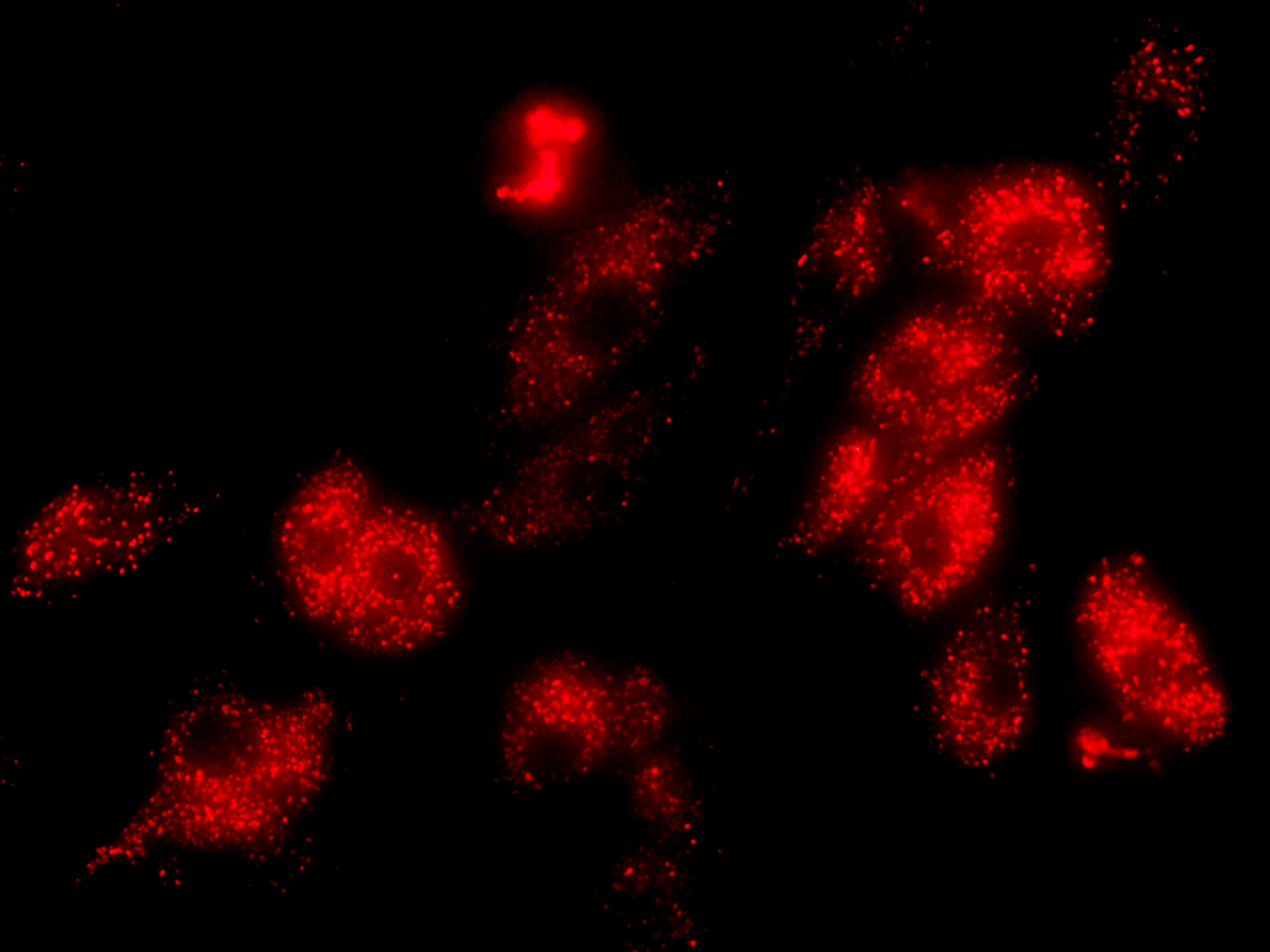

Supplement: Supplementary file 10 — Source Data for Figure 3 [file EMMM-15-e16928-s003.zip › Figure3/3B/H9c2+H:R_Aggregate.tif]

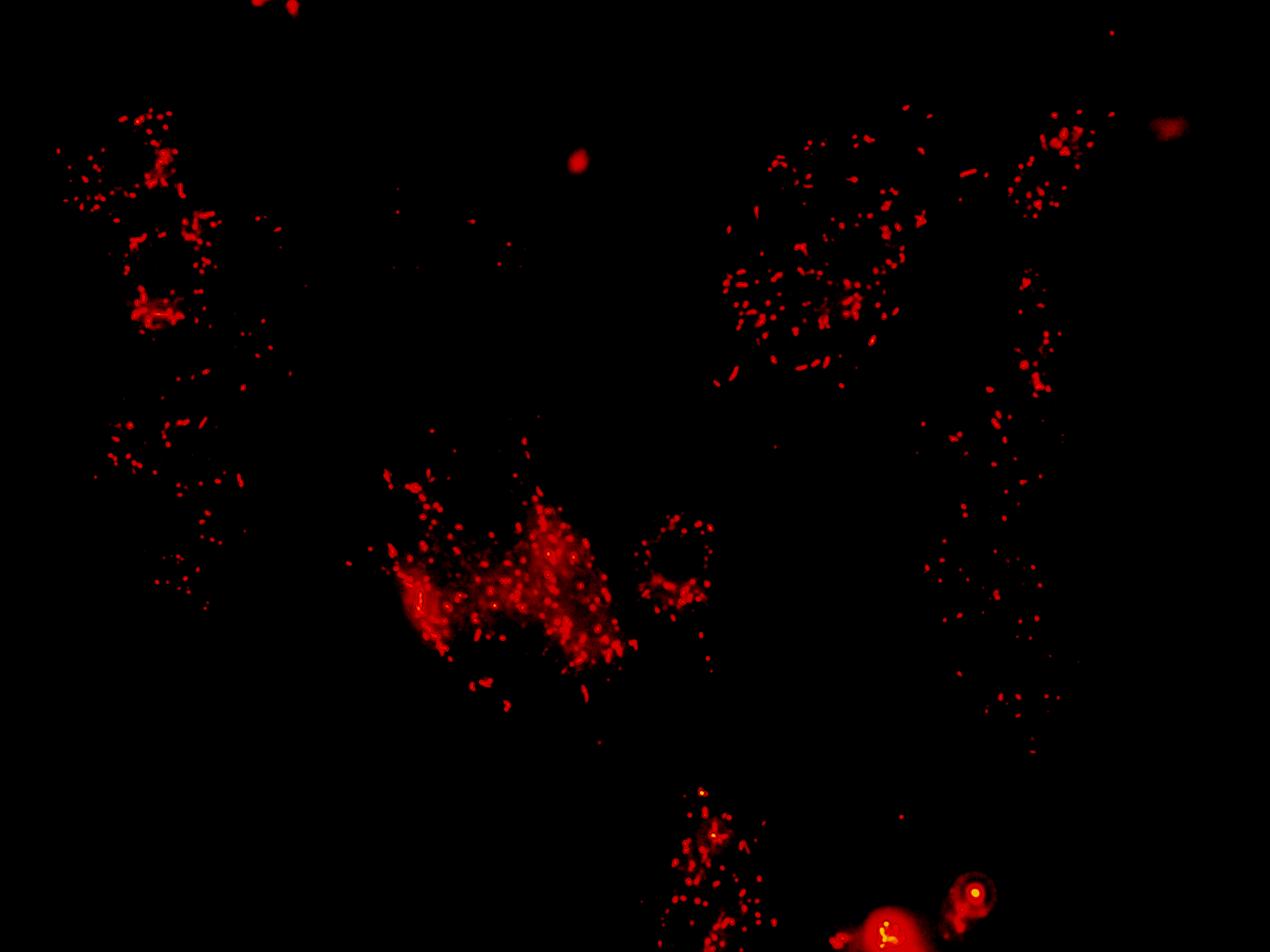

Supplement: Supplementary file 10 — Source Data for Figure 3 [file EMMM-15-e16928-s003.zip › Figure3/3B/H9c2+ImP_Aggregate.tif]

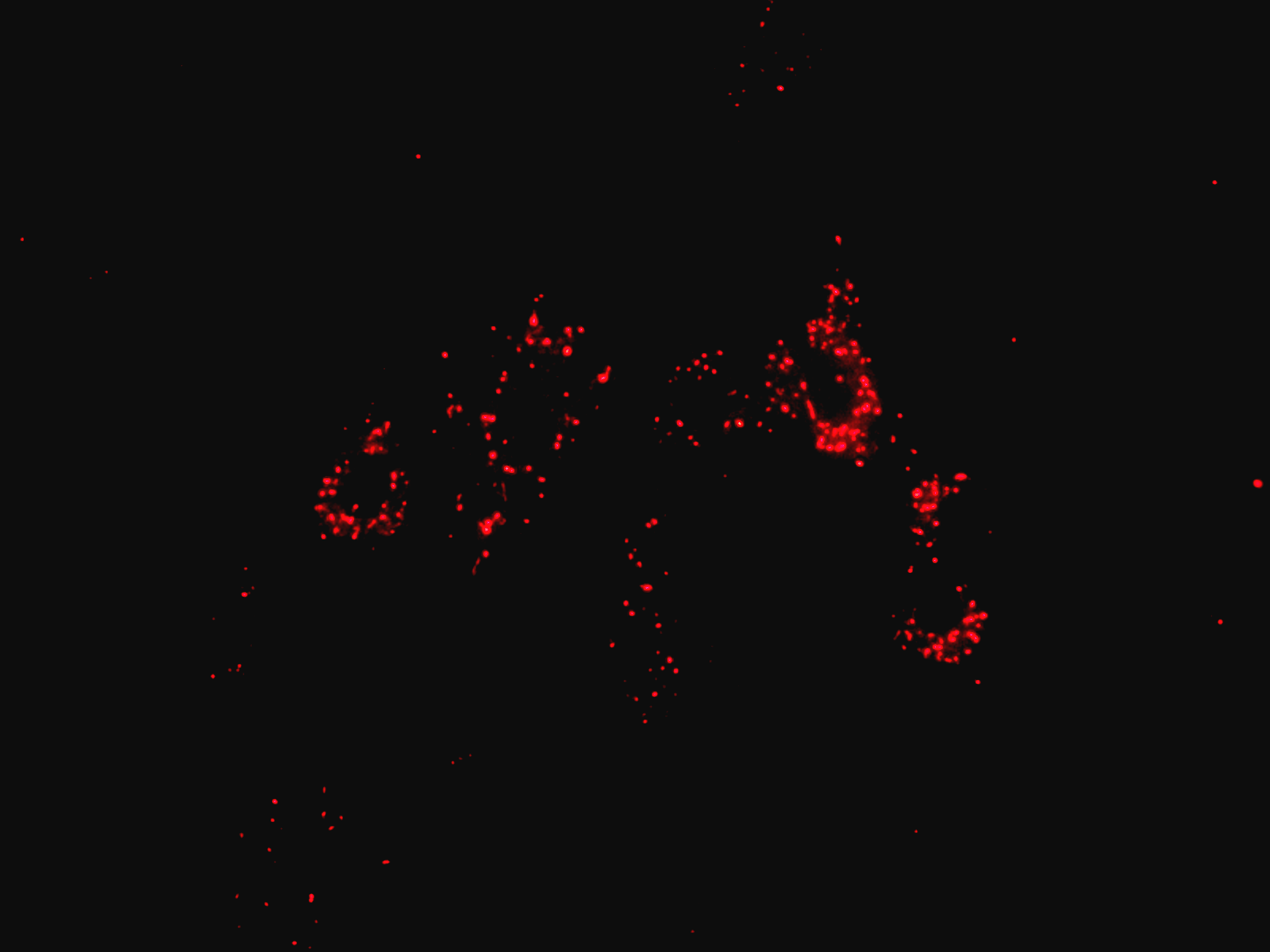

Supplement: Supplementary file 10 — Source Data for Figure 3 [file EMMM-15-e16928-s003.zip › Figure3/3B/H9c2_Aggregate.tif]

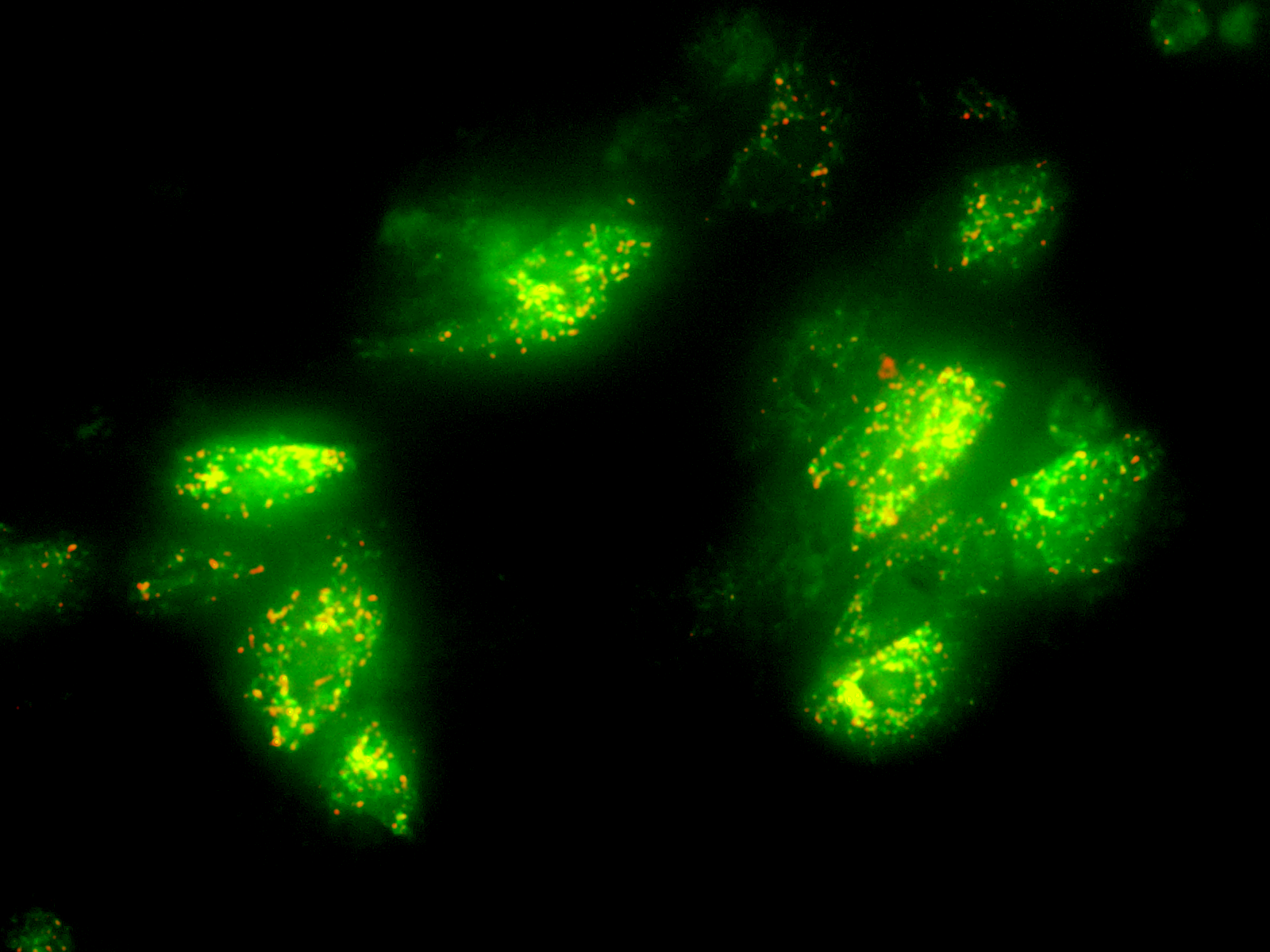

Supplement: Supplementary file 10 — Source Data for Figure 3 [file EMMM-15-e16928-s003.zip › Figure3/3B/H9c2+H:R+ImP_Merged.tif]

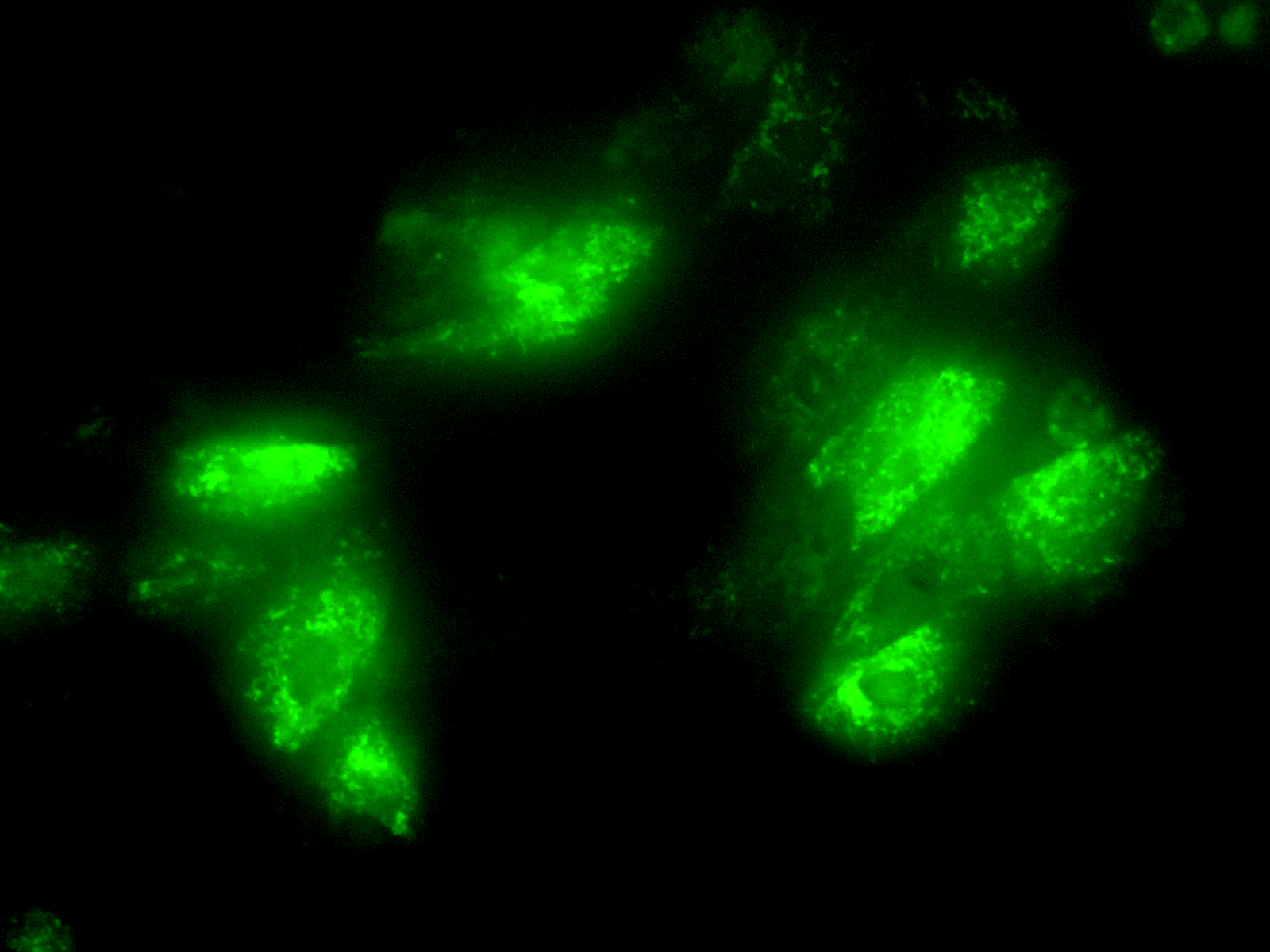

Supplement: Supplementary file 10 — Source Data for Figure 3 [file EMMM-15-e16928-s003.zip › Figure3/3B/H9c2+H:R+ImP_Monomer.tif]
