# Supplementary material for: Microbial metabolites in chronic heart failure and its common comorbidities
Source: EMBO Mol Med. 2023 May 8;15(6):e16928. doi: 10.15252/emmm.202216928 (PMC10245034; doi:10.15252/emmm.202216928)
Supplement: Supplementary file 13 [file EMMM-15-e16928-s011.pdf]

EMBO Molecular Medicine

Microbial metabolites in chronic heart failure and its common comorbidities

Sha Hua, Bomin Lv, Zeping Qiu, Zhuojin Li, Zhiyan Wang, Yanjia Chen, Yanxin Han, Katherine Tucker, Hao Wu, and Wei Jin
DOI: 10.15252/emmm.202216928

Corresponding author(s): Hao Wu (hao_w@fudan.edu.cn) , Wei Jin (jinwei@shsmu.edu.cn)

Review Timeline:

Submission Date:	23rd Sep 22
Editorial Decision:	21st Oct 22
Revision Received:	29th Mar 23
Editorial Decision:	14th Apr 23
Revision Received:	16th Apr 23
Accepted:	18th Apr 23

Editor: Lise Roth

Transaction Report:

(Note: With the exception of the correction of typographical or spelling errors that could be a source of ambiguity, letters and reports are not edited. Depending on transfer agreements, referee reports obtained elsewhere may or may not be included in this compilation. Referee reports are anonymous unless the Referee chooses to sign their reports.)

21st Oct 2022

Dear Dr. Wu,

Thank you for the submission of your manuscript to EMBO Molecular Medicine. We have now received feedback from the three reviewers who agreed to evaluate your manuscript. As you will see below, the reviewers raise substantial concerns on your work, which unfortunately preclude its publication in EMBO Molecular Medicine in its current form.

The reviewers find that the question addressed by the study is of potential interest, however they remain unconvinced that some of the major conclusions are sufficiently supported by the data. They thus raise the following major issues:
- methods must be detailed, and clarification/discussion on several points are needed
- the findings should be supported by wet-lab experiments

We understand that additional rodent experiments as suggested by referee #3 would require a lot of time and effort and we therefore further consulted the referees on this point. We agreed that while further consideration in EMBO Molecular Medicine would require some level of experimental validation, this could be done in cellular models (e.g. cardiomyocytes).

If you feel you can satisfactorily address these points and those listed by the referees, you may wish to submit a revised version of your manuscript. Please attach a covering letter giving details of the way in which you have handled each of the points raised by the referees. A revised manuscript will once again be subject to review and we cannot guarantee at this stage that the eventual outcome will be favorable.

Addressing the reviewers' concerns in full will be necessary for further considering the manuscript in our journal, and acceptance of the manuscript will entail a second round of review. EMBO Molecular Medicine encourages a single round of revision only and therefore, acceptance or rejection of the manuscript will depend on the completeness of your responses included in the next, final version of the manuscript. For this reason, and to save you from any frustrations in the end, I would strongly advise against returning an incomplete revision.

Revised manuscripts should be submitted within three months of a request for revision; they will otherwise be treated as new submissions, except under exceptional circumstances in which a short extension is obtained from the editor.

When submitting your revised manuscript, please carefully review the instructions that follow below. We perform an initial quality control of all revised manuscripts before re-review; failure to include requested items will delay the evaluation of your revision.

We require:

1) A .docx formatted version of the manuscript text (including legends for main figures, EV figures and tables). Please make sure that the changes are highlighted to be clearly visible.

2) Individual production quality figure files as .eps, .tif, .jpg (one file per figure). For guidance, download the 'Figure Guide PDF' (https://www.embopress.org/page/journal/17574684/authorguide#figureformat).

3) At EMBO Press we ask authors to provide source data for the main and EV figures. Our source data coordinator will contact you to discuss which figure panels we would need source data for and will also provide you with helpful tips on how to upload and organize the files.
Numerical data should be provided as individual .xls or .csv files (including a tab describing the data). For blots or microscopy, uncropped images should be submitted (using a zip archive if multiple images need to be supplied for one panel). Additional information on source data and instruction on how to label the files are available at
.

4) A .docx formatted letter INCLUDING the reviewers' reports and your detailed point-by-point responses to their comments. As part of the EMBO Press transparent editorial process, the point-by-point response is part of the Review Process File (RPF), which will be published alongside your paper.

5) A complete author checklist, which you can download from our author guidelines (https://www.embopress.org/page/journal/17574684/authorguide#submissionofrevisions). Please insert information in the checklist that is also reflected in the manuscript. The completed author checklist will also be part of the RPF.

6) Please note that all corresponding authors are required to supply an ORCID ID for their name upon submission of a revised manuscript.

7) It is mandatory to include a 'Data Availability' section after the Materials and Methods. Before submitting your revision, primary datasets produced in this study need to be deposited in an appropriate public database, and the accession numbers and database listed under 'Data Availability'. Please remember to provide a reviewer password if the datasets are not yet public (see https://www.embopress.org/page/journal/17574684/authorguide#dataavailability).

In case you have no data that requires deposition in a public database, please state so in this section. Note that the Data Availability Section is restricted to new primary data that are part of this study.

8) For data quantification: please specify the name of the statistical test used to generate error bars and P values, the number (n) of independent experiments (specify technical or biological replicates) underlying each data point and the test used to calculate p-values in each figure legend. The figure legends should contain a basic description of n, P and the test applied. Graphs must include a description of the bars and the error bars (s.d., s.e.m.). Please provide exact p values.

9) Our journal encourages inclusion of *data citations in the reference list* to directly cite datasets that were re-used and obtained from public databases. Data citations in the article text are distinct from normal bibliographical citations and should directly link to the database records from which the data can be accessed. In the main text, data citations are formatted as follows: "Data ref: Smith et al, 2001" or "Data ref: NCBI Sequence Read Archive PRJNA342805, 2017". In the Reference list, data citations must be labeled with "[DATASET]". A data reference must provide the database name, accession number/identifiers and a resolvable link to the landing page from which the data can be accessed at the end of the reference. Further instructions are available at .

10) We replaced Supplementary Information with Expanded View (EV) Figures and Tables that are collapsible/expandable online. A maximum of 5 EV Figures can be typeset. EV Figures should be cited as 'Figure EV1, Figure EV2" etc... in the text and their respective legends should be included in the main text after the legends of regular figures.
- For the figures that you do NOT wish to display as Expanded View figures, they should be bundled together with their legends in a single PDF file called *Appendix*, which should start with a short Table of Content. Appendix figures should be referred to in the main text as: "Appendix Figure S1, Appendix Figure S2" etc.
- Additional Tables/Datasets should be labeled and referred to as Table EV1, Dataset EV1, etc. Legends have to be provided in a separate tab in case of .xls files. Alternatively, the legend can be supplied as a separate text file (README) and zipped together with the Table/Dataset file.
See detailed instructions here:

11) The paper explained: EMBO Molecular Medicine articles are accompanied by a summary of the articles to emphasize the major findings in the paper and their medical implications for the non-specialist reader. Please provide a draft summary of your article highlighting
- the medical issue you are addressing,
- the results obtained and
- their clinical impact.
This may be edited to ensure that readers understand the significance and context of the research. Please refer to any of our published articles for an example.

12) For more information: There is space at the end of each article to list relevant web links for further consultation by our readers. Could you identify some relevant ones and provide such information as well? Some examples are patient associations, relevant databases, OMIM/proteins/genes links, author's websites, etc...

13) Author contributions: CRediT has replaced the traditional author contributions section because it offers a systematic machine readable author contributions format that allows for more effective research assessment. Please remove the Authors Contributions from the manuscript and use the free text boxes beneath each contributing author's name in our system to add specific details on the author's contribution. More information is available in our guide to authors.

14) Conflict of interest: We updated our journal's competing interests policy in January 2022 and request authors to consider both actual and perceived competing interests. Please review the policy https://www.embopress.org/competing-interests and update your competing interests if necessary.

15) Every published paper now includes a 'Synopsis' to further enhance discoverability. Synopses are displayed on the journal webpage and are freely accessible to all readers. They include a short stand first (maximum of 300 characters, including space) as well as 2-5 one-sentences bullet points that summarizes the paper. Please write the bullet points to summarize the key NEW findings. They should be designed to be complementary to the abstract - i.e. not repeat the same text. We encourage inclusion of key acronyms and quantitative information (maximum of 30 words / bullet point). Please use the passive voice. Please attach these in a separate file or send them by email, we will incorporate them accordingly.

Please also suggest a striking image or visual abstract to illustrate your article as a PNG file 550 px wide x 300-600 px high.

16) As part of the EMBO Publications transparent editorial process initiative (see our Editorial at http://embomolmed.embopress.org/content/2/9/329), EMBO Molecular Medicine will publish online a Review Process File (RPF) to accompany accepted manuscripts.
In the event of acceptance, this file will be published in conjunction with your paper and will include the anonymous referee reports, your point-by-point response and all pertinent correspondence relating to the manuscript. Let us know whether you agree with the publication of the RPF and as here, if you want to remove or not any figures from it prior to publication.
Please note that the Authors checklist will be published at the end of the RPF.

EMBO Molecular Medicine has a "scooping protection" policy, whereby similar findings that are published by others during review or revision are not a criterion for rejection. Should you decide to submit a revised version, I do ask that you get in touch after three months if you have not completed it, to update us on the status.

I look forward to receiving your revised manuscript.

Yours sincerely,

Lise Roth

Lise Roth, PhD
Senior Editor
EMBO Molecular Medicine

***** Reviewer's comments *****

Referee #1 (Comments on Novelty/Model System for Author):

It is difficult to evaluate technical quality as too much information has been left out in the methods and the results are not described in a detailed manner. The study design needs to be better rationalised and statistical analysis should be more stringent.

Referee #1 (Remarks for Author):

Hua et al. describe the serum profile of microbial metabolites in groups of Chinese individuals with varying degree of cardiometabolic multi-morbidities. The study follows-up on findings from previous studies that have described shared metabolomics patterns in individuals with CHF, T2D and CKD in different populations.

The questions the study sets out to address are promising and it is certainly of interest to evaluate these microbial metabolite contributions to CHF and comorbidities in the Chinese population. There are both some validated and novel metabolite-disease associations described in the study that the authors have put into context with the literature. The figures are well made and informative. However, a lot of details regarding the study design, methodology and results are unclear or left out, making it difficult to evaluate the quality of the work. My main comments are the following:

1. The study design is complex, having 6 groups and multiple outcomes. The authors need to explain much better their analysis strategy, i.e. how these groups are compared to answer the research questions, for example to define and validate disease-shared or disease-specific metabolites. In the validation analysis, are the metabolites associating with the same outcome here as in the discovery cohorts?
2. The NGT vs NGT+CHF is the only clean comparison for CHF relevance, thus metabolite changes that are not observed here but only in comparisons with multi-morbid groups cannot be attributed to CHF with any certainty. This seems to be the case for a few of the metabolites highlighted in the text like 3−Hydroxybutyric acid, C18:1 (oleic acid and C18:2 (linoleic acid). I think the authors need to be more careful in their interpretations of some of the findings.
3. The significance criteria that I could find information on is overly relaxed. Using unadjusted $P<0.05$, and in some places <0.1, is not really valid for this number of tests. The authors should use some correction for multiple testing and focus on those results. It is fine to describe some findings that are nominally significant, especially if it is a validation of previously published results, or if they are consistent across different comparisons between groups, but the multiple-test correction should be

available and clear to the reader.
4. The methods section is really lacking. The metabolomics quantification and QC needs to be described at least briefly, even if published elsewhere. The analyses need to be described in terms of what exactly is being tested and how (method, models, covariates, parameter settings, significance criteria etc).
5. The results should in general be provided in more detail in tables and supplementary tables (rather than only figures), for example to be able to see which metabolites are associated with which condition or were validated, and full results with effect sizes, confidence intervals, p-values from cross-group comparison, the mediation analysis, survival analysis etc.
6. How many events were there for survival analysis on mortality or CHF rehospitalization?

Minor
• The authors highlight the variability of the metabolite levels in the cohort as a main finding. Is this variation unexpected and if so why?
• Fig 1, add vertical lines at HR=1 for easier evaluation of effect size, show confidence intervals for HRs and add some significance legend. What are group 1,2,3 in the BPRHS cohort?
• Fig 2,difficult to infer which P values apply to which comparison
• How many individuals were within each CHF subgroup?
• Are there any differences in the Chinese cohort recruitment strategy or baseline characteristics that could describe the differences in baseline values of ImP compared to the Swedish? Is this the only metabolite that differed between cohorts in this manner?
• The AUC values for the metabolite score is most likely overfitted as trained and tested on the same cohort. If external validation is not possible then at least some bootstrapping would be appropriate if the sample size allows. In any case this limitation should be acknowledged in the discussion.

Referee #2 (Comments on Novelty/Model System for Author):

Further validation of the findings should be performed.

Referee #2 (Remarks for Author):

The work by Hua et al shows that metabolite signatures from 260 chinese individuals could be used to classify chronic heart failure and associated diseases such as chronic kidney disease and type 2 diabetes. Overall, the computational analyses are interesting but the work remains purely descriptive and given the low number of patients in this cohort one wonders about the wide applicability of these results. Some major points should be address before this study is considered for publication at EMBO MM.

A careful look at the classification of the different metabolites (figure S3 and Figure 1) according to the class they belong to shows that these have been randomly placed in the wrong categories. Eg. Kyrunerine is not an aminoacid (despite deriving from one); isovaleric acid is not ana aa but a short chain fatty acid, acetic acid is not a lipid but a fermentation product and a carboxylic acid (as most TCA cycle intermediates which here have been categorised as Energy!. These are some examples but I'd say that roughly 30% of the metabolites have been misassigned. The authors need to carefully place these into their adequate super pathways using well established databases. This should be done consistently throughout. The authors should also categorise these metabolites in their respective KEGG pathways (some metabolites will belong to more than one KEGG pathway). This will allow for KEGG functional enrichment analysis and network analysis which should be additionally performed.

It is not clear why the metabolites that "are not" from microbial origin have been ignored. If data are available, as it should be, since the Q300 platform has been used and these can detect the additional metabolites it would have been interesting to investigate changes between the different cohorts. It would be even more valuable if the metabolites were classed as host, host and microbe and microbial only origin.

These analyses have not been done in cohorts for which diet has been factored in. Given that diet strongly remodels the microbiota and hence their metabolome, without this level of information it is hard to assign changes in the metabolome purely due to disease status rather than dietary cues that may be directly leading to these diseases in the first place. This should be acknowledged and discussed.

The authors mention the use of machine learning analyses to address causation. Causation cannot be established computationally. This should be reworded appropriately. The authors focus on Imp which puzzles me since the mean effect sizes of Imp concentrations between different conditions is fairly small for metabolites changes according to the different conditions and at these changes very unlikely to exert any causal role in the progression of disease. At the very best, it may be a biomarker, but given the variability and differences measured, a not very good one either. What seems to stand out is an enrichment for metabolites involved in the first part of the TCA cycle (citric acid, isocitric acid, aconitic acid). This is not explained or investigated further, when it should have been.

Additional wet lab experimental work to validate some of the hypothesis being put forward would strongly validate this study (For

example, showing in a cell system that changes in metabolite at concentrations found in this study can cause alterations in physiological/signalling parameters)

Referee #3 (Comments on Novelty/Model System for Author):

The manuscript is generally interesting. But the current version lacks sufficient details in methods for precise quality evaluation.

Referee #3 (Remarks for Author):

Summary:
Hua et al. report the alterations in gut microbially derived metabolites are linked to CHF and its two common comorbidities, that are type 2 diabetes and chronic kidney disease. The profiling of 151 metabolites in a cohort of 260 individuals were based on absolute quantification, which resulted in a big variation in the levels of measured metabolites across the cohort. The 98 CHF-associated metabolites were validated in external study materials that are geographically independent. In addition, the authors highlighted imidazole propionate, a bacterial metabolite with 3 folds of basal levels in Chinese than that in Swedish cohort. Finally, as the authors showed, metabolites-based biomarkers provide prognostic superiority over the traditional risk scores for CHF.

Major comments:
1. Both discovery and replication study samples are of adequate size and as such, the epidemiological studies seem statistically powered. The presented observational and cross-sectional studies are however, purely descriptive and the interpretations of identified CHF-, T2D-, or CKD-related metabolites remain speculative. Substantial supportive evidence seems needed to interpret the reported relationships between gut microbially derived metabolites and onset of T2D, CHF, or CKD. Along the same line, the descriptive nature of the current studies would benefit from fecal microbiota transfer studies from individuals with CHF-, T2D, or CKD-phenotypes to germ-free rodents to explore mechanisms in depth behind potential inducible changes.
2. It's unfortunate to miss the profile of bile acids (as far as I can see, only chenodeoxycholic acid, chenodeoxyglycocholic acid, and glycolithocholic acid were included), short chain fatty acids (only acetate was measured), and trimethylamine N-oxide (absent), which had been widely reported as microbiota-related metabolites linked to insulin resistance, T2D, or cardiometabolic diseases. Please clarify why these metabolites were omitted in the current work.
3. It is well-known that multiple drugs and drug combinations cause considerable perturbation of gut microbiome composition and function. Although the investigators report that the study had excluded the effects of metformin on metabolome, but not clear how the impact of metformin was deconfounded. Still, such large number of individuals in real life settings both in discovery cohort undoubtedly take drugs including proton pump inhibitors, statins, antihypertensives, antinflammatory drugs, pain killers, Chinese herbal medicine etc, - all with potentially major confounding impact on microbiome related metabolites. Therefore, authors are strongly encouraged to provide information on medication use and to apply a state-of-the art drug-deconfounding pipeline in the analyses of their data.
4. Current method lacks far less sufficient details. For example, page 11, how many individuals completed the follow up period? When was the plasma collected after obtaining blood samples from participants? How was the plasma stored? More importantly, how was the targeted metabolomics performed? The reference provided by the authors was about metabolomics profiling based on GC-Q/TOF-MS platform, whereas in the manuscript, they say a UPLC-MS/MS system was used. In statistical analysis, how was the p values adjusted?Without such details, it is impossible to evaluate the reliability of the presented outcomes.

Minor comments:
1. The title should be more specified as this manuscript only talks about microbially related metabolites.
2. Figure 2 legend, here raw p value threshold was set at 0.1, why not 0.05?
3. Figure 3A, how was the association analysis performed? Was gender adjusted?
4. Results should be shortened; the biological interpretation of disease-associated metabolites should be moved to discussion.

******* Reviewer's comments *******

Referee #1 (Comments on Novelty/Model System for Author):

It is difficult to evaluate technical quality as too much information has been left out in the methods and the results are not described in a detailed manner. The study design needs to be better rationalized and statistical analysis should be more stringent.

Response: We thank the reviewer for these comments. In response to the reviewer's feedback, we have comprehensively revised the manuscript, with a focus on enhancing the clarity and structure of our results and discussions. We have also included detailed computational and experimental methods in the methods section. Additionally, we have adjusted the raw P values using the Benjamini-Hocherg method and expanded on the rationale behind the study design. We hope that these revisions have addressed the reviewer's concerns and are described in detail below.

Referee #1 (Remarks for Author):

Hua et al. describe the serum profile of microbial metabolites in groups of Chinese individuals with varying degree of cardiometabolic multi-morbidities. The study follows-up on findings from previous studies that have described shared metabolomics patterns in individuals with CHF, T2D and CKD in different populations.

The questions the study sets out to address are promising and it is certainly of interest to evaluate these microbial metabolite contributions to CHF and comorbidities in the Chinese population. There are both some validated and novel metabolite-disease associations described in the study that the authors have put into context with the literature. The figures are well made and informative. However, a lot of details regarding the study design, methodology and results are unclear or left out, making it difficult to evaluate the quality of the work.

Response: We thank the reviewer for these positive comments! We have now revised manuscript as suggested by the reviewer.

My main comments are the following:

1. The study design is complex, having 6 groups and multiple outcomes. The authors need to explain much better their analysis strategy, i.e. how these groups are compared to answer the research questions, for example to define and validate disease-shared or disease-specific metabolites. In the validation analysis, are the metabolites associating with the same outcome here as in the discovery cohorts?

Response: we apologize for any confusion caused by our lack of clarity. To address this issue, we have expanded on the rationale behind our study design in the first paragraph of the results section which now reads:

"Characterization of prediabetes or intermediate hyperglycaemia presents a unique opportunity for studying the role of gut microbiota in the progression to clinical T2D. We and others have demonstrated the potential contribution of gut microbiota in this transition (Wu et al, 2020; Zhou et al, 2019). To further explore the molecular signatures that may link CHF and T2D development, we screened 260 individuals with varying glucose metabolism from the REM-HF cohort and conducted a targeted metabolomics analysis..."

Definition for the cross-group comparisons to identify the disease-shared or disease-specific metabolites have also been added to the text:

"...We additionally performed cross-group comparisons to identify potential disease-specific metabolites. Our results revealed that 19, 13, and 35 metabolites were unique to CHF (NGT+CHF versus NGT), prediabetes/T2D (Prediabetes/T2D+CHF versus NGT+CHF), and CKD (Prediabetes/T2D+CHF+CKD versus Prediabetes/T2D+CHF), respectively..."

Regarding the two validation cohorts, the EPIC-Norfolk cohort used the same outcomes, which included the incidence of all three cardiometabolic diseases, as well as the hazards ratio for heart failure rehospitalization and cardiovascular deaths. In contrast, the BPRHS cohort utilized a relevant outcome composite that comprised of nine components associated with metabolic, inflammatory, and neurohormonal states. Those information have been added to the methods section.

2. The NGT vs NGT+CHF is the only clean comparison for CHF relevance, thus metabolite changes that are not observed here but only in comparisons with multi-morbid groups cannot be attributed to CHF with any certainty. This seems to be the case for a few of the metabolites highlighted in the text like 3−Hydroxybutyric acid, C18:1 (oleic acid and C18:2 (linoleic acid). I think the authors need to be more careful in their interpretations of some of the findings.

Response: we appreciate the reviewer for bringing this to our attention. To improve the clarity of our manuscript, we have rephrased this paragraph and other related results and discussions. Additionally, we have conducted *in vitro* cell experiments to validate some of the metabolite-disease links in cardiomyoblasts, as suggested by the other two reviewers. Specifically, we found that 3-hydroxybutyric acid, but not adrenic acid (C22:4), induced higher *NPPB* (BNP-encoding gene) expression than the control. We have included these results and revised our manuscript accordingly.

The related results part now reads:

"... Most lipid derived metabolites were insignificant in individuals with CHF compared to the NGT control group but showed progressively increased patterns along with co-ocurring CHF morbidities. For example, we noted that the serum levels for most long-chain fatty acids, including the two most abundant ones C18:1 (oleic acid) and C18:2 (linoleic acid) that can be taken up by the heart, were increased in the CHF+prediabetes/T2D groups but showed the most pronounced increase in individuals with all three diseases (Figure 1). C18:1 (oleic acid) and C22:4 (adrenic acid) additionally showed higher hazard ratios for incidence of CHF rehospitalization or cardiovascular deaths... "

The related discussion now reads:

"The failing heart also associated with reduced FFA utilization, which accounts for >70% myocardial carbon sources (Murashige et al., 2020). It has been shown that, for every standard deviation increase in serum FFA reflecting reduced heart consumption, there was a 12% higher incidence of CHF (Djousse et al, 2013). Similarly, we found the serum levels of long-chain fatty acid adrenic acid (C22:4) and short-chain fatty acid acetic acid were both increased in most disease groups and associated with higher hazard ratios for incidence of CHF rehospitalization and cardiovascular deaths, consistent with others (Delgado et al, 2017; Lankinen et al, 2015). However, no impact on NPPB gene expression levels were observed for both metabolites at physiological doses when exposed to cardiomyoblasts..."

3. The significance criteria that I could find information on is overly relaxed. Using unadjusted P<0.05, and in some places <0.1, is not really valid for this number of tests. The authors should use some correction for multiple testing and focus on those results. It is fine to describe some findings that are nominally significant, especially if it is a validation of previously published results, or if they are consistent across different comparisons between groups, but the multiple-test correction should be available and clear to the reader.

Response: We appreciate the reviewer's suggestions and agree with their concerns regarding multiple testing. To address this issue, we have performed the Benjamini-Hochberg correction and removed all metabolites with adjusted P values >= 0.1 for further analysis, unless the raw P value was <0.05 and there was at least one validation cohort supporting its link to the corresponding disease. We have also included both raw P values and adjusted P values in the supplementary tables and online webservers (https://omicsdata.org/Apps/REM-HF/Default). Changes to the text reflecting these modifications have been made in our revised manuscript.

Figure below shows both the raw and adjusted P values for 3-hydroxybutyric acid to illustrate the changes on our webserver:

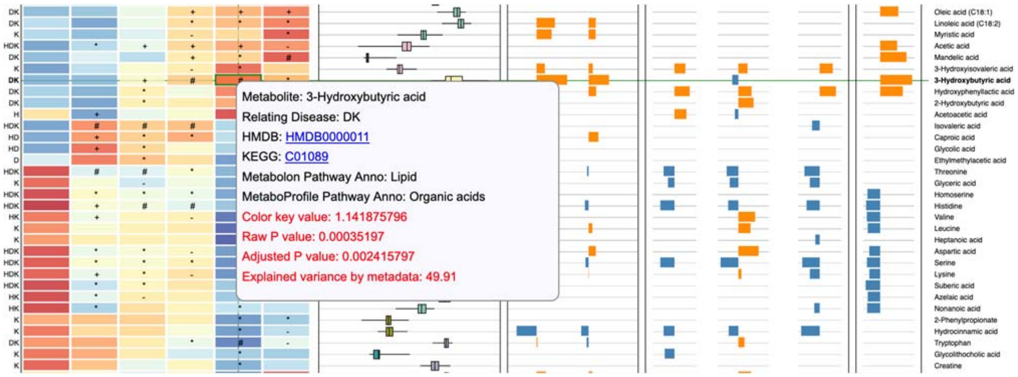

In the results, it now reads:

"Compared to the NGT controls, the circulating levels of 94 microbial metabolites were significantly altered in at least one disease setting, after adjusting for age and sex differences (adjusted P value < 0.1). Additionally, two metabolites, myristic acid (C14:0) and 2/alpha-aminobutyric acid, showed altered concentrations based only on the raw P values, but with supporting evidence from the validation cohorts (Table EV2)... "

4. The methods section is really lacking. The metabolomics quantification and QC needs to be described at least briefly, even if published elsewhere. The analyses need to be described in terms of what exactly is being tested and how (method, models, covariates, parameter settings, significance criteria etc).

Response: Yes. We have revised the methods part as suggested. The metabolomics part now reads:

"**Targeted metabolomics profiling.** The blood samples were centrifuged at 1,000g for 10 min to separate the serum within 12 hours after collection, and the aliquoted serum samples were stored at -80 °C for further analysis. Absolute quantification of the microbially associated metabolites was conducted based on the Q300 platform (Metabo-Profile Biotechnology, Shanghai, China) as previously described (Xie et al., 2021). In brief, 25 μL serum samples in a 96-well plate was mixed with 120 μL methanol and then vortexed vigorously for five minutes and centrifuged at 4,000g for 30 minutes. 30 μL of supernatant and 20 μL of freshly prepared derivative reagents were added to a clean 96-well plate for further derivatization. The samples were further diluted using 330 μL of ice-cod 50% methanol solution and centrifuged. 135 μL of supernatant was transferred to a new 96-well plate with 10 μL internal standards in each well. Measurement was performed using a UPLC-MS/MS system (ACQUITY UPLC-Xevo TQ-S, Waters Corp., Milford, MA, USA) with the following settings: BEHC18 1.7 μM VanGuard pre-column and analytical column; water with 0.1% formic acid for mobile phase A and acetonitrile/IPA for mobile phase B; flow rate at 0.4 mL/min; and capillary (Kv)=1.5 (ESI+), 2.0 (ESI-). The raw

data files generated by UPLC-MS/MS were processed using the MassLynx software (v4.1) to perform peak integration, calibration, and quantification for each metabolite."

The statistical analysis part now reads:

"**Statistical analyses.** All statistical analyses were conducted in the R environment (version 4.0.4). The nonparametric Wilcox rank-sum test was used to identify metabolites that differed significantly between groups. For categorical variables, the chi-squared test was used. Drug deconfounding analysis for the metabolite-disease links was performed using the metadeconfoundR package (v0.1.8) (Forslund et al., 2021). The multivariable Cox proportional hazards model (survival v3.2.13) (Therneau, 2019) and random forest model (randomForest v4.6.14) (Liaw & Wiener, 2002) with sex adjustment (ntree=3000, mtry=2-30) were utilized. Bi-directional mediation analysis (mediation v4.5.0) (Tingley et al, 2014) was performed with age and sex adjustment. Kaplan-Meier survival analysis (survminer v0.4.9) (Kassambara et al, 2021) and time-dependent ROC curves (riskRegression v2021.10.10) (Gerds & Kattan, 2021) were conducted with bootstrap resampling (n=1000). The log-rank test was applied to compare survival curves between groups. Raw P values were adjusted by the Benjamini-Hochberg method (Benjamini & Hochberg, 1995). Adjusted P values < 0.1 and raw P values < 0.05 were considered statistically significant**."**

5. The results should in general be provided in more detail in tables and supplementary tables (rather than only figures), for example to be able to see which metabolites are associated with which condition or were validated, and full results with effect sizes, confidence intervals, p-values from cross-group comparison, the mediation analysis, survival analysis etc.

Response: We thank the reviewer for this suggestion. We have now added those information in Table EV2 and provided source data for all models and figures.

6. How many events were there for survival analysis on mortality or CHF rehospitalization?

Response: During two-years of follow-up, 89 CHF rehospitalization evens and 24 cardiovascular deaths have been reported. We have now added those information into the text. It now reads:

"...A total of 244 individuals completed the follow-up study with 89 CHF rehospitalization and 24 cardiovascular deaths reported... "

Minor

• The authors highlight the variability of the metabolite levels in the cohort as a main finding. Is this variation unexpected and if so why?

Response: Yes. The absolute concentrations and variations of most metabolites in humans are currently unknown. We think the physiological variation of each metabolite may reflect its associations with health and disease.

• Fig 1, add vertical lines at HR=1 for easier evaluation of effect size, show confidence intervals for HRs and add some significance legend. What are group 1,2,3 in the BPRHS cohort?

Response: Yes. We have modified the figure and legend accordingly. Calculation of the cardiometabolic stress index and associated grouping methods have also been added to the methods part. It now reads:

"... The cardiometabolic stress (CM) index in BPRHS cohort was calculated as previously described (Murthy *et al*., 2020). This index is based on nine components associated with metabolic, inflammatory, and neurohormonal states, with a score of 0, 1, or 2 assigned to each component. The scores are then summed up to obtain a score ranging from 0 to 11 defined as the CM index. To identify metabolites associated with this index, we divided participants in BPRHS cohort into the following three groups: the low risk group (group1, $0 \leq$ CM index ≤ 3, N = 212), the intermediate risk group (group2, $4 \leq$ CM index ≤ 5, N = 301) and the high risk group (group3, $6 \leq$ CM index, N = 227). We calculated the fold-change of each metabolite by comparing its abundance in group 2 or group 3 to group 1, respectively."

• Fig 2, difficult to infer which P values apply to which comparison

Response: We are sorry for this unclarity. We have added the cross-group comparison in details in both the text and figure legend. In the text it now reads:

"... Our results revealed that 19, 13, and 35 metabolites were unique to CHF (NGT+CHF versus NGT), prediabetes/T2D (Prediabetes/T2D+CHF versus NGT+CHF), and CKD (Prediabetes/T2D+CHF+CKD versus Prediabetes/T2D+CHF), respectively... "

• How many individuals were within each CHF subgroup?

Response: We have 237 individuals with CHF and associated comorbidities, including 66 with HFpEF, 68 with HFmEF and 103 with HFrEF. We have now added this information in the revised manuscript and figure legend. Figure 2 legend now reads:

"...HFpEF: LVEF $\geq$50, n = 66; HFmEF: 40<LVEF<50, n = 68; HFrEF: LVEF $\leq$40, n = 103..."

• Are there any differences in the Chinese cohort recruitment strategy or baseline characteristics that could describe the differences in baseline values of ImP compared to the Swedish? Is this the only metabolite that differed between cohorts in this manner?

Response: We have confirmed that the NGT groups from Sweden and China have comparable age, BMI, and sex distributions. However, we would like to clarify that the full metabolomics data from our Swedish cohort has not been published yet, with the exception of ImP.

The related part in the text now reads:

"...comparable baseline characteristics including male/female ratio, average age and BMI were found between two NGT groups (55/70, 57.6, and 25.5 in Swedish and 12/11, 53.6, and 23.9 in Chinese NGT groups, respectively). "

• The AUC values for the metabolite score is most likely overfitted as trained and tested on the same cohort. If external validation is not possible then at least some bootstrapping would be appropriate if the sample size allows. In any case this limitation should be acknowledged in the discussion.

Response: We apologize for the lack of clarity in our previous statement. We would like to clarify that the AUC values in our studies were actually determined using bootstrapping with 1,000 iterations. We have updated the methods section and figure legend to reflect this information. Figure 5 legend now reads:

"...The AUC values were determined by bootstrap resampling with 1,000 iterations."

Referee #2 (Comments on Novelty/Model System for Author):

Further validation of the findings should be performed.

Response: We thank the reviewer for this valuable suggestion and we have now performed cell-metabolite co-culturing experiments to validate some of those metabolite-disease links as described in detail below.

Referee #2 (Remarks for Author):

The work by Hua et al shows that metabolite signatures from 260 Chinese individuals could be used to classify chronic heart failure and associated diseases such as chronic kidney disease and type 2 diabetes. Overall, the computational analyses are interesting but the work remains purely descriptive and given the low number of patients in this cohort one wonders about the wide applicability of these results. Some major points should be address before this study is considered for publication at EMBO MM.

Response: Many thanks for the reviewer for providing those valuable feedbacks on our manuscript. We have carefully considered and addressed each of the specific comments, resulting in substantial revisions to our study. We believe that the manuscript has greatly improved and hope it now addresses the reviewer's concerns.

A careful look at the classification of the different metabolites (figure S3 and Figure 1) according to the class they belong to shows that these have been randomly placed in the wrong categories. Eg. Kyrunerine is not an amino acid (despite deriving from one); isovaleric acid is not ana aa but a short chain fatty acid, acetic acid is not a lipid but a fermentation product and a carboxylic acid (as most TCA cycle intermediates which here have been categorised as Energy!. These are some examples but I'd say that roughly 30% of the metabolites have been misassigned. The authors need to carefully place these into their adequate super pathways using well established databases. This should be done consistently throughout. The authors should also categorise these metabolites in their respective KEGG pathways (some metabolites will belong to more than one KEGG pathway). This will allow for KEGG functional enrichment analysis and network analysis which should be additionally performed.

Response: We apologize for the confusion here. We would like to explain that the original pathway annotation for each metabolite was based on the Metabolon platform. For clarity, we have added annotation from both Metabolon and Metaboprofile on our webserver, along with links to HMDB and KEGG, as shown in the figure below. In addition, we have updated Figure 1 in the main text to include the Metaboprofile

annotation as suggested by the reviewer. We hope these changes will clarify the pathway annotation for each metabolite.

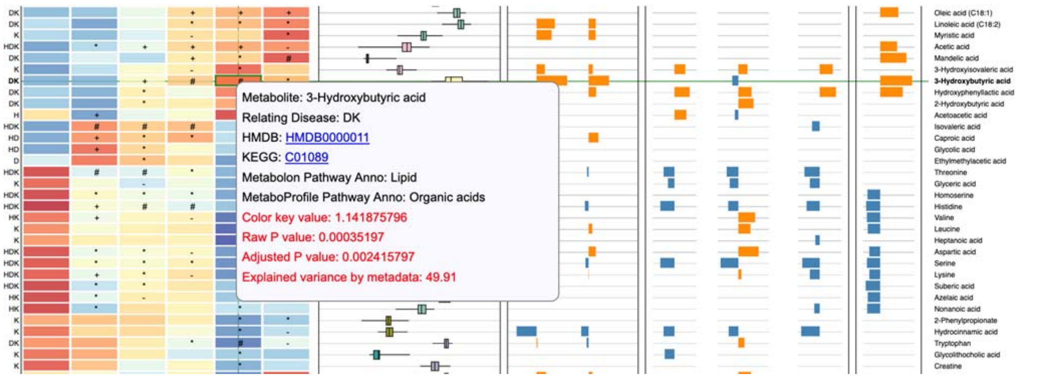

Metabolite pathway annotation on our webserver.

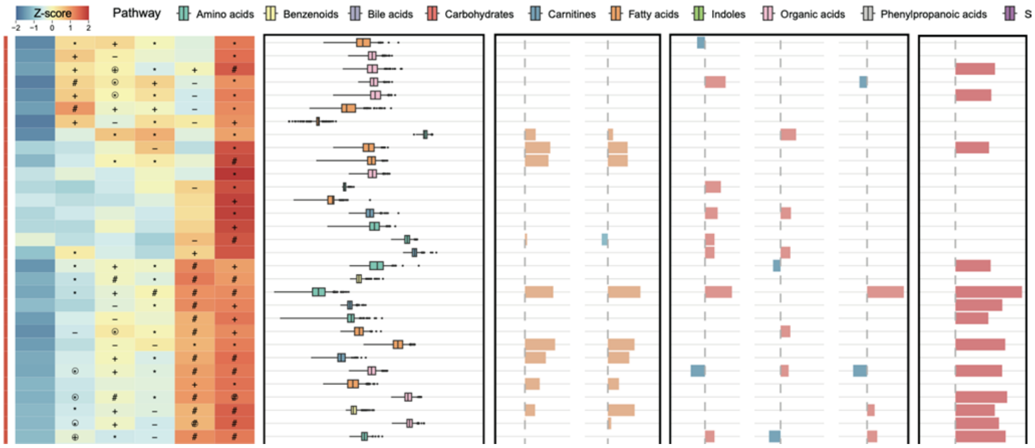

Updated Figure 1 with MetaboProfile pathway annotation for each metabolite (please notice that only part of the figure was shown).

There are several known pipelines including 3Mcor (Sun T. *et al.*, 2022) and we also have our own inhouse method to address this. However, we noticed that the pathway enrichment analysis is heavily dependent on the background metabolite database which could lead to substantial differences across pipelines. This is in contrast with the gene pathway enrichment which includes all known encoding genes within the host genome. Therefore, in this manuscript we still discussed the metabolites grouped by different categories highlighted several important pathways in the manuscript such as those involved in the phenylalanine metabolism and the TCA. We thank the viewer for this suggestion.

It is not clear why the metabolites that "are not" from microbial origin have been ignored. If data are available, as it should be, since the Q300 platform has been used and these can detect the additional metabolites it would have been interesting to

investigate changes between the different cohorts. It would be even more valuable if the metabolites were classed as host, host and microbe and microbial only origin.

Response: We thank the reviewer for bringing this up. We identified a total of 199 metabolites, out of which 151 were classified as microbially associated in our manuscript (**Table EV1**). However, some of these metabolites may also be host-microbe co-metabolites, and it may be difficult to determine whether they are solely microbial in origin without experimental validation. Imidazole propionate is an example of a microbial-only metabolite that has been proven. The majority of the remaining host-specific metabolites did not show significant associations with the studied cardiometabolic diseases, except in individuals with CKD. While these metabolites are listed in **Table EV1**, we did not expand on their discussion as our focus in this study was on the microbially associated ones. We hope this provides clarification. The relative changes for the host-specific metabolites are shown below:

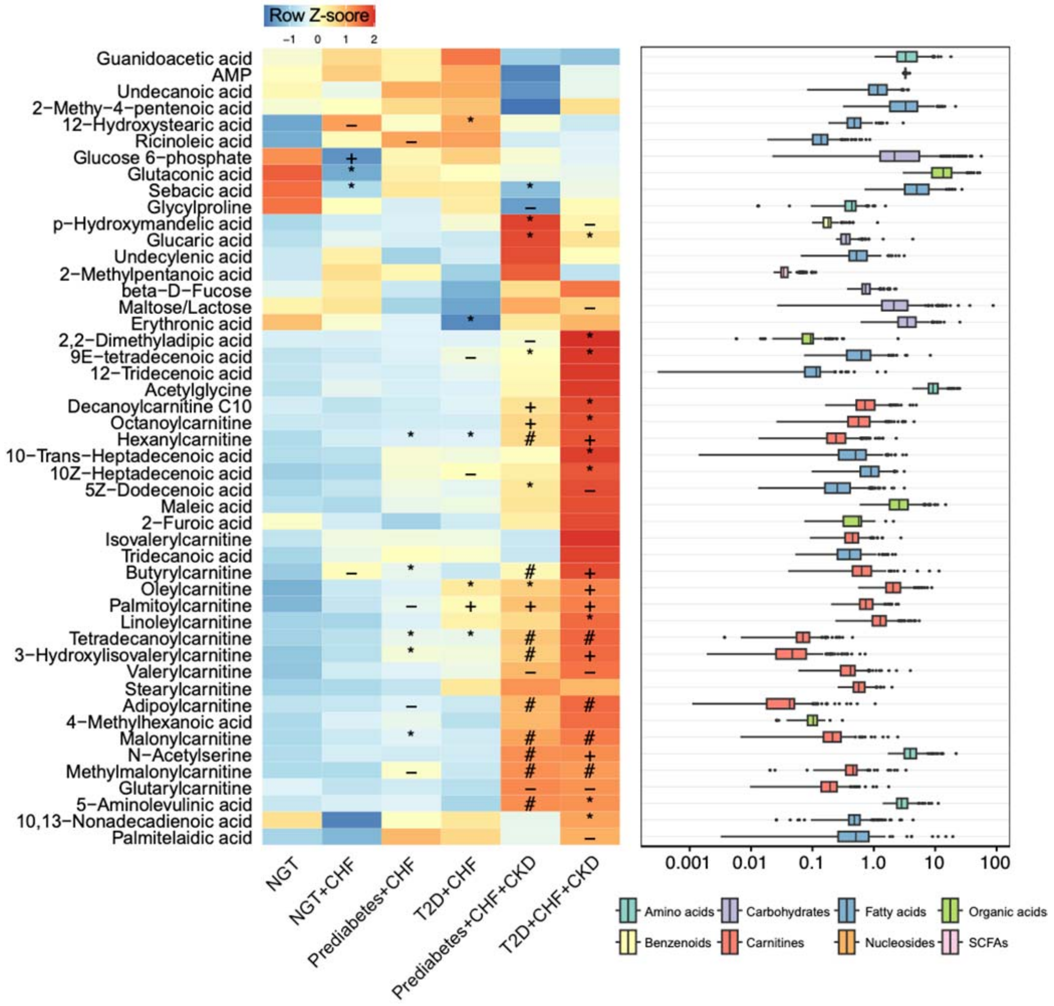

Changes of host-specific metabolites in different disease groups vs. the NGT control group.

These analyses have not been done in cohorts for which diet has been factored in. Given that diet strongly remodels the microbiota and hence their metabolome, without this level of information it is hard to assign changes in the metabolome purely due to disease status rather than dietary cues that may be directly leading to these diseases in the first place. This should be acknowledged and discussed.

Response: We totally agree with the reviewer for this issue. We have acknowledged and discussed this in the text which reads:

"... In line with this, inadequate protein intake, absorption, and negative nitrogen balance have been well documented (Aquilani *et al*, 2003; Arutyunov *et al*, 2008) and recently associated with higher CHF mortality (Streng *et al.*, 2022), despite the fact that a detailed food frequency questionnaire was not available in our cohort."

And also in the limitation part:

"...the lack of detailed nutritional data prevented us from determining whether the altered serum molecules were due to diet or the diseases or both."

The authors mention the use of machine learning analyses to address causation. Causation cannot be established computationally. This should be reworded appropriately. The authors focus on Imp which puzzles me since the mean effect sizes of Imp concentrations between different conditions is fairly small for metabolites changes according to the different conditions and at these changes very unlikely to exert any causal role in the progression of disease. At the very best, it may be a biomarker, but given the variability and differences measured, a not very good one either. What seems to stand out is an enrichment for metabolites involved in the first part of the TCA cycle (citric acid, isocitric acid, aconitic acid). This is not explained or investigated further, when it should have been. Additional wet lab experimental work to validate some of the hypothesis being put forward would strongly validate this study (For example, showing in a cell system that changes in metabolite at concentrations found in this study can cause alterations in physiological/signalling parameters)

Response: We appreciate those feedbacks from the reviewer. The ImP actually varies 100 fold. Our original study demonstrated that this metabolite could lead to insulin resistance via mTORC1 signaling pathway (Koh *et al*. Cell. 2018). However, we were surprised to find that it showed much stronger associations with biomarkers of CHF and CKD than with T2D in this cohort. We have now provided more evidence supporting that this metabolite might be causal to CHF relevant phenotypes in cell

experiments and could disrupt cardiomyoblast functions as reflected by reduced mitochondrial membrane potential upon exposure with ImP. We hope this now addresses the reviewer's concern. Thanks again for this constructive comment.

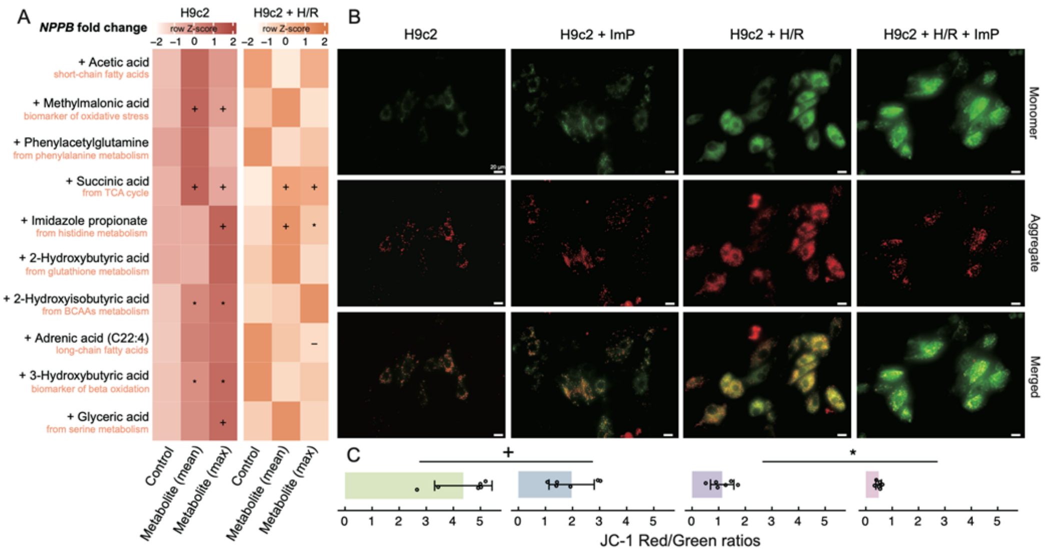

Figure 3. Cardiomyoblast-metabolite coculturing and induction of NPPB gene expression. A, the relative expression of NPPB (scaled by row) upon different metabolites exposure in H9c2 cells pretreated with or without hypoxia/reoxygenation (H/R; six replicates per group). B, representative JC-1 staining images showing red fluorescence of JC-1 aggregates and green signal of monomers. Scale bars: 20 µm. C, quantification of mitochondrial membrane potential (n=6). The data are shown as mean ± s.d; Wilcoxon rank-sum test (-P < 0.1, *P < 0.05, +P < 0.01).

The whole validation part in the results section now reads:

"**Validation of the metabolite-CHF links in cardiomyoblasts.** Differential expression analysis of Natriuretic Peptide B gene (NPPB), one of the key genes relevant for the pathogenesis of CHF, was investigated in H9c2 cardiomyoblasts (derived from rat heart tissue) by exposing to different metabolites. Ten metabolites representing distinct metabolic pathways were selected, each with two different doses, which include the mean and maximum concentrations measured in serum samples collected from mice with heart failure induced by transverse aortic constriction (n=4; Table EV5). After 12 hours of exposure, five metabolites including methylmalonic acid (biomarker of oxidative stress), succinic acid (involved in tricarboxylic acid cycle), ImP (from histidine metabolism), 2-hydroxyisobutyric acid (from BCAAs metabolism), and 3-hydroxybutyric acid (biomarker of lipid beta-oxidation), as expected, induced higher levels of NPPB expression compared to the phosphate-buffered saline (PBS) control; succinic acid and ImP even significantly

elevated NPPB expression levels in H9c2 cells pretreated with hypoxia/reoxygenation (H/R), an important cause of CHF (Figure 3A). In contrast, four metabolites, including phenylacetylglutamine, had no impact on NPPB expression, which seemed contradictory to a recent study indicating upregulation of this gene upon phenylacetylglutamine exposure (Romano et al, 2023). The study by Romano et al. used a dose of 100 μM, which was 1000 times higher than the doses used in this study (0.11 and 0.16 μM). When the dose of this metabolite was increased to the same level, NPPB expression was indeed significantly upregulated (Figure EV5), confirming a crucial dose effect. Glyceric acid was expected to have a protective role against CHF but failed to suppress NPPB expression in the H/R pretreated cells and might even cause damage to normal cardiomyoblasts. Additional JC-1 dye staining revealed that ImP disrupted cardiomyoblast functions, as indicated by significantly reduced mitochondrial membrane potential after exposure in both cell models (JC-1 red/green ratios; Figure 3B, C)."

As mentioned above, we have also tested representative metabolite from TCA cycle. We additionally discussed those metabolites as suggested by the viewer.

Related discussion now reads:

"…intermediates from incomplete FFA oxidation such as the main ketone body 3-hydroxybutyric acid and different carnitines, and those involved in the tricarboxylic acid cycle such as citric acid, isocitric acid, fumaric acid, aconitic acid and succinic acid, might represent more promising biomarkers for CHF development. For instance, elevated levels of citric acid and succinic acid have been associated with high risk of CHF (Bulló *et al*, 2021). Consistently, our cell experiments demonstrated that both 3-hydroxybutyric acid and succinic acid induced *NPPB* expression. Moreover, succinic acid could promote generation of reactive oxygen species (Mills *et al*, 2016), leading to myocardial cell deaths (Chouchani *et al*, 2014). However, it is essential to conduct further studies to determine whether increased circulation and utilization of those lipids represent compensatory mechanisms or true pathogenic factors for CHF and related comorbidities."

Referee #3 (Comments on Novelty/Model System for Author):

The manuscript is generally interesting. But the current version lacks sufficient details in methods for precise quality evaluation.

Response: We thank the reviewer for pointing this out. In response, we have made significant revisions to the Methods section to improve clarity. We have included all computational and experimental details, which are now described in detail below.

Referee #3 (Remarks for Author):

Summary:
Hua et al. report the alterations in gut microbially derived metabolites are linked to CHF and its two common comorbidities, that are type 2 diabetes and chronic kidney disease. The profiling of 151 metabolites in a cohort of 260 individuals were based on absolute quantification, which resulted in a big variation in the levels of measured metabolites across the cohort. The 98 CHF-associated metabolites were validated in external study materials that are geographically independent. In addition, the authors highlighted imidazole propionate, a bacterial metabolite with 3 folds of basal levels in Chinese than that in Swedish cohort. Finally, as the authors showed, metabolites-based biomarkers provide prognostic superiority over the traditional risk scores for CHF.

Response: We appreciate the reviewer for those positive comments.

Major comments:
1. Both discovery and replication study samples are of adequate size and as such, the epidemiological studies seem statistically powered. The presented observational and cross-sectional studies are however, purely descriptive and the interpretations of identified CHF-, T2D-, or CKD-related metabolites remain speculative. Substantial supportive evidence seems needed to interpret the reported relationships between gut microbially derived metabolites and onset of T2D, CHF, or CKD. Along the same line, the descriptive nature of the current studies would benefit from fecal microbiota transfer studies from individuals with CHF-, T2D, or CKD-phenotypes to germ-free rodents to explore mechanisms in depth behind potential inducible changes.

Response: Yes. We totally agree with the reviewer for this suggestion. Due to time and resource constraints, we opted to perform in vitro cell experiments to validate some of the metabolite-disease links. Specifically, we selected ten microbially associated metabolites that represented distinct pathways, with each metabolite tested at two different doses. To identify a reasonable dose for the cell-metabolite

coculturing experiments, we explored the physiological range of each metabolite in a TAC heart failure mice model. Overall, we confirmed six out of the ten metabolite-disease links in a dose-dependent manner (Figure 3 as shown below). We believe that these results partly address the reviewer's concern.

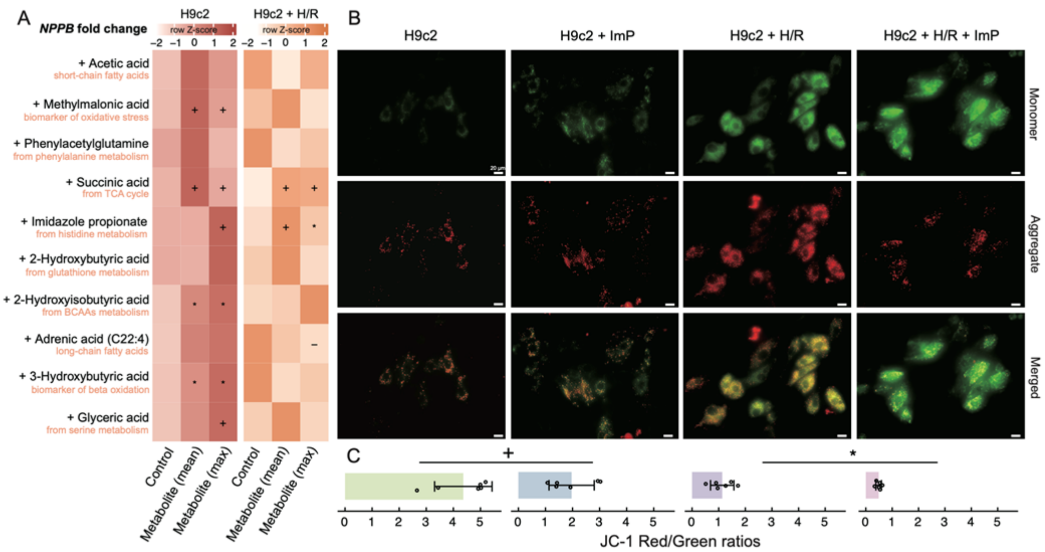

Figure 3. Cardiomyoblast-metabolite coculturing and induction of NPPB gene expression. A, the relative expression of NPPB (scaled by row) upon different metabolites exposure in H9c2 cells pretreated with or without hypoxia/reoxygenation (H/R; six replicates per group). B, representative JC-1 staining images showing red fluorescence of JC-1 aggregates and green signal of monomers. Scale bars: 20 µm. C, quantification of mitochondrial membrane potential (n=6). The data are shown as mean ± s.d; Wilcoxon rank-sum test (-P < 0.1, *P < 0.05, +P < 0.01).

The whole validation part in the results section now reads:

"**Validation of the metabolite-CHF links in cardiomyoblasts.** Differential expression analysis of Natriuretic Peptide B gene (NPPB), one of the key genes relevant for the pathogenesis of CHF, was investigated in H9c2 cardiomyoblasts (derived from rat heart tissue) by exposing to different metabolites. Ten metabolites representing distinct metabolic pathways were selected, each with two different doses, which include the mean and maximum concentrations measured in serum samples collected from mice with heart failure induced by transverse aortic constriction (n=4; Table EV5). After 12 hours of exposure, five metabolites including methylmalonic acid (biomarker of oxidative stress), succinic acid (involved in tricarboxylic acid cycle), ImP (from histidine metabolism), 2-hydroxyisobutyric acid (from BCAAs metabolism), and 3-hydroxybutyric acid (biomarker of lipid beta-oxidation), as expected, induced higher levels of NPPB expression compared to the

phosphate-buffered saline (PBS) control; succinic acid and ImP even significantly elevated NPPB expression levels in H9c2 cells pretreated with hypoxia/reoxygenation (H/R), an important cause of CHF (Figure 3A). In contrast, four metabolites, including phenylacetylglutamine, had no impact on NPPB expression, which seemed contradictory to a recent study indicating upregulation of this gene upon phenylacetylglutamine exposure (Romano et al, 2023). The study by Romano et al. used a dose of 100 μM, which was 1000 times higher than the doses used in this study (0.11 and 0.16 μM). When the dose of this metabolite was increased to the same level, NPPB expression was indeed significantly upregulated (Figure EV5), confirming a crucial dose effect. Glyceric acid was expected to have a protective role against CHF but failed to suppress NPPB expression in the H/R pretreated cells and might even cause damage to normal cardiomyoblasts. Additional JC-1 dye staining revealed that ImP disrupted cardiomyoblast functions, as indicated by significantly reduced mitochondrial membrane potential after exposure in both cell models (JC-1 red/green ratios; Figure 3B, C)."

2. It's unfortunate to miss the profile of bile acids (as far as I can see, only chenodeoxycholic acid, chenodeoxyglycocholic acid, and glycolithocholic acid were included), short chain fatty acids (only acetate was measured), and trimethylamine N-oxide (absent), which had been widely reported as microbiota-related metabolites linked to insulin resistance, T2D, or cardiometabolic diseases. Please clarify why these metabolites were omitted in the current work.

Response: We thank the reviewer for this comment. Overall, we detected 13 bile acids and nine SCFAs as shown in **Table EV1**. However, the majority of these metabolites were found to be insignificant in the disease groups compared to the control group, and therefore, were not included in the original version. TMAO, unfortunately, could not be detected by the current metabolomics platform.

3. It is well-known that multiple drugs and drug combinations cause considerable perturbation of gut microbiome composition and function. Although the investigators report that the study had excluded the effects of metformin on metabolome, but not clear how the impact of metformin was deconfounded. Still, such large number of individuals in real life settings both in discovery cohort undoubtedly take drugs including proton pump inhibitors, statins, antihypertensives, antinflammatory drugs, pain killers, Chinese herbal medicine etc, - all with potentially major confounding impact on microbiome related metabolites. Therefore, authors are strongly encouraged to provide information on medication use and to apply a state-of-the art drug-deconfounding pipeline in the analyses of their data.

Response: Yes, indeed. We have carefully sorted out all drugs taken in this cohort and applied a state-of-the-art drug-deconfounding pipeline (Forslund *et al*., 2021) to explore how current metabolite-disease links were affected by medication history. In total, we found 23 disease-metabolite links from 19 metabolites were potentially confounded by the taken drug and have been highlighted in the main **Figure 1** and **Table EV2**. We have also revised the text accordingly, which now reads:

"...In total, we identified 258 metabolite-disease links from 96 microbially associated metabolites, among which 23 such links from 19 metabolites were potentially confounded by medications based on a state-of-art drug-deconfounding pipline (Forslund et al, 2021) (Figure 1; Table EV2). For instance, associations between threonic acid and CHF and/or related comorbidities tend to be affected by diuretics, consist with previous findings (Forslund et al., 2021)."

And also in the study limitation part:

"...Third, some of the metabolite-disease links identified in our study were confounded by medications as shown... "

4. Current method lacks far less sufficient details. For example, page 11, how many individuals completed the follow up period? When was the plasma collected after obtaining blood samples from participants? How was the plasma stored? More importantly, how was the targeted metabolomics performed? The reference provided by the authors was about metabolomics profiling based on GC-Q/TOF-MS platform, whereas in the manuscript, they say a UPLC-MS/MS system was used. In statistical analysis, how was the p values adjusted? Without such details, it is impossible to evaluate the reliability of the presented outcomes.

Response: We apologize for those missing information. We have thoroughly revised the methods section accordingly.

The related part in the text now reads:
"…A total of 244 individuals completed the follow-up study with 89 CHF rehospitalization and 24 cardiovascular deaths reported…"

We would like to clarify that the metabolomics method has been recently updated and we mistakenly cited an old reference. We have now corrected this and revised the whole methods part as:

"**Targeted metabolomics profiling.** The blood samples were centrifuged at 1,000g for 10 min to separate the serum within 12 hours after collection, and the aliquoted serum samples were stored at -80 °C for further analysis. Absolute quantification of the microbially associated metabolites was conducted based on the Q300 platform

(Metabo-Profile Biotechnology, Shanghai, China) as previously described (Xie et al., 2021). In brief, 25 µL serum samples in a 96-well plate was mixed with 120 µL methanol and then vortexed vigorously for five minutes and centrifuged at 4,000g for 30 minutes. 30 µL of supernatant and 20 µL of freshly prepared derivative reagents were added to a clean 96-well plate for further derivatization. The samples were further diluted using 330 µL of ice-cod 50% methanol solution and centrifuged. 135 µL of supernatant was transferred to a new 96-well plate with 10 µL internal standards in each well. Measurement was performed using a UPLC-MS/MS system (ACQUITY UPLC-Xevo TQ-S, Waters Corp., Milford, MA, USA) with the following settings: BEHC18 1.7 µM VanGuard pre-column and analytical column; water with 0.1% formic acid for mobile phase A and acetonitrile/IPA for mobile phase B; flow rate at 0.4 mL/min; and capillary (Kv)=1.5 (ESI+), 2.0 (ESI-). The raw data files generated by UPLC-MS/MS were processed using the MassLynx software (v4.1) to perform peak integration, calibration, and quantification for each metabolite**.**"

We also revised the statistical analysis part to include more details as suggested by the reviewer:

"...Raw P values were adjusted using Benjamini-Hochberg correction (Benjamini & Hochberg, 1995). Adjusted P values < 0.1 and raw P values < 0.05 were considered statistically significant."

Minor comments:

1. The title should be more specified as this manuscript only talks about microbially related metabolites.

Response: We have revised accordingly. The title now reads: "Microbial metabolites in chronic heart failure and its common comorbidities".

2. Figure 2 legend, here raw p value threshold was set at 0.1, why not 0.05?

Response: We have now consistently changed to 0.05 but still kept ImP which showed marginal significance in NGT+CHF vs. Prediabetes+CHF (P = 0.055).

3. Figure 3A, how was the association analysis performed? Was gender adjusted?

Response: We apologize for the lack of clarity. We used random forest models to evaluate the association (R^2) of each metabolite with all clinical variables. Gender was also considered during the revised version. The methods part now reads:

"Statistical analyses. All statistical analyses were conducted in the R environment (version 4.0.4). The nonparametric Wilcox rank-sum test was used to identify

metabolites that differed significantly between groups. For categorical variables, the chi-squared test was used. Drug deconfounding analysis for the metabolite-disease links was performed using the metadeconfoundR package (v0.1.8) (Forslund et al., 2021). The multivariable Cox proportional hazards model (survival v3.2.13) (Therneau, 2019) and random forest model (randomForest v4.6.14) (Liaw & Wiener, 2002) with sex adjustment (ntree=3000, mtry=2-30) were utilized. Bi-directional mediation analysis (mediation v4.5.0) (Tingley et al, 2014) was performed with age and sex adjustment. Kaplan-Meier survival analysis (survminer v0.4.9) (Kassambara et al, 2021) and time-dependent ROC curves (riskRegression v2021.10.10) (Gerds & Kattan, 2021) were conducted with bootstrap resampling (n=1000). The log-rank test was applied to compare survival curves between groups. Raw P values were adjusted by the Benjamini-Hochberg method (Benjamini & Hochberg, 1995). Adjusted P values < 0.1 and raw P values < 0.05 were considered statistically significant."

4. Results should be shortened; the biological interpretation of disease-associated metabolites should be moved to discussion.

Response: Yes. We have revised accordingly and moved discussions about amino acid and lipid metabolism to the discussion section. All text changes were highlighted in red color in the main manuscript. We thank again the reviewer for all constructive comments.

14th Apr 2023

Dear Dr. Wu,

Thank you for the submission of your revised manuscript to EMBO Molecular Medicine. We have now received the reports from referees #1 and #3 who re-reviewed your manuscript. Additionally, both referees also evaluated your responses to referee #2. As you will see below, the referees are now supportive of publication pending minor revisions, and we will therefore be able to accept your manuscript once the following minor points will be addressed:

1/ Referees' comments: please address the remaining concerns from the referees.

2/ Main manuscript text:
- Please removed any coloured text and accept all changes. Only keep in track changes mode any new modification.
- In the abstract, please reformulate the following sentences to make them clearer: "In all three cohorts and diseases, 16 and 3 metabolites, including imidazole propionate, consistently differed, respectively. Notably, the basal levels of this metabolite were three times higher in the Chinese than that in the Swedish and increased 1.1-1.6 fold for each additional CHF comorbidity; further cell experiments confirmed its causal links to distinct CHF relevant phenotypes."
- Please remove the reference to the Graphical Abstract in the introduction. Also remove the headings "Study limitations" and "Conclusions" in the discussion.
- Materials and methods:
o Human subjects: Please include a sentence that the experiments conformed to the principles set out in the WMA Declaration of Helsinki and the Department of Health and Human Services Belmont Report.
o Cell cultures: please indicate whether the cells were tested for mycoplasma contamination (kindly also adjust the checklist accordingly).
o Statistics: please include a sentence about randomization, blinding and inclusion/exclusion criteria (please also provide matching information in the checklist).
- Data Availability section: thank you for providing access to the datasets produced in your study. We nevertheless ask authors to provide access to their raw data via deposition in a public repository (such as MetabolomicsWorkbench or MetaboLights). Please see also our guidelines for authors: https://www.embopress.org/page/journal/17574684/authorguide#dataavailability.
- Acknowledgements: Please make sure that the information provided match the funding information entered in the submission system (missing: 82270582, Program of Shanghai Academic Research Leader (21XD1402100), Shanghai Municipal Science and Technology Major Project (2017SHZDZX01), Shanghai Pujiang Program (21PJ1401200), the Youth Project of Shanghai Municipal Health Commission (20174Y0142), the Scholar Training Support (Grant No. 2019GG01) from Huangpu Health Commission (Shanghai), the Excellent Youth Project (Grant No. YQA2021003) of Ruijin Hospital/Lu wan branch, School of Medicine, Shanghai Jiaotong University, the China Postdoctoral Science Foundation (Grant No. 2022M710785)).

3/ Figures:
- Please make sure to provide exact p values for all your figures and EV figures (or figure legends).
- Please upload you EV Tables 1-5 as individual files, each with its corresponding legend.
- Thank you for providing Source Data. Please also fill in and provide the Source Data checklist.

4/ Please note that all corresponding authors are required to supply an ORCID ID for their name upon submission of a revised manuscript. An ORCID identified is currently missing for Wei Jin.

5/ Thank you for providing The Paper Explained. I introduced minor changes, please let me know if you agree or amend as you see fit:

PROBLEM: Whether and which circulating microbial metabolites are associated with chronic heart failure and related comorbidities is currently unknown.
RESULTS: An association between microbial metabolites and cardiometabolic diseases was identified in a Chinese cohort of 260 individuals with or without incidence of chronic heart failure and related comorbidities. Compared with the traditional heart failure risk score, the metabolite-based risk score exhibited superior performance for CHF prognosis.
IMPACT: These results revealed several potential microbial metabolites and pathways that could be utilized for chronic heart failure multimorbidity monitoring, targeted for drug design, and integrated for disease prognosis.

6/ Thank you for providing a nice synopsis image. Please also provide a synopsis text to further enhance discoverability. Synopses are displayed on the journal webpage and are freely accessible to all readers. They include a short stand first (maximum of 300 characters, including space) as well as 2-5 one-sentences bullet points that summarizes the paper. Please write the bullet points to summarize the key NEW findings. They should be designed to be complementary to the abstract - i.e. not repeat the same text. We encourage inclusion of key acronyms and quantitative information (maximum of 30 words / bullet point). Please use the passive voice.

7/ As part of the EMBO Publications transparent editorial process initiative (see our Editorial at http://embomolmed.embopress.org/content/2/9/329), EMBO Molecular Medicine will publish online a Review Process File (RPF) to accompany accepted manuscripts.
This file will be published in conjunction with your paper and will include the anonymous referee reports, your point-by-point response and all pertinent correspondence relating to the manuscript. Let us know whether you agree with the publication of the RPF and as here, if you want to remove or not any figures from it prior to publication.
Please note that the Authors checklist will be published at the end of the RPF.

I look forward to receiving your revised manuscript.

Yours sincerely,

Lise Roth

Lise Roth, PhD
Senior Editor
EMBO Molecular Medicine

***** Reviewer's comments *****

Referee #1 (Remarks for Author):

I thank the authors for addressing my comments, I believe the manuscript has improved a lot and is suitable for publication.

A minor comment, I would in general refrain from stating that any analysis or data confirms causality but rather supports it.

Referee #3 (Remarks for Author):

All the concerns from this reviewer have been adequately addressed and the manuscript has been greatly improved. Yet, a few concerns persist,

1) it is unclear why the authors defined doses of microbial metabolites in mice model while co-cultured the 10 selected metabolites with a cell line derived from rats;

2) please in the methods section (page 17), clarify what buffer was used for dissolving the 10 selected microbial metabolites as adrenic acid (C22:4) is considered as practically insoluble in water;

3) Revise 'cell experiments' to 'cellular experiments' throughout the manuscript.

***** Reviewer's comments *****

Referee #1 (Remarks for Author):

I thank the authors for addressing my comments, I believe the manuscript has improved a lot and is suitable for publication.

Response: We thank the reviewer for all the valuable feedback and support.

A minor comment, I would in general refrain from stating that any analysis or data confirms causality but rather supports it.

Response: Thanks! We have revised accordingly.

Referee #3 (Remarks for Author):

All the concerns from this reviewer have been adequately addressed and the manuscript has been greatly improved. Yet, a few concerns persist,

Response: We sincerely appreciate your time and efforts in helping us improve our manuscript.

1) it is unclear why the authors defined doses of microbial metabolites in mice model while co-cultured the 10 selected metabolites with a cell line derived from rats;

Response: We thank the reviewer for pointing this out. H9c2 cell lines, derived from rat models, are widely utilized in mechanistic studies of heart failure and were therefore used in this study. However, we routinely use C57/BL6 mice in our group for the TAC model.

2) please in the methods section (page 17), clarify what buffer was used for dissolving the 10 selected microbial metabolites as adrenic acid (C22:4) is considered as practically insoluble in water;

Response: We apologize for those missing information. We have revised the manuscript for more clarity:

"...All metabolites were dissolved in phosphate-buffered saline (PBS) for subsequent use, with the exception of adrenic acid (C22:4) which was dissolved in ethanol first before being diluted in PBS."

3) Revise 'cell experiments' to 'cellular experiments' throughout the manuscript.

Response: Yes. We have revised accordingly.

18th Apr 2023

Dear Dr. Wu,

Thank you for providing the revised files. I am pleased to inform you that your manuscript is accepted for publication and is now being sent to our publisher to be included in the next available issue of EMBO Molecular Medicine!

We would like to remind you that as part of the EMBO Publications transparent editorial process initiative, EMBO Molecular Medicine will publish a Review Process File online to accompany accepted manuscripts. If you do NOT want the file to be published or would like to exclude figures, please immediately inform the editorial office via e-mail.

Please read below for additional IMPORTANT information regarding your article, its publication and the production process.

Congratulations on your interesting work,

With kind regards,

Lise Roth

Lise Roth, Ph.D
Senior Editor
EMBO Molecular Medicine

Follow us on Twitter @EmboMolMed
Sign up for eTOCs at embopress.org/alertsfeeds

*** *** *** IMPORTANT INFORMATION *** *** ***

SPEED OF PUBLICATION
The journal aims for rapid publication of papers, using using the advance online publication "Early View" to expedite the process: A properly copy-edited and formatted version will be published as "Early View" after the proofs have been corrected. Please help the Editors and publisher avoid delays by providing e-mail address(es), telephone and fax numbers at which author(s) can be contacted.

Should you be planning a Press Release on your article, please get in contact with embomolmed@wiley.com as early as possible, in order to coordinate publication and release dates.

LICENSE AND PAYMENT:

All articles published in EMBO Molecular Medicine are fully open access: immediately and freely available to read, download and share.

EMBO Molecular Medicine charges an article processing charge (APC) to cover the publication costs. You, as the corresponding author for this manuscript, should have already received a quote with the article processing fee separately. Please let us know in case this quote has not been received.

Once your article is at Wiley for editorial production you will receive an email from Wiley's Author Services system, which will ask you to log in and will present you with the publication license form for completion. Within the same system the publication fee can be paid by credit card, an invoice, pro forma invoice or purchase order can be requested.

Payment of the publication charge and the signed Open Access Agreement form must be received before the article can be published online.

PROOFS

You will receive the proofs by e-mail approximately 2 weeks after all relevant files have been sent o our Production Office.

Please return them within 48 hours and if there should be any problems, please contact the production office at embopressproduction@wiley.com.

Please inform us if there is likely to be any difficulty in reaching you at the above address at that time. Failure to meet our deadlines may result in a delay of publication.

All further communications concerning your paper proofs should quote reference number EMM-2022-16928-V3 and be directed to the production office at embopressproduction@wiley.com.

EMBO Press Author Checklist

Corresponding Author Name: Hao Wu and Wei Jin	
Journal Submitted to: EMBO Molecular Medicine	
Manuscript Number: EMM-2022-16928	

USEFUL LINKS FOR COMPLETING THIS FORM
The EMBO Journal - Author Guidelines
EMBO Reports - Author Guidelines
Molecular Systems Biology - Author Guidelines
EMBO Molecular Medicine - Author Guidelines

Reporting Checklist for Life Science Articles (updated January
This checklist is adapted from Materials Design Analysis Reporting (MDAR) Checklist for Authors. MDAR establishes a minimum set of requirements in transparent reporting in the life sciences (see Statement of Task: 10.31222/osf.io/9sm4x). Please follow the journal's guidelines in preparing your
Please note that a copy of this checklist will be published alongside your article.

Abridged guidelines for figures
1. Data
The data shown in figures should satisfy the following conditions:
- the data were obtained and processed according to the field's best practice and are presented to reflect the results of the experiments in an accurate and unbiased manner.
- ideally, figure panels should include only measurements that are directly comparable to each other and obtained with the same assay.
- plots include clearly labeled error bars for independent experiments and sample sizes. Unless justified, error bars should not be shown for technical
- if n<5, the individual data points from each experiment should be plotted. Any statistical test employed should be justified.
- Source Data should be included to report the data underlying figures according to the guidelines set out in the authorship guidelines on Data

2. Captions
Each figure caption should contain the following information, for each panel where they are relevant:
- a specification of the experimental system investigated (eg cell line, species name).
- the assay(s) and method(s) used to carry out the reported observations and measurements.
- an explicit mention of the biological and chemical entity(ies) that are being measured.
- an explicit mention of the biological and chemical entity(ies) that are altered/varied/perturbed in a controlled manner.
- the exact sample size (n) for each experimental group/condition, given as a number, not a range;
- a description of the sample collection allowing the reader to understand whether the samples represent technical or biological replicates (including how many animals, litters, cultures, etc.).
- a statement of how many times the experiment shown was independently replicated in the laboratory.
- definitions of statistical methods and measures:
  - common tests, such as t-test (please specify whether paired vs. unpaired), simple χ2 tests, Wilcoxon and Mann-Whitney tests, can be unambiguously identified by name only, but more complex techniques should be described in the methods section;
  - are tests one-sided or two-sided?
  - are there adjustments for multiple comparisons?
  - exact statistical test results, e.g., P values = x but not P values < x;
  - definition of 'center values' as median or average;
  - definition of error bars as s.d. or s.e.m.

Please complete ALL of the questions below.
Select "Not Applicable" only when the requested information is not relevant for your study.

Materials

Newly Created Materials	Information included in the manuscript?	In which section is the information available? (Reagents and Tools Table, Materials and Methods, Figures, Data Availability Section)
New materials and reagents need to be available; do any restrictions apply?	Yes	Methods

Antibodies	Information included in the manuscript?	In which section is the information available? (Reagents and Tools Table, Materials and Methods, Figures, Data Availability Section)
For **antibodies** provide the following information:<br>- Commercial antibodies: RRID (if possible) or supplier name, catalogue number and or/clone number<br>- Non-commercial: RRID or citation	Not Applicable	

DNA and RNA sequences	Information included in the manuscript?	In which section is the information available? (Reagents and Tools Table, Materials and Methods, Figures, Data Availability Section)
**Short novel DNA or RNA including primers, probes**: provide the sequences.	Yes	Methods

Cell materials	Information included in the manuscript?	In which section is the information available? (Reagents and Tools Table, Materials and Methods, Figures, Data Availability Section)
**Cell lines:** Provide species information, strain. Provide accession number in repository **OR** supplier name, catalog number, clone number, and/**OR** RRID.	Yes	Methods
**Primary cultures:** Provide species, strain, sex of origin, genetic modification status.	Yes	Methods
Report if the cell lines were recently **authenticated** (e.g., by STR profiling) and tested for mycoplasma contamination.	Yes	Methods

Experimental animals	Information included in the manuscript?	In which section is the information available? (Reagents and Tools Table, Materials and Methods, Figures, Data Availability Section)
**Laboratory animals or Model organisms:** Provide species, strain, sex, age, genetic modification status. Provide accession number in repository **OR** supplier name, catalog number, clone number, **OR** RRID.	Yes	Methods
**Animal observed in or captured from the field:** Provide species, sex, and age where possible.	Not Applicable	
Please detail **housing and husbandry conditions**.	Yes	Methods

Plants and microbes	Information included in the manuscript?	In which section is the information available? (Reagents and Tools Table, Materials and Methods, Figures, Data Availability Section)
**Plants:** provide species and strain, ecotype and cultivar where relevant, unique accession number if available, and source (including location for collected wild specimens).	Not Applicable	
**Microbes:** provide species and strain, unique accession number if available, and source.	Not Applicable	

Human research participants	Information included in the manuscript?	In which section is the information available? (Reagents and Tools Table, Materials and Methods, Figures, Data Availability Section)
If collected and within the bounds of privacy constraints report on age, sex and gender or ethnicity for all study participants.	Yes	Methods

Core facilities	Information included in the manuscript?	In which section is the information available? (Reagents and Tools Table, Materials and Methods, Figures, Data Availability Section)
If your work benefited from core facilities, was their service mentioned in the acknowledgments section?	Not Applicable	

Design

Study protocol	Information included in the manuscript?	In which section is the information available? (Reagents and Tools Table, Materials and Methods, Figures, Data Availability Section)

If study protocol has been **pre-registered, provide DOI in the manuscript**. For clinical trials, provide the trial registration number **OR** cite DOI.	Not Applicable	
Report the **clinical trial registration number** (at ClinicalTrials.gov or equivalent), where applicable.	Yes	Methods section; Identifier: NCT02998788

Laboratory protocol	Information included in the manuscript?	In which section is the information available? (Reagents and Tools Table, Materials and Methods, Figures, Data Availability Section)
Provide DOI OR other citation details if **external detailed step-by-step protocols** are available.	Not Applicable	

Experimental study design and statistics	Information included in the manuscript?	In which section is the information available? (Reagents and Tools Table, Materials and Methods, Figures, Data Availability Section)
Include a statement about **sample size** estimate even if no statistical methods were used.	Not Applicable	
Were any steps taken to minimize the effects of subjective bias when allocating animals/samples to treatment (e.g. **randomization procedure**)? If yes, have they been described?	Yes	Methods
Include a statement about **blinding** even if no blinding was done.	Yes	Methods
Describe **inclusion/exclusion criteria** if samples or animals were excluded from the analysis. Were the criteria pre-established?<br><br>If sample or data points were omitted from analysis, report if this was due to attrition or intentional exclusion and provide justification.	Yes	Methods
For every figure, are **statistical tests** justified as appropriate? Do the data meet the assumptions of the tests (e.g., normal distribution)? Describe any methods used to assess it. Is there an estimate of variation within each group of data? Is the variance similar between the groups that are being statistically compared?	Yes	Methods

Sample definition and in-laboratory replication	Information included in the manuscript?	In which section is the information available? (Reagents and Tools Table, Materials and Methods, Figures, Data Availability Section)
In the figure legends: state number of times the experiment was **replicated** in laboratory.	Yes	Figure Legends and Methods
In the figure legends: define whether data describe **technical or biological replicates**.	Yes	Figure Legends and Methods

Ethics

Ethics	Information included in the manuscript?	In which section is the information available? (Reagents and Tools Table, Materials and Methods, Figures, Data Availability Section)
Studies involving **human participants**: State details of **authority granting ethics approval** (IRB or equivalent committee(s), provide reference number for approval.	Yes	Methods
Studies involving **human participants**: Include a statement confirming that **informed consent** was obtained from all subjects and that the experiments conformed to the principles set out in the WMA Declaration of Helsinki and the Department of Health and Human Services Belmont Report.	Yes	Methods
Studies involving **human participants:** For publication of **patient photos**, include a statement confirming that consent to publish was obtained.	Not Applicable	
Studies involving experimental **animals**: State details of **authority granting ethics approval** (IRB or equivalent committee(s), provide reference number for approval. Include a statement of compliance with ethical regulations.	Yes	Methods
Studies involving **specimen and field samples:** State if relevant **permits** obtained, provide details of authority approving study; if none were required, explain why.	Not Applicable	

Dual Use Research of Concern (DURC)	Information included in the manuscript?	In which section is the information available? (Reagents and Tools Table, Materials and Methods, Figures, Data Availability Section)
Could your study fall under dual use research restrictions? Please check biosecurity documents and list of **select agents and toxins** (CDC): https://www.selectagents.gov/sat/list.htm	Not Applicable	
If you used a select agent, is the security level of the lab appropriate and reported in the manuscript?	Not Applicable	
If a study is subject to dual use research of concern regulations, is the name of the **authority granting approval and reference number** for the regulatory approval provided in the manuscript?	Not Applicable	

Reporting

The MDAR framework recommends adoption of discipline-specific guidelines, established and endorsed through community initiatives. Journals have their own policy about requiring specific guidelines and recommendations to complement MDAR.

Adherence to community standards	Information included in the manuscript?	In which section is the information available? (Reagents and Tools Table, Materials and Methods, Figures, Data Availability Section)
State if relevant guidelines or checklists (e.g., **ICMJE, MIBBI, ARRIVE, PRISMA**) have been followed or provided.	Not Applicable	
For **tumor marker prognostic studies,** we recommend that you follow the **REMARK** reporting guidelines (see link list at top right). See author guidelines, under 'Reporting Guidelines'. Please confirm you have followed these guidelines.	Not Applicable	
For **phase II and III randomized controlled trials**, please refer to the **CONSORT** flow diagram (see link list at top right) and submit the CONSORT checklist (see link list at top right) with your submission. See author guidelines, under 'Reporting Guidelines'. Please confirm you have submitted this list.	Not Applicable	

Data Availability

Data availability	Information included in the manuscript?	In which section is the information available? (Reagents and Tools Table, Materials and Methods, Figures, Data Availability Section)
Have **primary datasets** been deposited according to the journal's guidelines (see 'Data Deposition' section) and the respective accession numbers provided in the Data Availability Section?	Yes	Data Availability Section
Were **human clinical and genomic datasets** deposited in a public access-controlled repository in accordance to ethical obligations to the patients and to the applicable consent agreement?	Not Applicable	
Are **computational models** that are central and integral to a study available without restrictions in a machine-readable form? Were the relevant accession numbers or links provided?	Yes	Data Availability Section
If publicly available data were reused, provide the respective **data citations in the reference list**.	Yes	Methods
